# The robustness of polymer-based lateral flow glycoassays in medically relevant media

**DOI:** 10.1039/d6lp00336b

**Published:** 2026-10-22

**Authors:** Megan Ellis, Zaimire Maimaitiaili, Douglas E. Soutar, Alexander J. Cantor, Jack C. Oram, Anish Mistry, Garrett D. Jackson, Alexander N. Baker

**Affiliations:** a Department of Chemistry, University of Warwick Gibbet Hill Road CV4 7AL Coventry UK alexander.baker@warwick.ac.uk

## Abstract

Lateral flow immunoassays are a commonly used tool in rapid point-of-care diagnostics, notably in the formats of pregnancy and COVID-19 tests. Their reliance on antibodies limits the adaptability of the assay however, due to complex manufacturing/development and sensitivity to biological media. Polymer-based alternatives, *i.e.* those that do not depend on antibody–antigen interactions, offer greater tunability and robustness, and are therefore highly desirable. Lateral flow glycoassays represent a new approach, utilising glycan-functionalised polymers as the recognition unit instead of antibodies. In this study, the effect of biologically relevant media (saliva, blood, urine), lectin concentration, and polymer length, on the performance of lateral flow and flow-through glycoassays is investigated. Poly(hydroxyethyl acrylamide) (PHEA) polymers of varying chain lengths were functionalised with galactosamine to produce glycopolymers. These were subsequently bound to gold nanoparticles. In both cases, lateral flow and flow-through, target lectins were successfully detected in a range of concentrations of biologically relevant media (saliva, blood, urine). Each system was tested using soybean agglutinin (SBA) as the target protein, a lectin with known selectivity for galactose derivatives, and bovine serum albumin (BSA) as an off-target control protein. A concentration range of 0.1 mg mL^−1^ to 0.5 mg mL^−1^ was explored for SBA in flow-through, and 0.1 mg mL^−1^ in lateral flow. Bovine serum albumin (BSA) was used as the control throughout the study at a concentration of 0.5 mg mL^−1^. The ability to modify assay performance through chain length demonstrates the inherent versatility, and more importantly tuneability, of glycopolymer based lateral flow devices. This highlights the robustness of the system in biologically relevant media and further emphasises the potential of lateral flow glycoassays (LFGAs) as adaptable, polymer-centered diagnostic platforms for improved point-of-care applications.

## Abstract 2

Lateral flow devices are common as rapid point-of-care diagnostics. Lateral Flow GlycoAssays (LFGAs) are emerging in this space thanks to their robustness in medically relevant media as demonstrated here.

## Graphical abstract

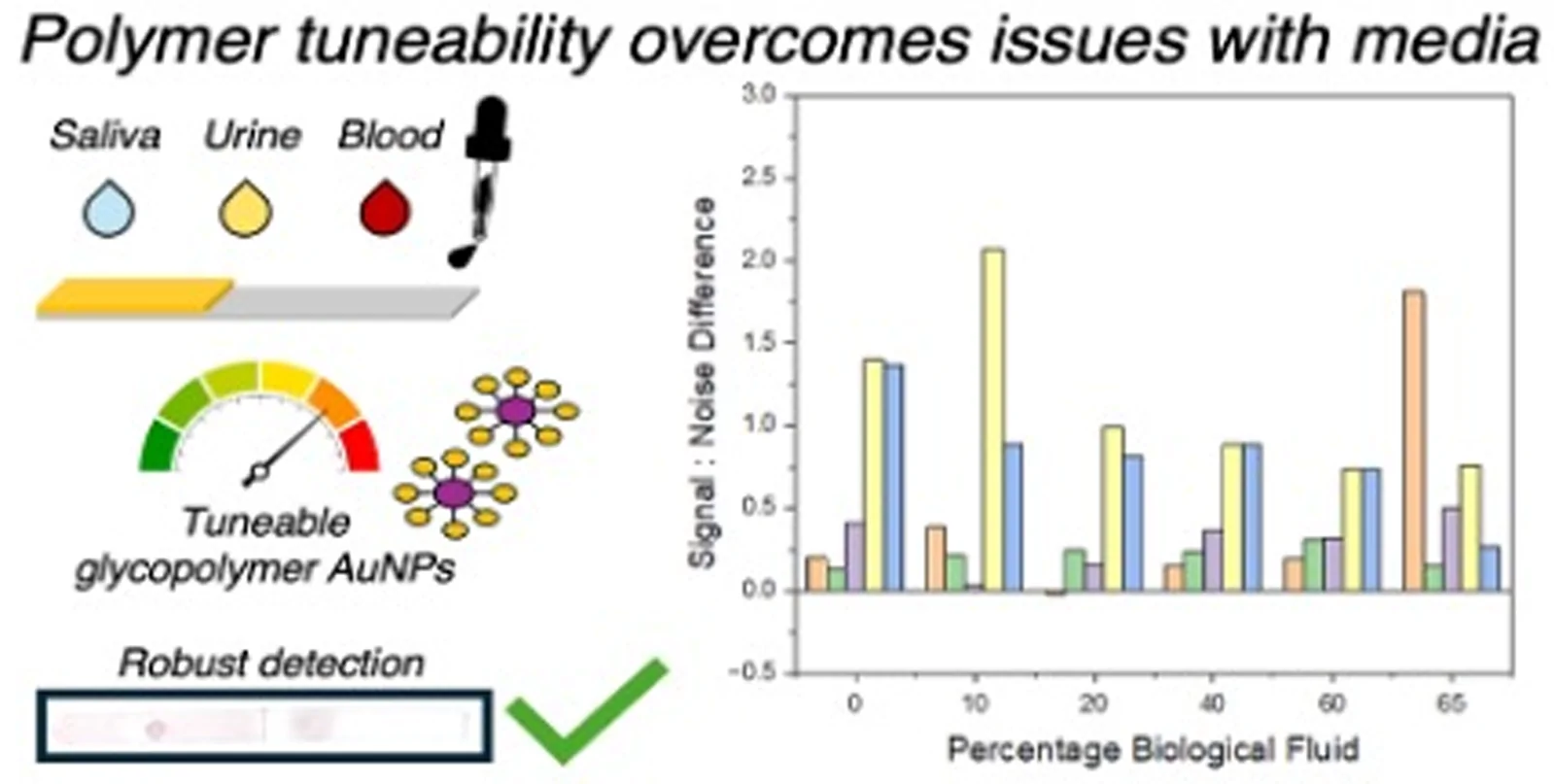

## Introduction

Point-of-care tests (POCTs) have emerged as alternatives to conventional laboratory-based diagnostics by enabling testing at or near the site of patient care.^1^ POCTs provide rapid turnaround times compared with standard laboratory testing, which requires trained personnel and specialised infrastructure, including large analytical instruments. These requirements present substantial barriers in low-resource settings. The disparity between laboratory-dependent diagnostics and the demand for accessible testing has driven significant growth in the POCT market.^2–4^ POCTs offer low-cost, self-contained platforms for the detection and control of infectious diseases – a need that is increasingly urgent given the projected rise in the frequency and risk of epidemics and pandemics.^5^ Furthermore the POCT market was valued at ∼$48 billion USD in 2024 with a projected valuation of ∼$68 billion USD by 2030,^6^ suggesting that this method of diagnostic testing will become even more prominent in the coming years.^7^

Lateral flow devices (LFDs), low-cost paper-based POCTs, provide simple and rapid detection making them common in clinical and public health settings.^8^ LFDs do not require specialised staff and testing facilities, making them invaluable for the diagnosing of various infectious diseases in less economically developed countries (LEDCs) or sparsely populated and remote regions where access to healthcare and/or resources is limited.^9^ This was epitomised by the Ebola epidemic in West Africa during 2014–15 wherein the diagnosis, originally, was made through reverse transcription-PCR (RT-PCR).^10^ These diagnostics were shown to be slow and expensive, taking 2–6 hours at a cost of $100 USD per test in comparison to LFD testing kits that cost $0.10 to $3.00 to manufacture.^10,11^ Additionally, blood storage and transportation required cold chain preservation; an uninterrupted temperature controlled supply chain from the moment of production, posing a problem in certain areas of West Africa, such as Guinea and Sierra Leone. These limitations led to the World Health Organization (WHO) calling for fast and inexpensive test systems that do not require specialist equipment or aforementioned cold chain preservation, leading to the rise and development of LFDs for the detection of Ebola antigen ReEBOV VP40.^12,13^

Though after the COVID-19 pandemic, there was substantial improvements made for RT-PCR including lower cost ($10–15) per test and quicker turnaround times (60–120 minutes) compared to during the Ebola epidemic.^14^ Studies done after the pandemic still highlighted RT-PCR's issue with cold chain preservation,^14^ and that countries, such as Mozambique, were paying $39.20 per test due to the high cost of medical supplies needed followed by the need for trained personnel.^15^

Lateral Flow Immunoassays (LFIAs) are the most commonly used LFDs, where antibodies are utilised as the recognition element, making use of an antibody–antigen interaction.^16^ LFIAs operate using a well-established principle where the paper based chromatographic system uses capillary action to flow a liquid sample, containing the analyte, through the strip across various functional zones.^16^ An extensive review of LFIAs and their underlying procedures is provided by Koczula *et al.* and Pedreira-Rincón *et al.* for reader comprehension.^3,17^ The attractiveness of LFIAs lies in their portability, low-cost, rapid detection, and ease of use, compared to laboratory-based analysers (previously discussed). Moreover, LFIAs provide high analytical sensitivity and specificity, as well as the user-friendly detection of results provided by the gold nanoparticles which act as a visual signal, again proving useful in low resource settings, small hospitals, and with monitoring health conditions.^3^ Additionally, compared to methods such as RT-PCR that have an average turnaround time of 60–120 minutes, LFIA results can be read between 5–30 minutes depending on the test itself.^11^

While LFIAs can be utilised to detect numerous targets, they are hampered by complex manufacturing/development, stability, and effect of modification on the antibody.^18^ As has been shown previously the antibody needs to be carefully designed and highly purified.^19^ While the robustness of LFIAs in different biological media, such as complex matrices like blood and urine, has also been called into question.^20^ For example, blood has been shown to interfere with LFIA visual assessment, due to its intense red colour and high viscosity.^20,21^ It has also been reported that pre-treatment is often required to separate blood cells from blood samples before LFIAs are used, thus increasing the time and cost of utilising this type of LFD.^16^ In related work, Merkoci *et al.* developed a promising strategy to apply electrophoresis to the LFIA strip to mitigate these issues. While many commercial LFIAs rely on sample dilution combined with specialised plasma separation membranes,^22^ as further discussed by Baillargeon *et al.*^23^ It is important to note, that the need for matrix dilution leads to a reduction in analyte concentration which in turn can affect the limit of detection.^23^ In summary although, LFIAs are well-established, many challenges still remain in maintaining the promise of their low cost and rapid testing. LFD research should develop more tuneable systems, *i.e.* in different biological fluids or utilising different recognition elements that are able to bind effectively under various conditions.

Lateral flow glycoassays (LFGAs) present a novel approach to address issues in conventional antibody-based systems. While antibodies are the most common recognition element employed in LFDs,^24–28^ there are alternatives (nucleotides,^29^ glycans, and lectins^30^*etc*.) that can be employed in LFD design.^28^ The diverse biological recognition processes mediated by glycans provides numerous opportunities to target either the glycans themselves or lectins (glycan binding proteins).^31^ Damborsky *et al.* demonstrated how lectins could be used as the capture unit in an LFD system.^30^ They developed a lectin-based LFD for the glycoprofiling of serum proteins such as prostate specific antigen (PSA), a widely used biomarker of prostate cancer. Work by Bayoumy *et al.* has also utilised glycans as the analyte, employing antibodies to target and detect specific glycan structures.^32^

LFGAs represent a new approach to replace immunoassays in which glycans are used as the capture units, in comparison to previous work utilising glycans as the analyte. Glycans, which hold a high affinity for glycan-binding proteins, can be conjugated onto polymers, for example, to produce glycopolymers of various chain lengths which are further immobilised onto gold nanoparticles to provide the recognition mechanism.^33,34^ LFGAs have successfully demonstrated the detection of lectin analytes; soybean agglutinin and Ricinus communis agglutinin I.^28^ As well, LFGAs results can be read in 20 minutes, based on the group's previous work, which has a similar turnaround time to LFIAs that take between 5–30 minutes.^11^ While LFGAs cannot be directly compared to LFIAs in terms of error when reviewing results, the limit of detection (LoD) has been shown to be comparable to LFIAs based on previous research by the group which concluded a LoD of 0.02 mg mL^−1^, similar to that of a commercial pregnancy test.^28^ Therefore, displaying the glycans used in this way can achieve the necessary LoD to be a viable alternative to antibody-based LFDs.

In these tests, signal to noise ratios were found to be dependent on nanoparticle structural parameters, such as the glycan, the precise chain length of the glycopolymer and the gold nanoparticle diameter. Notably, the chain length used was different for the lectins targeted, and following optimisation lectins were detected at sub-nanomolar concentrations, comparable to antibody-based LFIAs. Further work focusing on LFGAs explored the interaction between the SARS-CoV-2 spike protein S1 unit and sialic acids in LFGAs.^27^ Leading to *N*-acetyl neuraminic acid terminated polymer ligands being immobilised on gold nanoparticles, demonstrating that the SARS-CoV-2 spike protein could be detected in an LFGA, using a bovine serum albumin (BSA) glycoconjugate as the test line.^27,28^ Furthermore, flow-through assays developed from this could sense for SARS-CoV-2 in saliva samples from real patients. Thus, previous work has demonstrated that LFDs that use glycans as capture agents have strong potential as rapid diagnostics and are inherently tuneable to provide optimal signal to noise ratios.^25^

LFGAs can exploit multivalent carbohydrate–lectin interactions that can remain effective at lower analyte concentrations, an added positive compared to LFIAs.^35,36^ Although each glycopolymer presents a single carbohydrate ligand, lectins being multimers naturally hold several recognition sites which allow for the simultaneous binding to multiple glycans displayed across neighbouring polymer chains.^37^ This local glycan binding density gives rise to statistical rebinding, enhancing apparent binding strength, enabling effective lectin capture even at low analyte concentrations. This is especially significant when utilising urine as a biological fluid for the device matrix, where important biomarkers such as proteins and hormones are significantly diluted and thus fall below the concentration threshold of some conventional LFIAs.^38,39^ Due to lateral flow tests requiring a minimum concentration to produce a visible signal, urine biomarkers have been shown to be missed causing false negatives, reducing clinical reliability. To mitigate this issue, techniques and approaches such as osmotic processing have been reported to increase the concentration of urine biomarkers.^39^ LFGAs can circumvent dilution issues, and necessary mitigations, due to the nature of statistical rebinding and the cluster glycoside effect.^40^

Therefore, guided by previous LFGA research, this paper investigates the effects of varying concentrations of biologically relevant media (defibrinated sheep's blood, artificial saliva, and artificial urine), and the effects of lectin (soybean agglutinin, SBA) concentration on polymer-based glycoassay performance in both lateral flow and flow-through formats. Each system was tested using SBA as the target protein, a lectin with known selectivity for galactose derivatives, and bovine serum albumin (BSA) as an off-target control protein that when conjugated with glycans is often used as a test line in LFGAs. A concentration range of 0.1 mg mL^−1^ to 0.5 mg mL^−1^ was explored for SBA in flow-through, and 0.1 mg mL^−1^ in lateral flow. Bovine serum albumin (BSA) was used as the control throughout the study at a concentration of 0.5 mg mL^−1^.

This study demonstrates how LFGAs are a viable option for use in rapid detection, providing benefits *versus* LFIAs through tuning the polymer chain length to increase the signal to noise ratio depending on the biological fluid matrix in use which is simple and cost effective.

## Results and discussion

The primary aim of this work was to evaluate how polymer chain length, analyte concentration, and the presence of biological media affected glycoassay performance output, allowing for the exploration of the robustness of polymer-based LFGAs. Wherein robustness is defined quantitatively as the “degree to which a system or component can function correctly in the presence of invalid inputs or stressful environmental conditions”.^41^ To achieve this, two particle systems based on previous research, were used: Gal-PHEA_40_@AuNP_16_ and Gal-PHEA_72_@AuNP_16_ (where the polymer is functionalised onto the gold nanoparticle surface and denoted as polymer@nanoparticle).^28^ For ease of comparison of polymer length to previous papers, poly(hydroxyethyl acrylamides) (PHEAs) are named based on their length according to Size Exclusion Chromatography (SEC) in *N*,*N*-dimethylformamide (DMF) with poly(methyl methacrylate) (PMMA) calibrants.^42^

PHEA was chosen as the optimal polymer due to its solubility and colloidal stability when immobilised onto gold nanoparticles (AuNPs). Previous research from Baker *et al.* arrived at the conclusion that optimal AuNP diameter and polymer lengths for an LFGA *versus* SBA (soybean agglutinin), was an AuNP diameter of 16 nm and PHEA chain lengths of PHEA_40_ and PHEA_72_ as they provided optimal performance against SBA in terms of higher signal intensity, with for example Gal-PHEA_72_@AuNP_16_ having the highest signal to noise ratio (SNR).^28^ This previous study demonstrated that; (i) decreasing the density of the glycan led to decreased binding to SBA but also led to increased non-specific binding, (ii) increasing AuNP diameter led to increased signal intensity but also increased background noise, (iii) as polymer chain length increased, binding to SBA decreased and (iv) as polymer chain length decreased, binding to SBA and off-target binding increased.^28^ Considering this, the particle used for testing of robustness of our LFGAs were 100% glycan functionalised AuNP_16_ utilising a PHEA_72_ polymer. A PHEA_40_ system was also synthesised as it was hypothesised that the matrices would decrease SBA binding so a higher binding particle and therefore shorter polymer would be beneficial. Consequently Gal-PHEA_40_@AuNP_16_ and Gal-PHEA_72_@AuNP_16_ were synthesised.

Reversible addition–fragmentation chain transfer (RAFT) polymerisation was used to synthesise PHEA using pentafluorophenyl-2-(dodecylthiocarbonothioy)-2-methylpropanoate (PFP-DMP) as the RAFT agent to install a pentafluorophenyl group at the α-chain end and a trithiocarbonate at the ω-end for AuNP immobilisation (method found in SI). Galactosamine (2-deoxy-2-amino-galactose) was conjugated to the polymer *via* an amide linkage on C2, by displacement of the PFP end-group, to mimic the structure of *N*-acetylgalactosamine (GalNAc) a glycan with known affinity for SBA (target lectin),^43^ (Fig. 1) (method found in SI). Successful synthesis of PHEA_40_, PHEA_72_ (Table 1), Gal-PHEA_40_, and Gal-PHEA_72_, were confirmed by ^1^H NMR and SEC (SI Fig. S1 and Table S1).

**Fig. 1:**
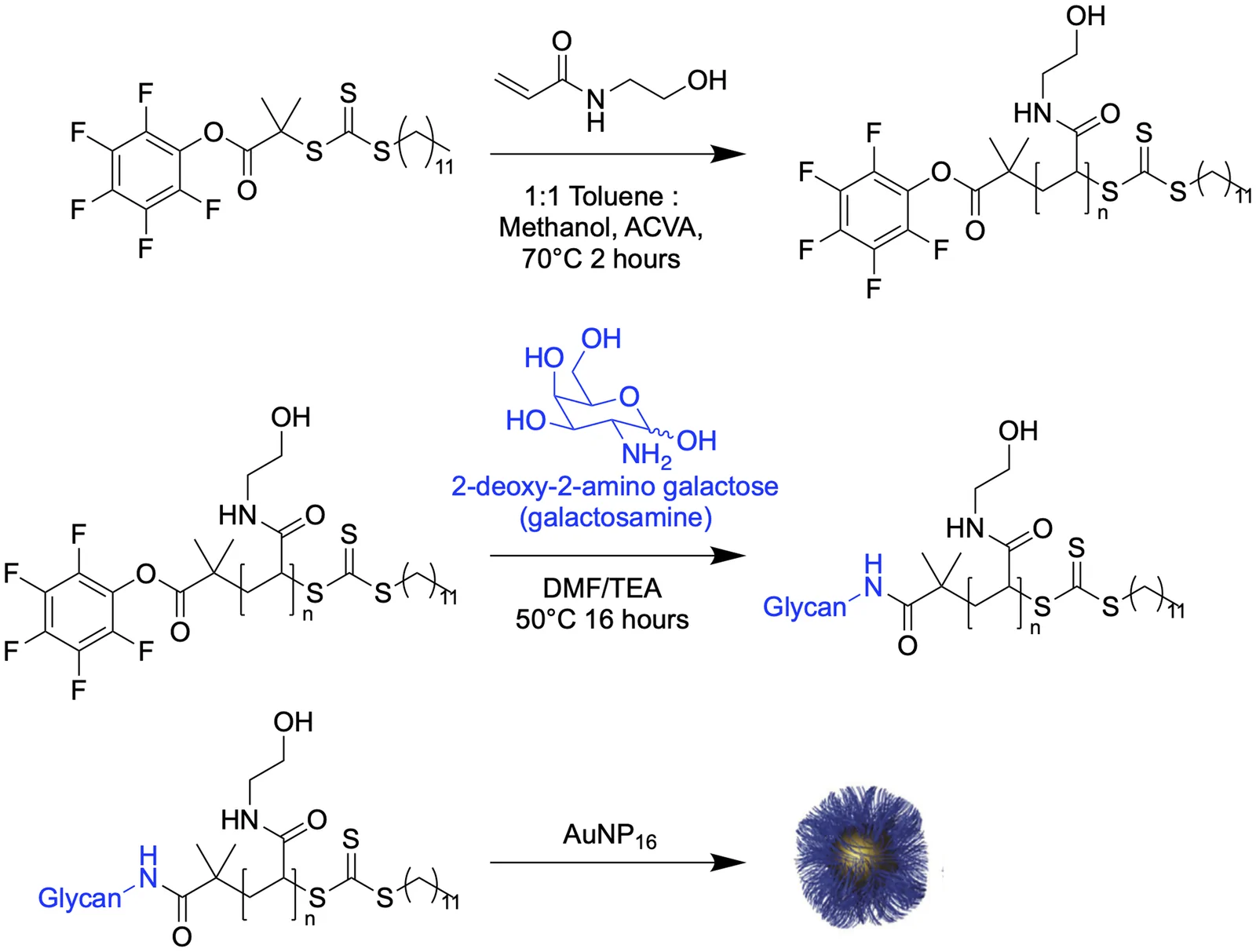
Synthesis of glycopolymer coated nanoparticles. Polymerization of *N*-hydroxy ethyl acrylamide (HEA) by RAFT, followed by displacement of the PFP ester with galactosamine, and finally assembly of the glycopolymers onto AuNP size 16 nm.

**Table 1:** Polymers prepared for detection of SBA

Polymer	[M] : [CTA]^a^	*M*_n (theo)_ b [g mol^−1^]	*M*_n (SEC)_ c [g mol^−1^]	DP_(NMR)_ d	*Đ*_M_ c
PHEA_40_	15 : 1	2260	4900	16	1.14
PHEA_72_	35 : 1	4560	8700	39	1.11

a Molar ratio of monomer (M) to chain transfer agent (CTA).

b Theoretical *M*_n_ considering molar ratio of chain transfer agent (CTA) to monomer (M).

c *M*_n_ and *Đ*_M_ determined by SEC in DMF eluant containing 5 mM NH_4_BF_4_, calibrated with poly(methyl methacrylate) standards at 50 °C at flow rate of 1.0 mL min^−1^.

d Degree of polymerisation (DP) determined from ^1^H NMR end-group analysis. Polymers are referred to by their DP SEC in this paper.

Citrate stabilised 16 nm AuNPs were synthesised (method found in SI) by a seeded growth approach and characterised by DLS and UV-Vis (SI Fig. S2, S5, and Table S2), they were then functionalised with glycosylated polymers following published procedures to produce Gal-PHEA_40_@AuNP_16_ and Gal-PHEA_72_@AuNP_16_ (Fig. 1).^28^ Functionalisation of the nanoparticles by the polymer was confirmed by UV-Vis as shown by a shift in the UV_max_, further characterisation was also carried out by DLS (SI Fig. S3– S5, and Table S2).

All discussed above methods can be found in the SI. With the glycopolymer functionalised nanoparticles to hand, Gal-PHEA_72_@AuNP_16_ and Gal-PHEA_40_@AuNP_16_, these were assessed against differing concentrations of biological media and lectin concentration. Each system was tested in triplicate using the lectin soybean agglutinin (SBA) as the target protein, a lectin with known selectivity for galactose derivatives, and bovine serum albumin (BSA) as an off-target control protein that when conjugated with glycans is often used as a test line in LFGAs.

Artificial saliva, artificial urine, and defibrinated sheep's blood were employed as the biologically relevant media to assess the robustness of our glycoassays. Artificial human saliva is comparable to human saliva in terms of viscosity, density, and pH.^44^ Synthetic urine is near indistinguishable from real urine, unless specific tests are used to distinguish synthetic *versus* real, for example considering long-duration biomolecules inherent in human urine, and short-duration biomolecules that degrade quickly. These markers go beyond the scope of this paper and were not targeted.^45^ Ovine blood (defibrinated sheep's blood), has also been demonstrated to be a suitable alternative for human blood in a multitude of cases including thrombogenicity testing,^46^ which is essential for blood-contacting medical devices such as stents or artificial organs, it is therefore a reasonable alternative for human blood when considering the robustness of our glycoassays. For clarification, defibrinated sheep blood was purchased and used without further purification or pre-treatment. Defibrinated sheep cells are aseptically collected whole sheep blood samples that had been processed to remove fibrin. There are no additives or preservatives in this product. Notably, buffer conditions were kept the same across all tests. While optimisation of the buffer could be undertaken this is beyond the scope of this work.

Flow-through devices (SI Fig. S6) were developed first using target analyte (SBA) concentrations from 0.1 mg mL^−1^ to 0.5 mg mL^−1^ to determine on-target binding, and an off-target control analyte (BSA) concentration of 0.5 mg mL^−1^ with no anticipated affinity to the nanoparticles, to determine if there was any off-target binding. The SBA concentration range of 0.1 mg mL^−1^ to 0.5 mg mL^−1^ was chosen as previous research determined the limit of detection (LoD) in a lateral flow format to be 0.02 mg mL^−1^ in a buffered solution.^28^ Based on the previous works conclusion, this work focuses on investigating the influence of biological media rather than re-iterating the concentration response behaviour of the assay. Therefore, a concentration range of 0.1 mg mL^−1^ to 0.5 mg mL^−1^ was explored for SBA in flow-through, and 0.1 mg mL^−1^ in lateral flow.

In a flow-through device no antibody or glycan test line is used, instead 1 µL of analyte (SBA or BSA) was deposited onto the dipstick in place of the test line. The differing concentrations of lectin were tested *versus* differing concentrations of biologically relevant media (0%–65%) doped into buffer which also contained one of the AuNP systems Gal-PHEA_40_@AuNP_16_ or Gal-PHEA_72_@AuNP_16_, Fig. 2A & B (SI Tables S3 and S4).

**Fig. 2:**
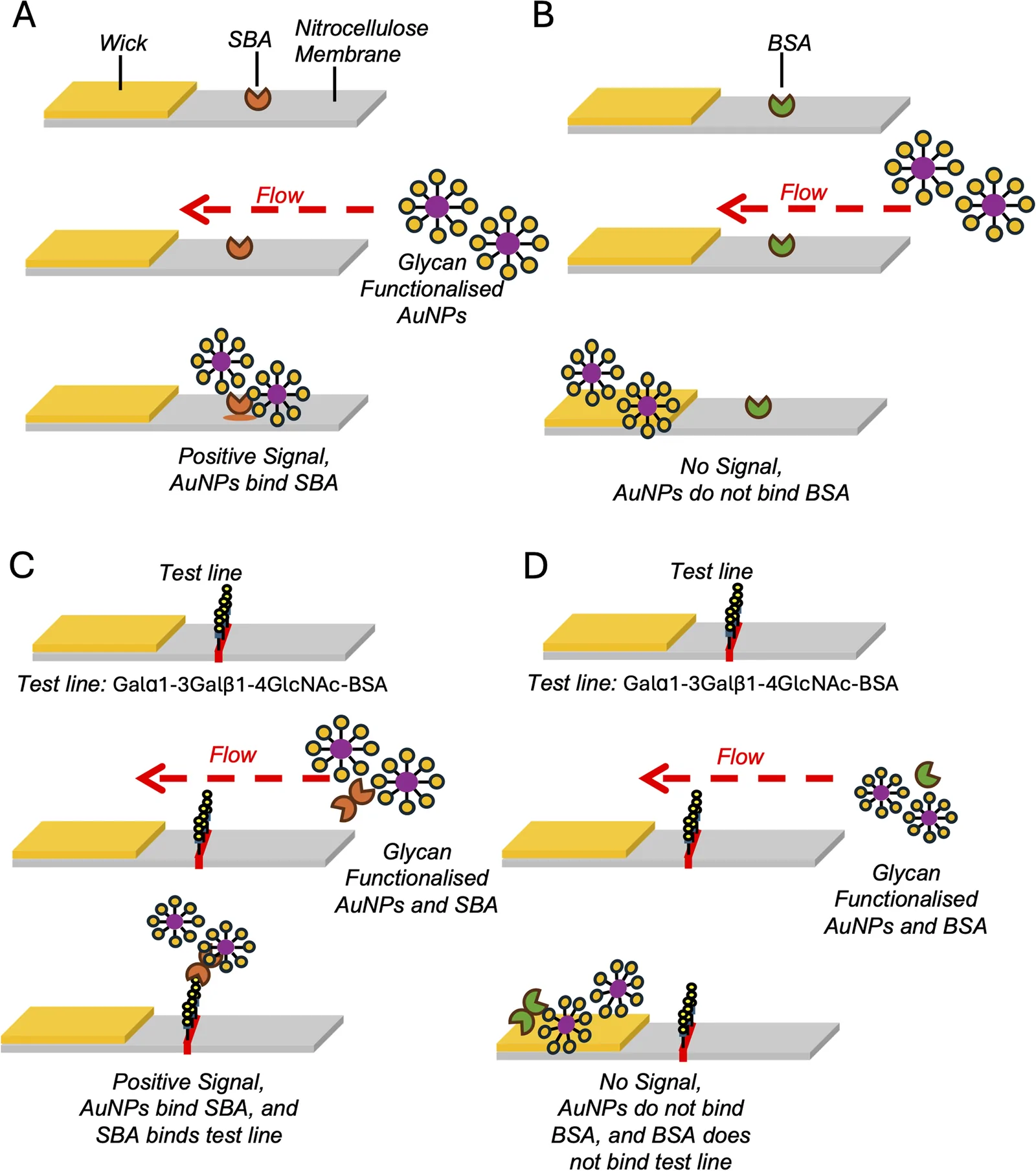
Schematic of dipstick flow-through and lateral flow assay. (A) Flow-through assay with SBA test line and glycan functionalised AuNPs, showing a positive signal from binding. (B) Flow-through assay with BSA test line and glycan functionalised AuNPs, showing no binding *i.e.* no signal. (C) Lateral flow assay with Galα1-3Galβ1-4GlcNAc-BSA test line and SBA (analyte), showing a positive signal from binding. (D) Lateral flow assay with Galα1-3Galβ1-4GlcNAc-BSA test line and BSA (negative control), showing no binding *i.e.* no signal.

Examples of positive (SBA as analyte) and negative (BSA as analyte) flow-through dipsticks are shown in Fig. 3. Note, the wick area, which is often hidden by a cassette, is exposed in Fig. 3. At the end of the assay, unbound particles gather at the wick. Full scans and analysis of all strips can be found in the SI alongside a representative figure of a processed grey value plot and representative dipstick (SI Fig. S7). The signal to noise ratio (SNR) was determined for each system by measuring the maximum intensity of the test line *versus* the average intensity around the test line. The SNRs for the BSA control were then subtracted from the SBA (test) SNRs to give Fig. 4.

**Fig. 3:**
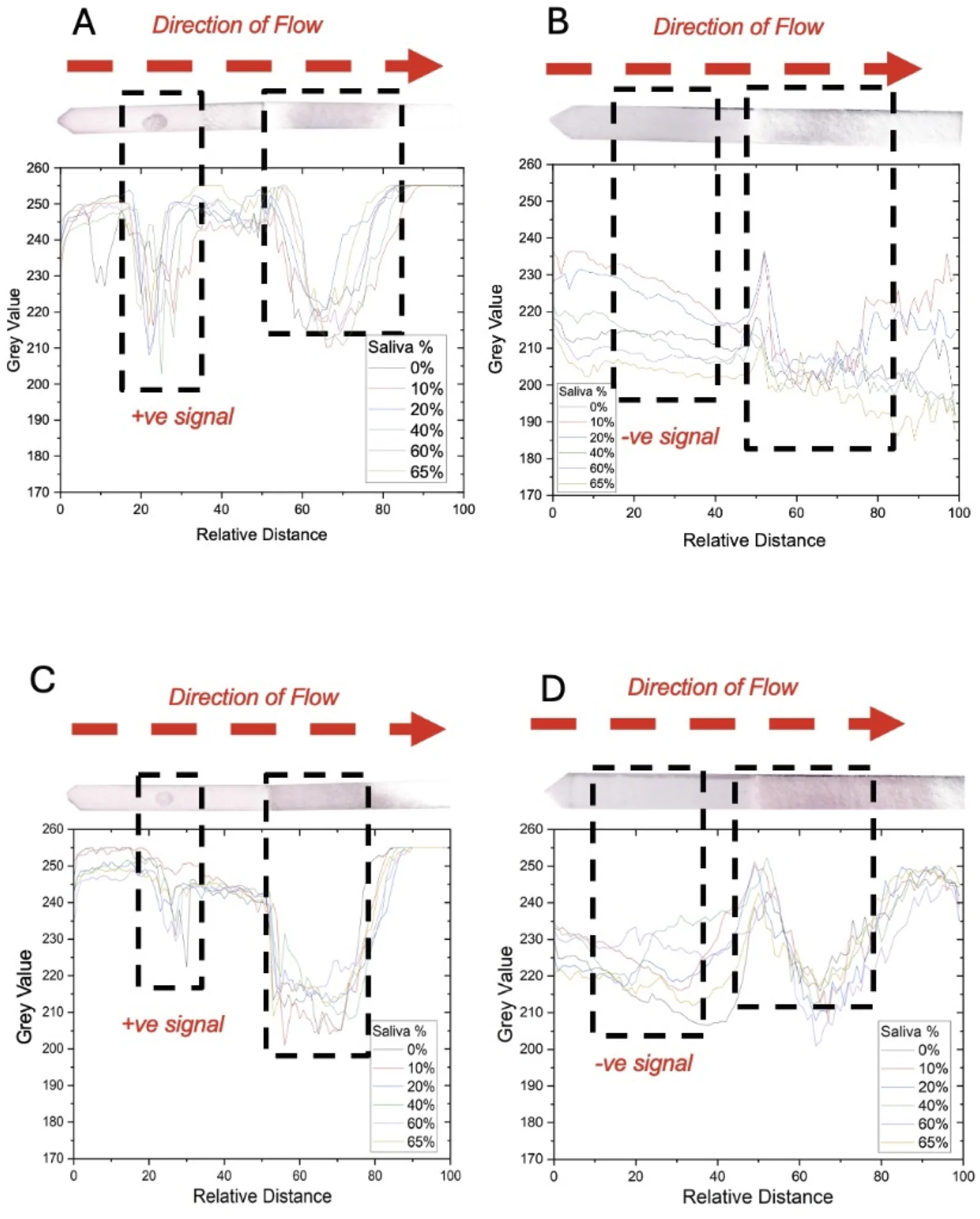
Example flow-through strips and analysis with saliva doped buffer at varying percentages, and analyte concentrations of 0.5 mg mL^−1^. (A) Gal-PHEA_40_@AuNP_16_*versus* SBA, (B) Gal-PHEA_40_@AuNP_16_*versus* BSA, (C) Gal-PHEA_72_@AuNP_16_*versus* SBA, and (D) Gal-PHEA_72_@AuNP_16_*versus* BSA.

**Fig. 4:**
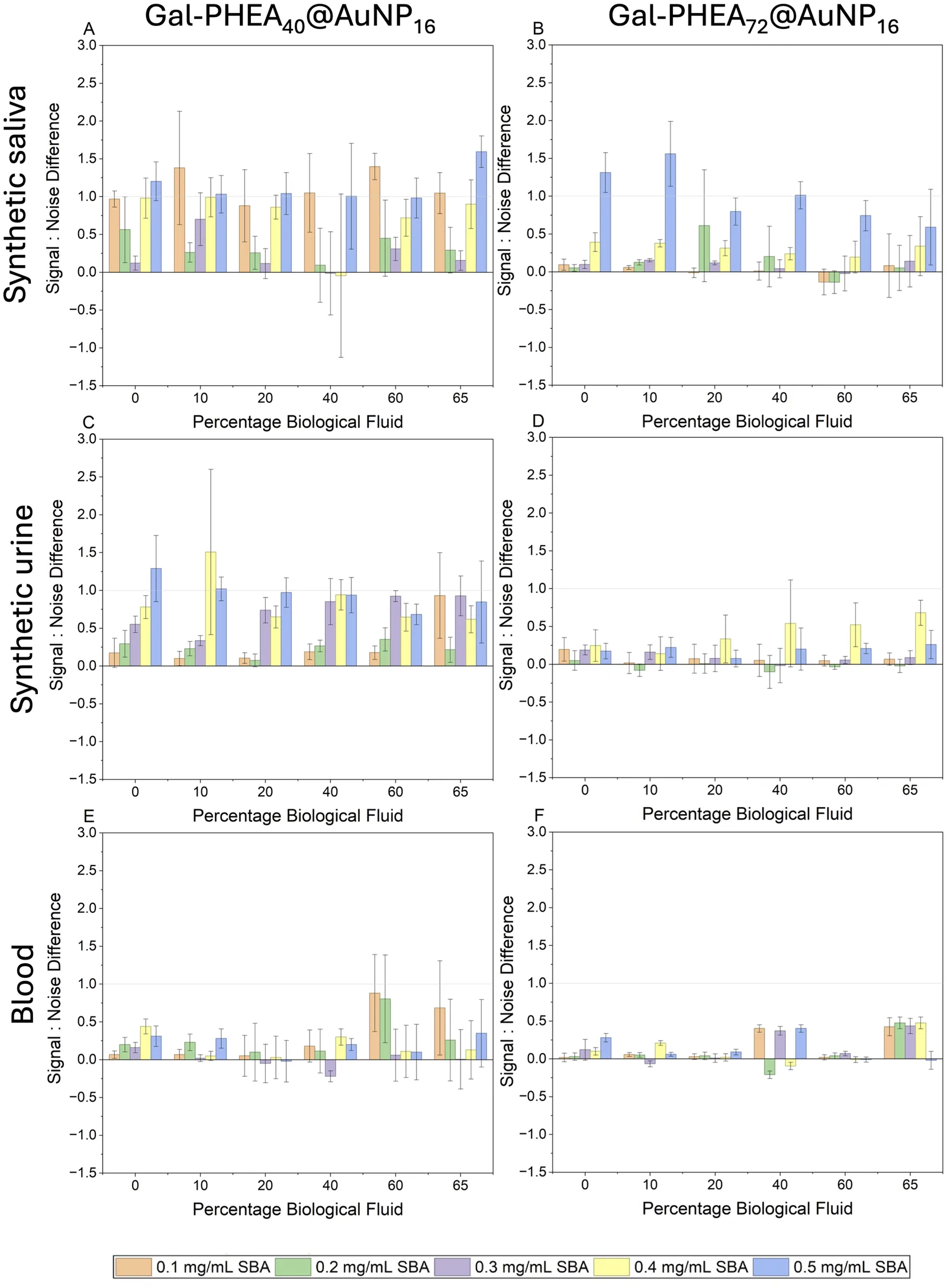
Results from flow-through testing. Signal to noise analysis (SBA SNR – BSA SNR) of doped buffers at varying percentages of media, and varying SBA concentrations of 0.1 mg mL^−1^ to 0.5 mg mL^−1^. (A) Gal-PHEA_40_@AuNP_16_ in saliva, (B) Gal-PHEA_72_@AuNP_16_ in saliva, (C) Gal-PHEA_40_@AuNP_16_ in urine, (D) Gal-PHEA_72_@AuNP_16_ in urine, (E) Gal-PHEA_40_@AuNP_16_ in blood, and (F) Gal-PHEA_72_@AuNP_16_ in blood.

In terms of flow-through (SI Tables S5–S14, and Fig. S8–S32), consideration of the data revealed the following trends. Saliva, in comparison to blood and urine, provided the highest SNR differences overall, with 0.5 mg mL^−1^ heavily outcompeting other concentrations for the highest media concentrations for Gal-PHEA_40_@AuNP_16_ and all for Gal-PHEA_72_@AuNP_16_. Overall in saliva samples, decreasing the concentration of the lectin showed a decrease in the SNR difference. In comparison to Gal-PHEA_72_@AuNP_16_, Gal-PHEA_40_@AuNP_16_ across multiple media concentrations had higher SNR differences even with decreasing lectin concentration – showing a trend favouring higher SNR differences with Gal-PHEA_40_@AuNP_16_ compared to Gal-PHEA_72_@AuNP_16_. Consideration of the error bars (1 standard deviation) across the conditions however suggests that the difference is not statistically robust at all concentrations. However, it should be noted that a visible trend is seen for the middle concentrations giving on average a lower SNR difference compared to the lower and higher concentrations. Since this trend was not observed in either blood or urine, it could possibly be due to other biological components in the saliva that affect the assay in ways that are competing or interfering with SBA binding.

For synthetic urine, the Gal-PHEA_72_@AuNP_16_ signal exhibited less variation but lower SNR differences across (i) differing matrix concentrations and (ii) differing lectin concentrations for each matrix concentration *versus* Gal-PHEA_40_@AuNP_16_, which is demonstrated by the smaller error bars. While Gal-PHEA_40_@AuNP_16_ showed greater susceptibility to changes in media concentration *versus* Gal-PHEA_72_@AuNP_16_ as SBA concentration changed. The highest SNR difference recorded for Gal-PHEA_40_@AuNP_16_ was with conditions of 10% biological media and 0.4 mg mL^−1^ lectin concentration, whereas in comparison, for Gal-PHEA_72_@AuNP_16_, 0.4 mg mL^−1^ was shown to be most effective in 65% biological media concentration, demonstrating the impact of polymer length. Overall, Gal-PHEA_40_@AuNP_16_ is shown to be a more optimal length when compared to Gal-PHEA_72_@AuNP_16_ as it provides on average higher SNR differences, especially in 10% and 40% doped media where it is evident that changing lectin concentration affects SNR output.

For blood, it was evident that across differing media concentrations, once again Gal-PHEA_72_@AuNP_16_ provided a lower SNR difference, with substantial change only being seen at opposite ends of media concentration, that being 0% and 65%. Though overall for blood, SNR differences for both polymer lengths remained low (never breaching the threshold of 1.5) in comparison to urine and saliva. This is attributed to the complexity of blood and the tendency for aggregation which leads to problems in flowing ‘through’ the dipstick to the test line. However, once again, for most media concentrations the optimal particle was Gal-PHEA_40_@AuNP_16_.

The standard deviation error bars across all conditions supports the conclusion that the longer polymer system, Gal-PHEA_72_@AuNP_16_, provides greater reproducibility across all conditions, particularly for the urine and blood matrices. However, this also comes at a cost of visibly lower SNR differences on average. Therefore, there is a significant trade-off between stability and sensitivity that one must consider in selecting appropriate polymer lengths for a given application. Large error bars, mainly observed in saliva for Gal-PHEA_40_@AuNP_16_, provides insight into the strip-to-strip variability within a triplicate group. It should also be noted that negative SNR differences do not equate to binding of the control (BSA). Those with negative SNR differences have no visible test line spot on either the SBA or equivalent BSA test strips, however strip surface variations can interfere with SNR calculations through (a) inconsistent flow or (b) buildup of fluid/nanoparticles which can in turn depress the SBA signal, bringing the SNR below the equivalent BSA strip SNR. As well, across conditions in both Gal-PHEA_40_@AuNP_16_ and Gal-PHEA_72_@AuNP_16_, there is some overlap in standard deviation error bars which indicates that differences between specific conditions cannot be considered statistically significant at *n* = 3 (triplicate testing), though general trends are still observed.

In all, for each media group, increasing lectin concentration increased SNR difference. This highlights the benefits of this tuneable system, wherein changing lectin concentration and/or polymer length can provide vastly different results allowing for user control depending on system specifics, Fig. 4.

After the particle systems had been interrogated in flow-through, it was decided to test the system in a full lateral flow glycoassay at the lowest concentration of analyte (0.1 mg mL^−1^ analyte) doped into the buffer, in order to truly challenge the robustness of the assay, Fig. 2C & D (SI Tables S15, S16, and Fig. S33–S41). In the lateral flow glycoassay setup, a test line of 1 µL of a 0.5 mg mL^−1^ of Galα1-3Galβ1-4GlcNAc-BSA was used. This BSA glycoconjugate is known to have affinity for SBA and has previously been used by Baker *et al.* for this purpose.^28^Fig. 5 which represents the SNR differences of the lateral flow results includes total error which was calculated by adding the SBA SNR error and BSA SNR error together. There is again some overlap in the standard deviation error bars, similar to flow-through, which indicates that differences between specific conditions cannot be considered statistically significant at *n* = 3 (triplicate testing), though the following general trends are still observed.

**Fig. 5:**
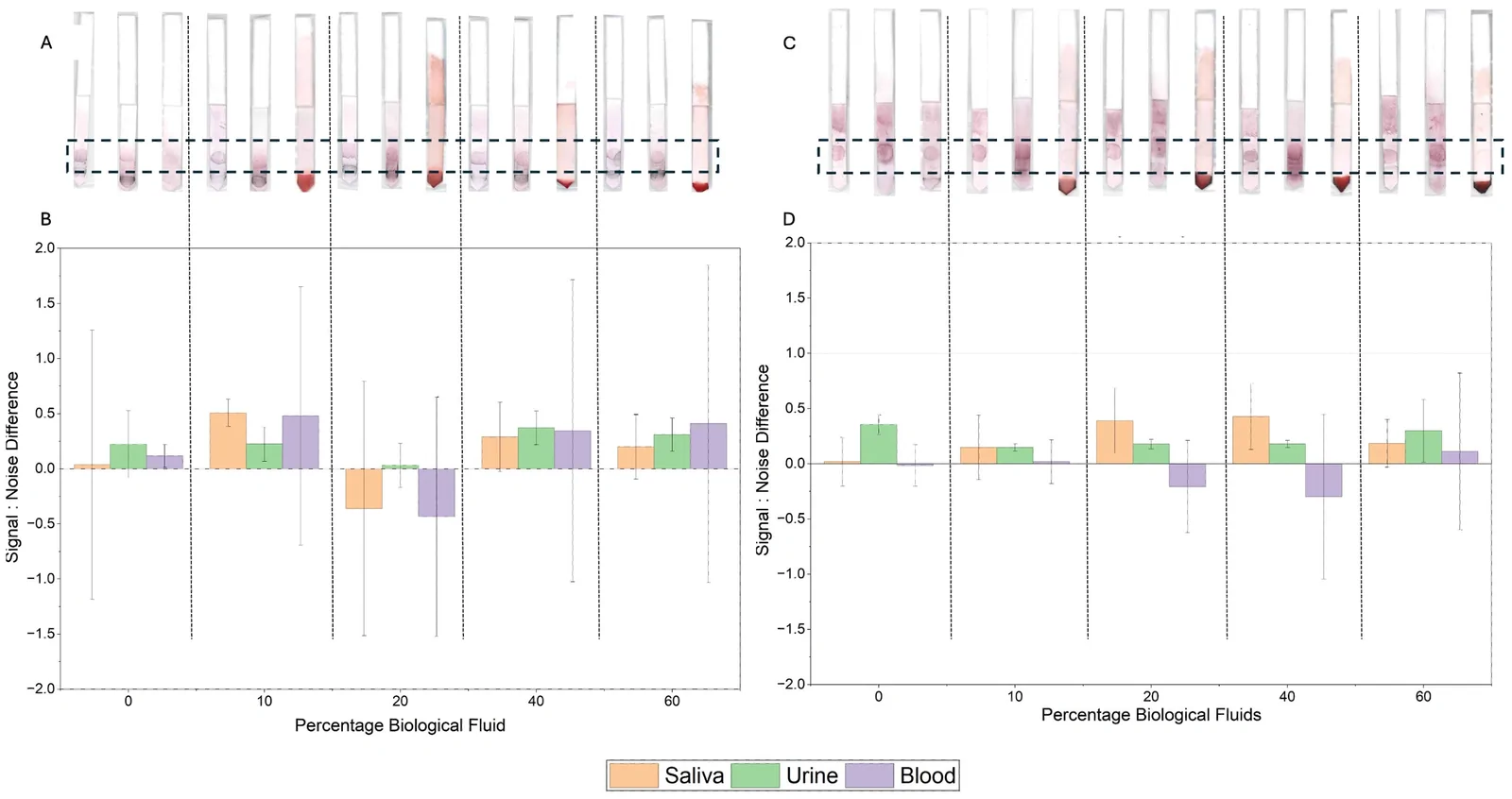
Results from lateral flow testing. Signal to noise analysis, SBA concentrations of 0.1 mg mL^−1^ in doped buffers (varying concentrations of dopant saliva, urine and blood), with a 0.5 mg mL^−1^ test line of Galα1-3Galβ1-4GlcNAc-BSA. (A) Representative strip images of Gal-PHEA_40_@AuNP_16_ results, (B) Gal-PHEA_40_@AuNP_16_ results, (C) representative strip images of Gal-PHEA_72_@AuNP_16_ results, (D) Gal-PHEA_72_@AuNP_16_ results. Position of the test line in strip images is indicated by the black dash box.

Gal-PHEA_40_@AuNP_16_ was again more susceptible to changes in (i) media type and (ii) media concentration. This is shown by saliva and urine, in Gal-PHEA_40_@AuNP_16_, whose SNR differences changed significantly depending on the media percentages. LFGAs in blood produced higher SNR differences on average with the shorter length Gal-PHEA_40_@AuNP_16_, whereas urine and saliva producing higher SNR differences with the longer length polymer. This is most notable for 60% blood between Gal-PHEA_40_@AuNP_16_ and Gal-PHEA_72_@AuNP_16_, with the two systems showing differing results illustrating the importance of tuning the polymer system depending on media type and media concentration. For example, changing the polymer length from Gal-PHEA_40_@AuNP_16_ to Gal-PHEA_72_@AuNP_16_ provided lower SNR differences in 40% blood, but this change also affected the SNR difference produced for saliva, which gave an improvement in signal difference. For saliva and urine, Gal-PHEA_40_@AuNP_16_ was seen to be the more optimal polymer length for lower concentrations of media as it provided higher SNRs differences on average regardless of changing lectin concentration when compared to Gal-PHEA_72_@AuNP_16_. However, for blood, Gal-PHEA_40_@AuNP_16_ provides a higher SNR difference than the shorter polymer length, Fig. 5. This is different to the flow-through systems where the shorter polymer was more optimal across all the different biological media.

Considering the outcomes from the flow-through and lateral flow systems, this work displays how each system can be tuned with lectin concentration, media concentration, and polymer length to enable the modulation of observable outputs, allowing for user specific systems to be made. Tunability of polymer length especially, shows how in comparison to antibodies, LFGAs can be modified to output optimal results depending on the media being used such as urine, blood, or saliva.

## Conclusions

Lateral flow glycoassays (LFGAs) have been shown to be a viable option for rapid diagnostic testing,^25–28^ this is further supported by this work that provides a better understanding of their capabilities regarding testing with biologically relevant media, that has not previously been undertaken to the best of our knowledge. Crucial observations were made on how lectin concentration and polymer length changed the performance of the different assays (flow-through *versus* lateral flow) in biologically relevant media. Gal-PHEA_72_@AuNP_16_, in comparison to Gal-PHEA_40_@AuNP_16_, seemed to enhance stability and reproducibility whereas the shorter chain provided greater signal to noise variation in less complex media such as saliva and urine. The trends in saliva and urine in general gave higher SNR differences at lower media concentrations, inferring that increasing concentration interferes with lectin interactions and flow performance. Blood, in all testing conditions, routinely gave lower SNR differences due to issues with its viscosity and complexity, thus highlighting the need for further testing or another preparation.

In summary, the variation of SNR differences observed depended on (i) the biologically relevant media, (ii) lectin concentration used, and (iii) the polymer length utilised. The importance of polymer chain length is notable as it shows how the assay can be easily tuned for accuracy depending on the environment being tested, a benefit of polymer-based LFGAs compared to their antibody-based counterparts. Further optimisation could be undertaken for example by; changing the monomer, testing further variations in chain length, changing the diameter of the nanoparticle, utilising anisotropic gold nanoparticles or quantum dots, and varying the buffer components – although this is beyond the scope of this work. While the LFGAs presented in this paper, and by previous work of the group, have demonstrated many advantages such as synthetic flexibility, further studies should be undertaken to evaluate their full potential.

This paper, therefore, presents a novel study of polymer-based LFGAs in biological media and has demonstrated their robustness to the point that these devices have the capability to be applied in a broad manner, through polymer tunability, for diagnostic testing.

## Conflicts of interest

There are no conflicts to declare.

## Supplementary Material

LP-OLF-D6LP00336B-s001

## Data Availability

The data supporting this article have been included as part of the supplementary information (SI). Supplementary information: full experimental details; characterisation; flow-through strip images and analysis; and lateral flow strip images and analysis. See DOI: https://doi.org/10.1039/d6lp00336b.
