# Supplementary material for: The robustness of polymer-based lateral flow glycoassays in medically relevant media
Source: RSC Appl Polym. 2026 Oct 22. Online ahead of print. doi: 10.1039/d6lp00336b (PMC13688169; doi:10.1039/d6lp00336b)
Supplement: Supplementary file 1 [file LP-OLF-D6LP00336B-s001.pdf]

Supplementary Information (SI) for RSC Applied Polymers.
This journal is © The Royal Society of Chemistry

Supplementary Information

The Robustness of Polymer-Based Lateral Flow GlycoAssays in Medically Relevant Media

*Megan Ellis,[a‡] Zaimire Maimaitiaili,[a‡] Douglas E. Soutar,[a] Alexander J. Cantor,[a] Jack C. Oram,[a] Anish Mistry,[a] Garrett D. Jackson,[a] and Alexander N. Baker[a]**

[a] Department of Chemistry, University of Warwick, Gibbet Hill Road, CV4 7AL, Coventry, UK

* *Corresponding Author*: alexander.baker@warwick.ac.uk

Contents

Physical and Analytical Methods *5*

NMR Spectroscopy 5

Centrifugation 5

Size Exclusion Chromatography 5

Dynamic Light Scattering 6

UV-Vis Spectroscopy 6

Image Collection of Lateral Flow Dipsticks and Devices 6

Materials *8*

Synthetic Methods *10*

Polymerisation of 2-Hydroxyethyl Acrylamide 10

Representative DP40 Poly(N-hydroxyethyl acrylamide) glycan functionalisation 11

Representative Citrate-Stabilised 16nm Gold Nanoparticle Synthesis 12

Gold Nanoparticle Coating Functionalisation 12

Size Exclusion Chromatography *14*

Dynamic Light Scattering Data *15*

UV-Vis Data *18*

Lateral Flow Strip Production, Running and Analysis Protocols *19*

Protocol for Manufacturing Lateral Flow Strips 19

Assay Buffer – 10x HEPES buffer (10% PVP400) in 100 mL H_2O 20

Protocol for Running Flow-Through Test 20

Protocol for Test Line Addition for Lateral Flow Test 21

Protocol for Running Lateral Flow Test 21

Standard Protocol for Lateral Flow and Flow Through Strip Analysis 22

Lateral Flow Signal to Noise Analysis Relative Distance Pixel 23

Flow-Through Test Results *25*

Signal to Noise Results for Flow-Through *49*

Lateral Flow Test Results *63*

Signal to Noise Results for Lateral Flow *69*

References *75*

Physical and Analytical Methods

NMR Spectroscopy

^{1}H NMR, ^{13}C{1H} NMR and ^{19}F NMR spectra were recorded at 300 MHz on a Bruker Avance III HD 300 MHz or Bruker Avance III HD 400 MHz spectrometer respectively, with deuterium oxide (D_2O) as the solvent at 298K. Chemical shifts of protons are reported as δ in parts per million (ppm) and are relative to D_2O (4.79 ppm).

Centrifugation

Centrifugation was performed on Thermo Scientific Multifuge X1 Centrifuge and Thermo Scientific mySPIN12. Citrate-Stabilised 16 nm gold nanoparticles and DP40/72 Poly(N-hydroxyethyl acrylamide) glycan functionalisation were carried out at 12,500 RPM (RCF 18,151 xg). 2-Hydroxyethyl Acrylamide was centrifuged at 5000 RPM (RCF 10,000 xg).

Size Exclusion Chromatography

SEC analysis was performed on an Agilent Infinity II MDS instrument equipped with differential refractive index (DRI), viscometry (VS), dual angle light scatter (LS) and variable wavelength UV detectors. The system was equipped with 2 x PLgel Mixed D columns (300 x 7.5 mm) and a PLgel 5 μm guard column. The mobile phase used was *N,N*-dimethylformamide (DMF) (HPLC grade) containing 5 mM NH_4BF_4 at 50 °C with a flow rate of 1.0 mL/min. Poly(methyl methacrylate) (PMMA) standards (Agilent EasyVials) were used for calibration between 955,000 – 550 g.mol^{-1}.

Analyte samples were filtered through a nylon membrane with 0.22 μm pore size before injection. Number average molecular weights (M_n), weight average molecular weights (M_w) and dispersity ($Đ = M_w/M_n$) was determined by calibration using RI against PMMA standards using Agilent GPC/SEC software.

Dynamic Light Scattering

Hydrodynamic diameters (D_h) and size distributions of particles were determined by dynamic light scattering (DLS) using a Malvern Zetasizer Nano ZS with a 4 mW He-Ne 633 nm laser module operating at 25 °C. Measurements were carried out at an angle of 173° (back scattering), and results were analysed using Malvern DTS 7.03 software. All determinations were repeated 5 times with at least 10 measurements recorded for each run. D_h values were calculated using the Stokes- Einstein equation where particles are assumed to be spherical.

UV-Vis Spectroscopy

Absorbance measurements were recorded on an Agilent Cary 60 UV-Vis Spectrophotometer and on a BioTek Epoch microplate reader.

Image Collection of Lateral Flow Dipsticks and Devices

All devices were scanned using a Canon IJ Scan Utility Lite to a pdf file that was converted to a jpeg. The jpeg was analysed in ImageJ 1.51. None of the images in this ESI have been image adjusted i.e. no changes/enhancements have been made from the original scan images.

Materials

N-Hydroxyethyl acrylamide (97%), 4,4'-azobis(4-cyanovaleric acid) (ACVA, 98%), 4-dimethylaminopyridine (>98%), triethylamine (>99%), sodium citrate tribasic dihydrate (>99%), gold(III) chloride trihydrate (99.9%), deuterium oxide (D_2O, 99.9%), deuterated chloroform ($CDCl_3$, 99.8%), diethyl ether (≥99.8%, ACS reagent grade), methanol (≥99.8%, ACS reagent grade), toluene (≥99.7%,), Tween-20 (molecular biology grade), HEPES, PVP40 (poly(vinyl pyrrolidone with average Mw ≈ 40 000), sucrose (Bioultra grade), carbon disulfide (≥99.8%), acetone (≥99%), 1-dodecane thiol (≥98%), N-pentylamine (99%), and pentafluorophenol (≥ 99%, reagent plus) were purchased from Sigma-Aldrich. Anhydrous trehalose was purchased from Alfa Aesar. DMF (>99%) and 2-bromo-2-methyl propionic acid (98%) were purchased from Acros Organics. D-(+)-galactosamine·HCl and 1-ethyl-3-(3- dimethylaminopropyl)carbodiimide hydrochloride (EDC) (>98%), were purchased from Carbosynth. Sodium chloride (≥99.5%), calcium chloride, hydrochloric acid (≈37%, analytical grade), glacial acetic acid (analytical grade), and magnesium sulfate (reagent grade) were purchased from Thermo Fisher Scientific. Nitrocellulose Immunopore RP 90-150 s/4cm 25mm was purchased from GE Healthcare. Lateral flow backing cards 60mm by 301.58mm (KN-PS1060.45 with KN211 adhesive) was purchased from Kenosha Tapes. Cellulose fibre wick material 20 cm by 30 cm by 0.825 mm (290 gsm and 180 mL/min) (Surewick CFSP223000) was purchased from EMD Millipore. Unfunctionalised BSA was purchased from Merck. Thick Chromatography Paper (for sample pads), Grade 237, Ahlstrom 20 cm by 20 cm was purchased from VWR International. Lectin from glycine max (soybean) was purchased from Merck. Artificial saliva for Pharmaceutical Research and Sigmatrix Urine Diluent was purchased from

Merck. Defibrinated Sheep Blood was purchased from E&O Laboratories and used without further purification or pre-treatment. Defibrinated sheep cells are aseptically collected whole sheep blood samples that had been processed to remove fibrin. There are no additives or preservatives in this product. Ultra-pure water used for buffers was Elga grade 15 MΩ resistance.

Synthetic Methods

Polymerisation of 2-Hydroxyethyl Acrylamide

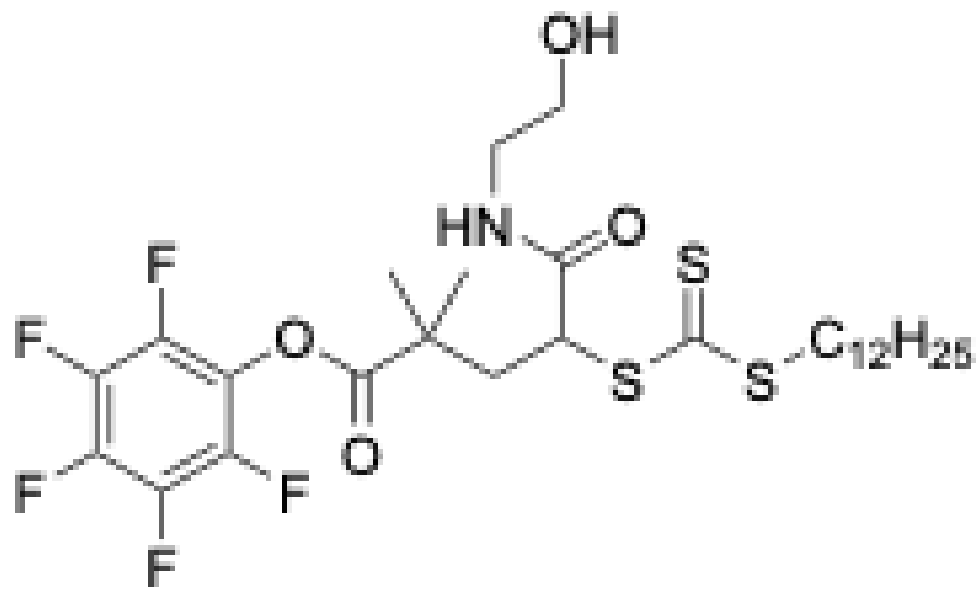

This was synthesised according to a previously published procedure.[1] $PHEA_{40}$ was used as a representative example, [M]:[CTA] of 15:1.

0.51 g (4.402 mmol) of 2-hydroxyethyl acrylamide, 0.0165 g (0.110 mmol) of ACVA, and 0.13 g (0.240 mmol) of PFP-DMP was added to 4 mL 1:1 toluene : methanol. The reaction vessel was degassed by sparging with nitrogen for 20 min then stirred and heated to 70 °C for 2 h. The reaction was halted by cooling with liquid nitrogen, and a gel formed. The crude product was dissolved in the minimum amount of methanol. 25 mL of cold diethyl ether was added to the methanol to form a precipitate. The mixture was centrifuged for 3 min at 5000 RPM (RCF 10,000 xg) and the liquid decanted off. The solid was dissolved in 7 mL methanol and diethyl ether was added to be centrifuged for 3 min at 5000 rpm. The product was dried under vacuum to give a yellow crystalline solid, structure was confirmed by ^{1}H-NMR.

The same method was used for the following [M:CTA] ratio with the following amounts. For [M:CTA] 32:1, 1 g (8.67 mmol) of 2-hydroxyethyl acrylamide, 0.015g (0.057 mmol) of ACVA, and 0.143 g (0.270 mmol) of PFP-DMP for the synthesis of $PHEA_{72}$.

$PHEA_{40}$ – δH (300 MHz, D_2O ; M) 7.67 - 8.95 (5H, NH), 3.52 - 4.08 (32H, NHC**H**$_2$), 2.87 - 3.43 (34H, C**H**$_2$OH), 1.15 - 2.69 (54H, **CH**$_2$**CH** & $(CH_3)_2$), 0.90 - 1.13 (20H, (C**H**$_2$)10), 0.49 - 0.74 (3H, (CH_2)10C**H**$_3$).

$PHEA_{72}$ – δH (300 MHz, D_2O; M) 3.52 - 4.04 (78H, NHC**H**$_2$), 2.95 - 3.43 (85H, C**H**$_2$OH & diethyl ether contaminant), 1.18 - 2.42 (120H, CH_2CH & $(CH_3)_2$), 1.00 - 1.17 (31H, (CH_2)10 & diethyl ether contaminant), 0.56 - 0.78 (3H, CH_3).

Representative DP40 Poly(N-hydroxyethyl acrylamide) glycan functionalisation

This was synthesised according to a previously published procedure.[1] 0.21 g (0.04 mmol) of $PHEA_{40}$ and 0.020 g (0.004 mmol) of D-(+)- galactosamine. HCl were added to 25 mL of DMF containing 100 μL TEA. The reaction was stirred at 50 °C for 16 h. Solvent was removed under vacuum. The crude product was dissolved in the minimum amount of methanol at RTP before cooling in a liquid nitrogen bath. Diethyl ether cooled in liquid nitrogen was added to the methanol to form a precipitate. The mixture was centrifuged for 2 min at 12,500 RPM (RCF 18,151 xg) and the liquid decanted off. The solid was dissolved in methanol and removed under vacuum to give an orange/brown solid. Structure was confirmed by ^{1}H-NMR.

$PHEA_{40}$ Glycan Functionalization – δ_H (300 MHz, D_2O) 8.04 – 7.94 (6H, m, NH), 4.99 – 4.88 (2H, anomeric protons), 4.13 – 3.45 (~90H, m, $NHCH_2$ & glycan protons), 3.45 – 3.0 (~80H, m, CH_2OH & SCH_2 & glycan protons), 2.46 – 1.82 (~50H, m, $CH_2CHC(O)$, $C(CH_3)_2$ & glycan protons), 1.82 – 1.30 (98H, m, $CH_2CHC(O)$ & $(CH_2)_9CH_3$), 0.89 – 0.66 (3H, m, CH_3).

$PHEA_{72}$ Glycan Functionalization – δ_H (300 MHz, D_2O) 8.04 – 7.87 (6H, m, NH), 4.90 – 4.88 (2H, anomeric protons), 4.13 – 3.45 (~90H, m, $NHCH_2$ & glycan protons), 3.45 – 3.0 (~80H, m, CH_2OH & SCH_2 & glycan protons), 2.48 – 1.80 (~50H, m, $CH_2CHC(O)$, $C(CH_3)_2$ & glycan protons), 1.80 – 1.28 (98H, m, $CH_2CHC(O)$ & $(CH_2)_9CH_3$), 0.83 – 0.69 (3H, m, CH_3).

Representative Citrate-Stabilised 16nm Gold Nanoparticle Synthesis

This was synthesised according to a previously published procedure.[2] To 100 mL of water, 0.032 g (0.082 mmol) of gold(III) chloride trihydrate was added. The mixture was heated to reflux and 14.6 mL of water containing 0.09 g (0.292 mmol) of sodium citrate tribasic dihydrate was added when the reaction temperature came to 100 °C. The reaction was allowed to reflux for 30 minutes before cooling to room temperature over 3 hours. The solution was centrifuged at 12,500 RPM (RCF 18,151 xg) for 30 minutes and the pellet resuspended in 40 mL of water to give an absorbance at 520 nm of ~1 Abs.[3]

Gold Nanoparticle Coating Functionalisation

This was synthesised according to a previously published procedure.[2,3] Stock solutions of each polymer (Gal-PHEA$_{40}$, Gal-PHEA$_{72}$) were prepared by dissolving 10 mg of each separately into 1 mL of distilled water. 100 μL of each stock solution was added individually to 1 mL of OD 0.55 gold nanoparticles of the desired size. The nanoparticle solutions were left to functionalise overnight with agitation on a roller. The nanoparticle solutions were then centrifuged for 30 minutes at the speeds corresponding to their size, the supernatant removed, and the solutions re-diluted with distilled water. Two further washes were performed with 15- minute centrifugations at the corresponding speeds.

Size Exclusion Chromatography

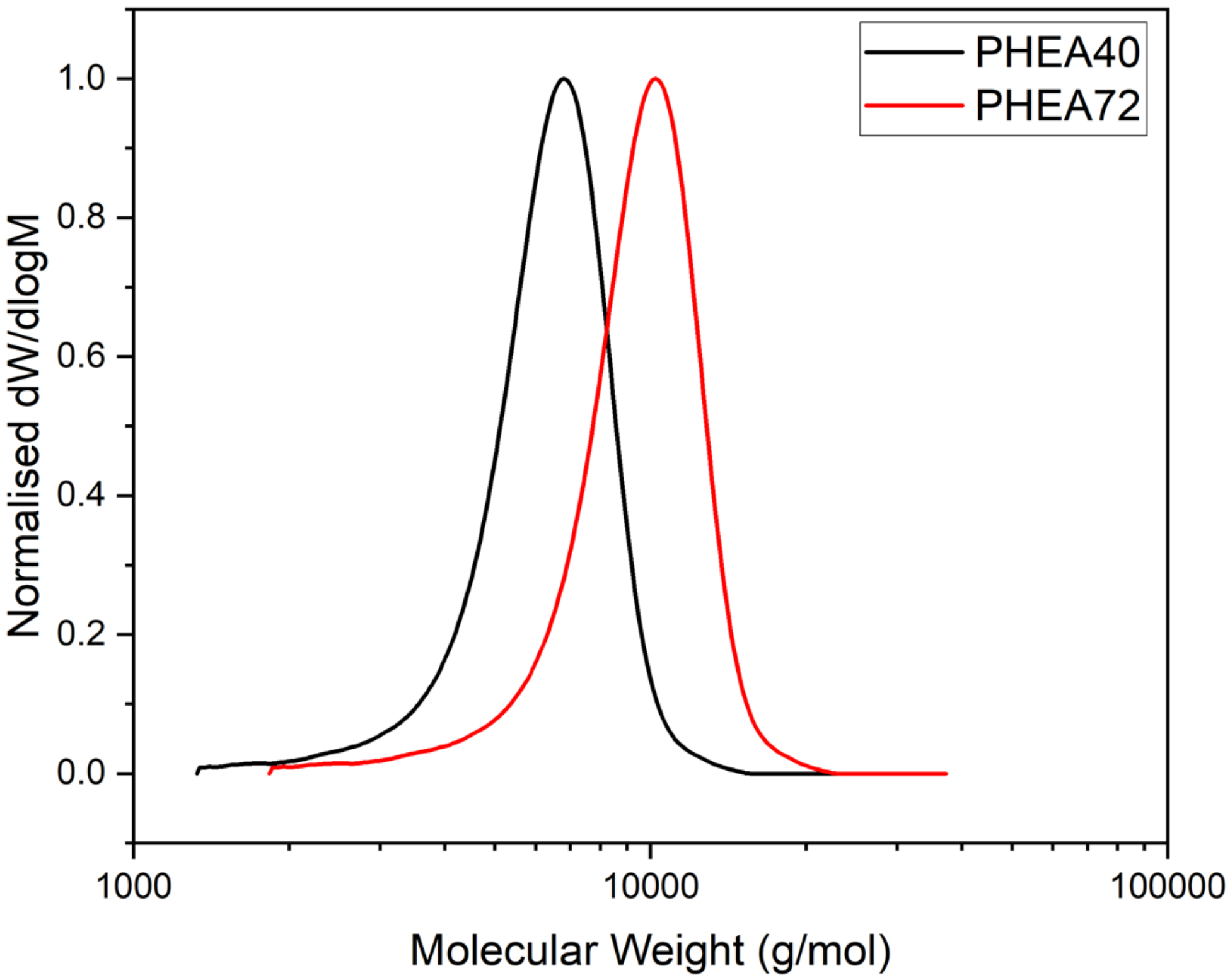

Figure S1. – Normalised size exclusion chromatography RI molecular weight distributions of $PHEA_{40}$ and $PHEA_{72}$ obtained in DMF versus PMMA standards.

Polymer	[M]:[CTA]	$M_{n\,(theo)}$ [g mol^{-1}]$^{(a)}$	$M_{n\,(SEC)}$ [g mol^{-1}]$^{(b)}$	$DP_{NMR}$$^{(c)}$	$Đ_M$$^{(b)}$
$PHEA_{40}$	15:1	2260	4900	16	1.14
$PHEA_{72}$	35:1	4560	8700	39	1.11

Table S1. – Polymers prepared

[a]Calculated from feed ratio of monomer to chain transfer agent
[b]Calculated by conventional calibration against poly(methyl methacrylate) standards in DMF as eluent.
[c]Determined from ^{1}H end group analysis

Dynamic Light Scattering Data

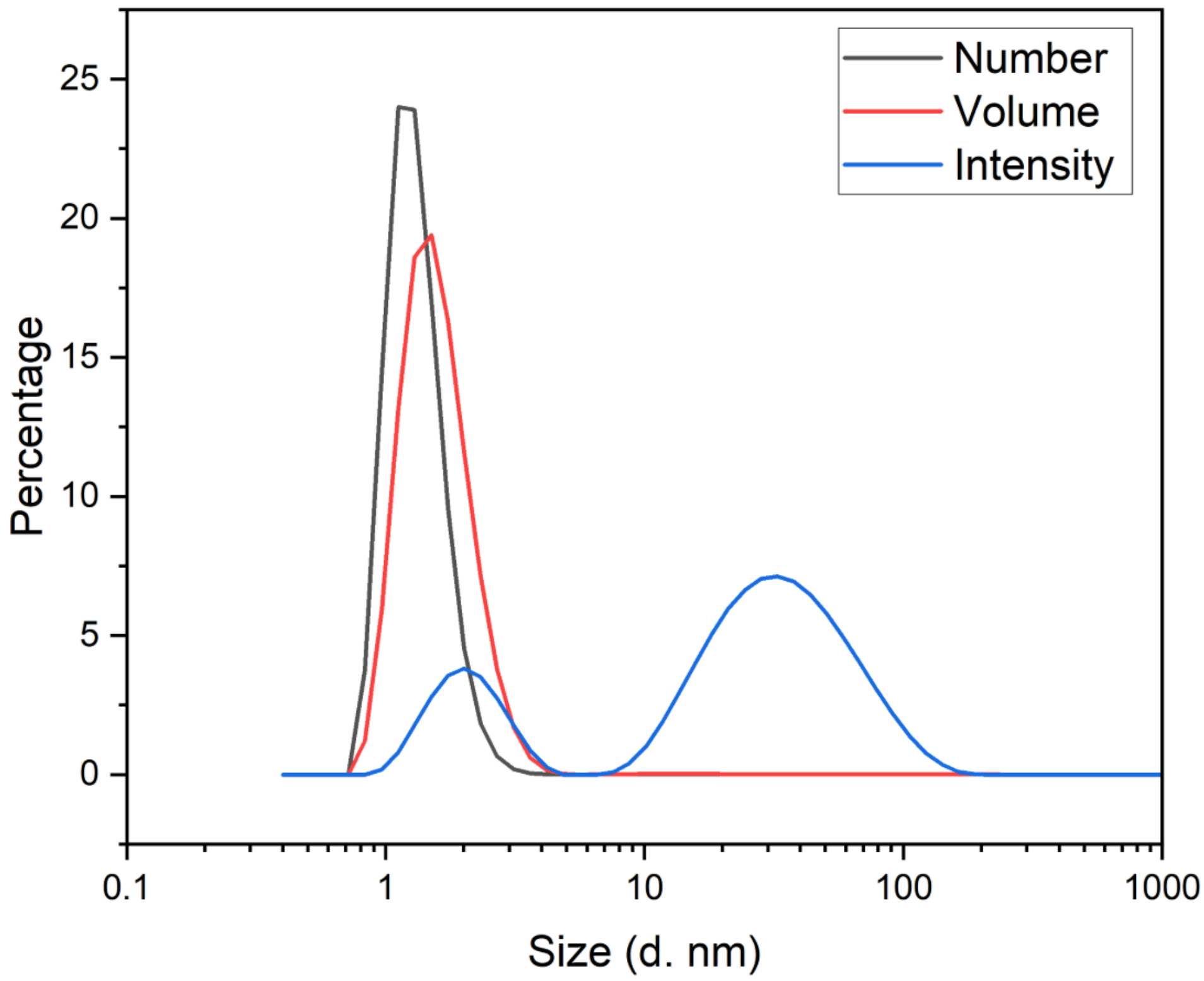

Figure S2. – DLS Size Distribution for 16nm AuNPs

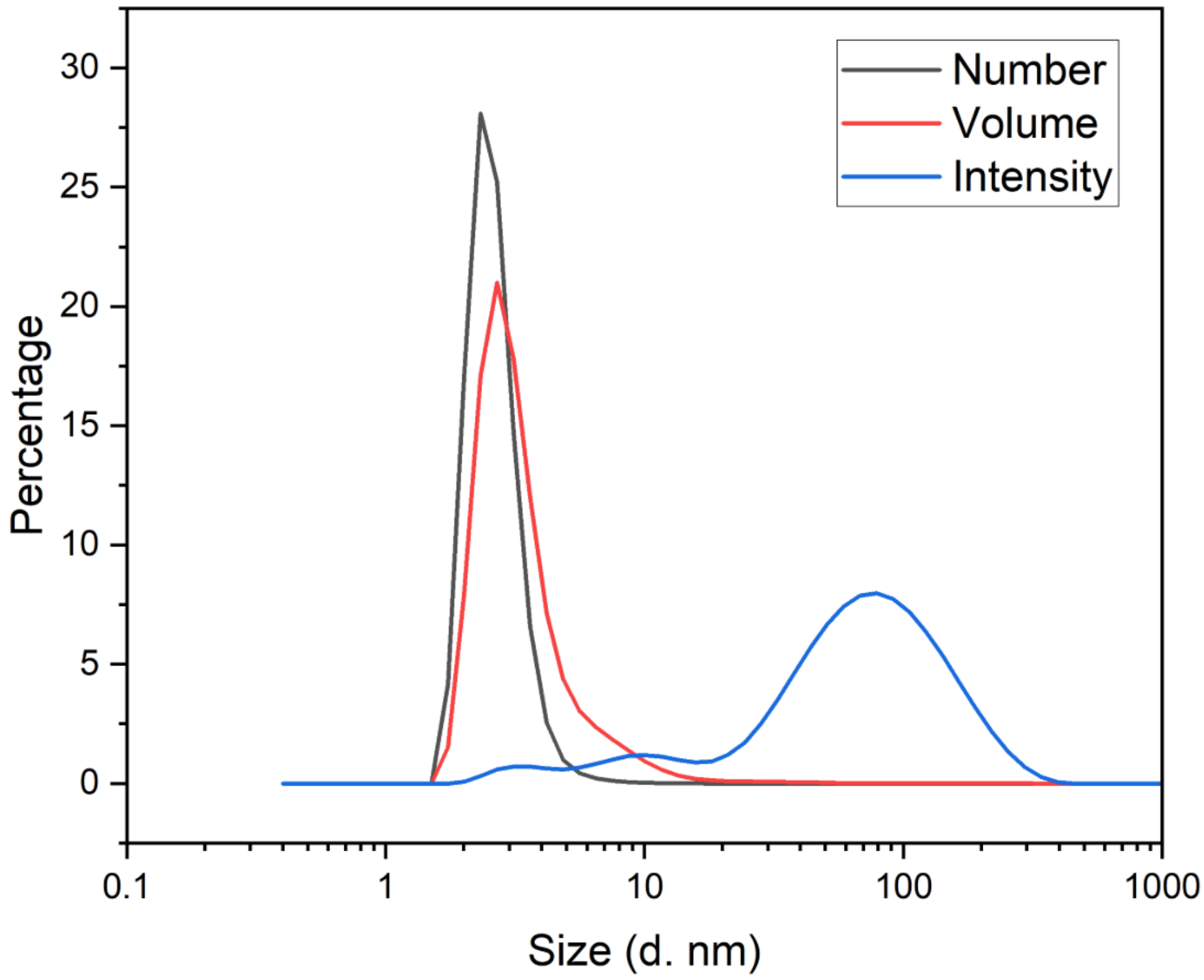

Figure S3. – DLS Size Distribution for Gal-PHEA$_{40}$@AuNP$_{16}$

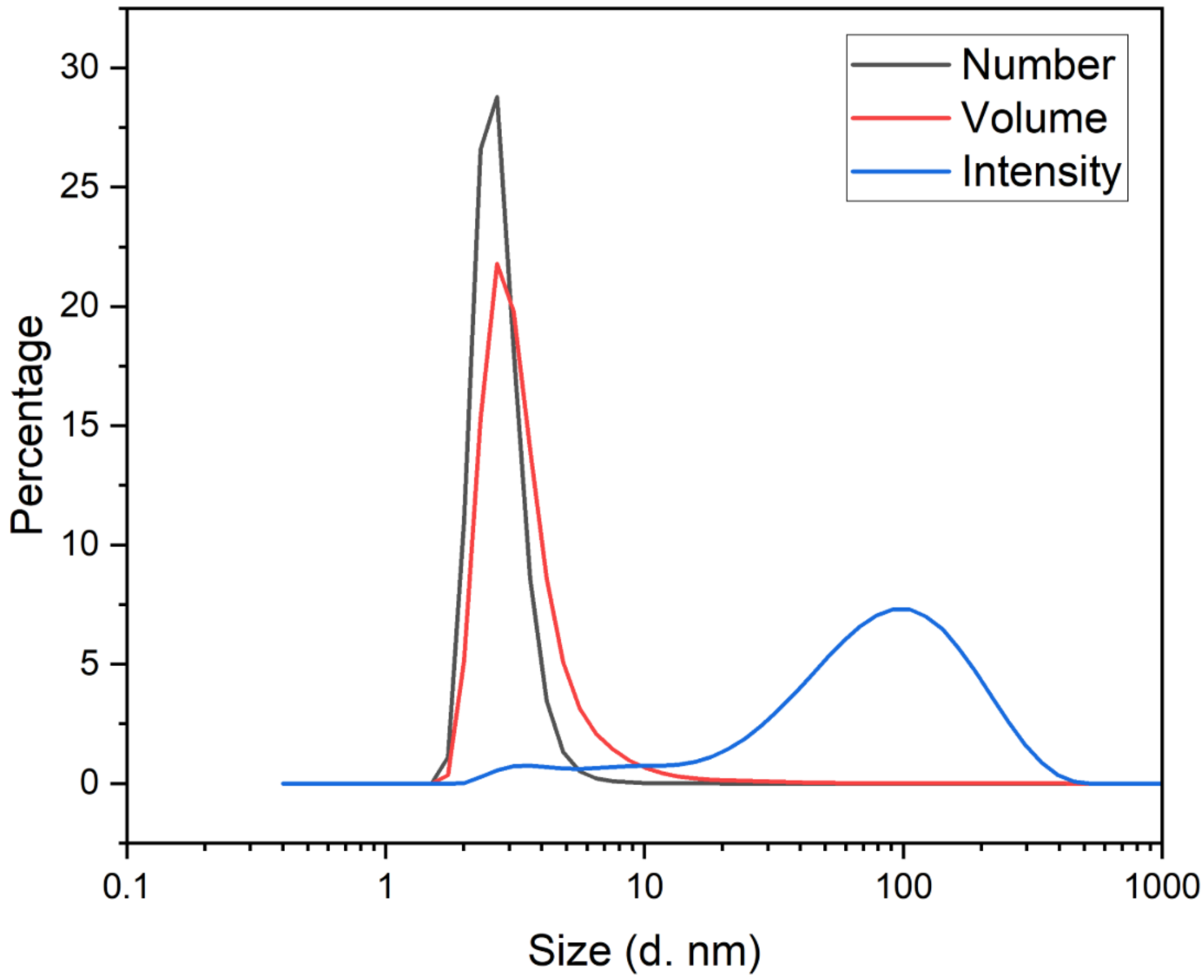

Figure S4. – DLS Size Distribution for Gal-$PHEA_{72}$@$AuNP_{16}$

UV-Vis Data

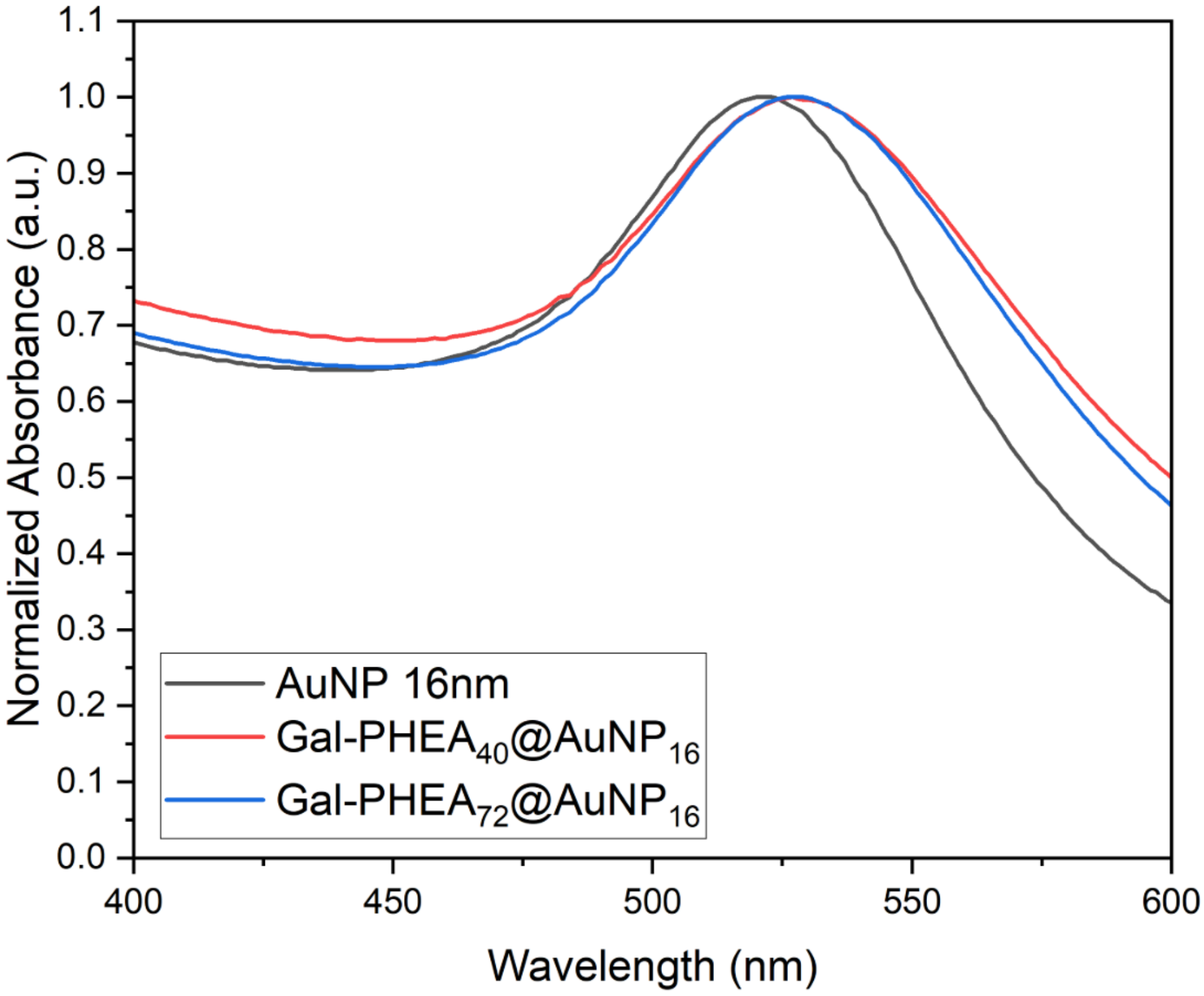

Figure S5. – Normalised UV-vis curve of 16 nm AuNPs, Gal-PHEA$_{40}$@AuNP$_{16}$, Gal-PHEA$_{72}$@AuNP$_{16}$.

Particle	□$_{SPR}$[a] (nm)	DLS[b] (nm)
**AuNPs 16nm**	520	14.24 ± 0.559
**GalPHEA$_{40}$@AuNP$_{16}$**	527	45.34 ± 0.505
**GalPHEA$_{72}$@AuNP$_{16}$**	526	50.74 ± 0.544

Table S2. – UV-Vis test results

[a]Maximum absorption wavelength from the surface plasmon resonance band of the particles

[b]Diameter from dynamic light scattering ± standard error from three measurements.

Lateral Flow Strip Production, Running and Analysis Protocols

The procedure to produce flow-through and lateral flow devices was identical, apart from the deposition of the analyte directly to the nitrocellulose (flow-through), versus application of tests lines to the nitrocellulose (lateral flow).

Protocol for Manufacturing Lateral Flow Strips

This was made according to a previously published procedure.[4] Backing cards were cut to size by removal of 20 mm using a guillotine. Nitrocellulose was added to the backing card by attaching the plastic backing of the nitrocellulose to the self- adhesive on the card. The wick material was then added to the backing card so it overlaps with the nitrocellulose by ~5 mm. The lateral flow strips were cut to size of width 2-3 mm.

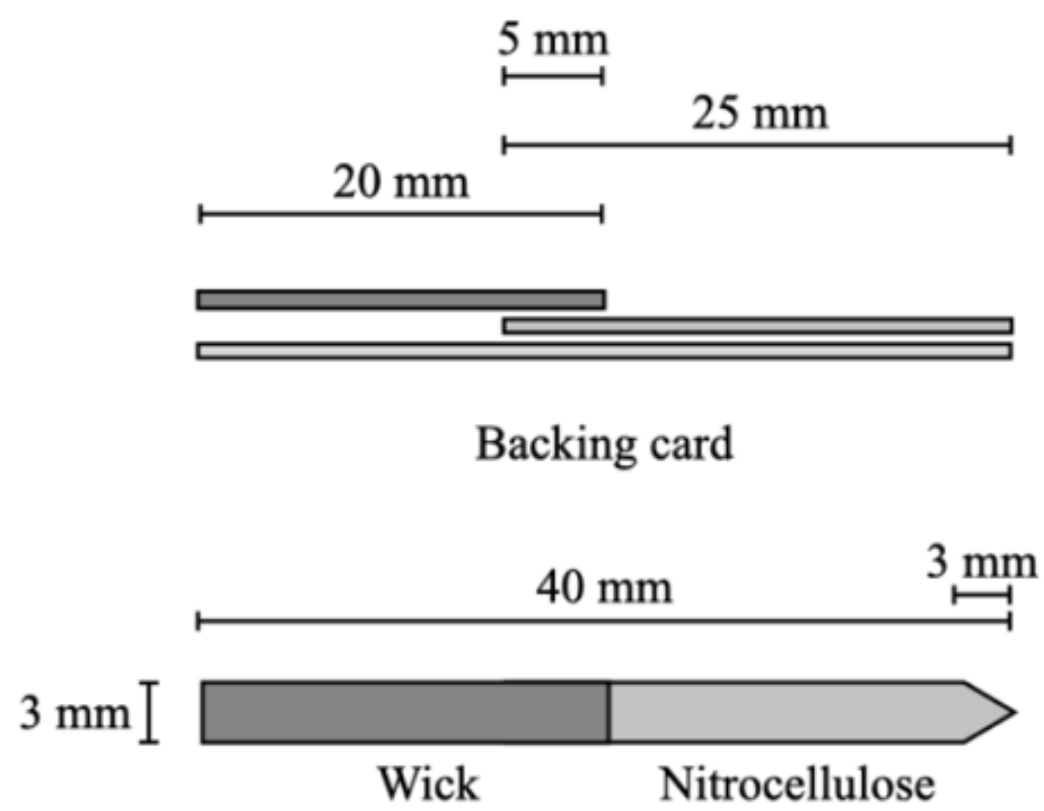

Figure S6. – Lateral flow strip dimensions[4]

Assay Buffer – 10x HEPES buffer (10% PVP400) in 100 mL H_2O

Synthesised according to previously published procedure.[4] 2.38 g (100 mmol dm^{-3}) of HEPES, 8.77 g (1.50 mol dm^{-3}) of NaCl, 0.011 g (1.0 mmol dm^{-3}) of $CaCl_2$, 0.8 g (0.8% w/v., 123 mmol dm^{-3}) of NaN_3, 0.5 g (0.5% w/v., 4.07 mmol dm^{-3}) of Tween-20 and 10 g (10% w/v.) of poly(vinyl pyrrolidone)$_{400}$ (PVP_{400}, Average Mw ~40,000) were dissolved in 100 mL of water. The buffer was not pH adjusted.

Protocol for Running Flow-Through Test

This was designed according to a previously published procedure.[4] 1 μL of the chosen analyte either SBA or BSA (0.1-0.5 mg/mL) was manually spotted onto the test strip using a micropipette fitted with 10 μL tip. The analyte was spotted by hand 1 cm away from the non-wick end of the strip. The strips were dried at 20 °C for 30 minutes. The running buffer of total volume 50 μL was made as follows; 12.5 μL AuNPs (OD=4), 5 μL assay buffer – 10 × HEPES buffer, 32.5μL-X water (where X represents the volume of biologically relevant media as seen in table 1). The running solution was then agitated on a roller for 5 minutes. 45 μL of this solution was added to a 0.2 mL PCR tube, standing vertically.

Media Concentration (In 50 μL)	Volume (μL)
0%	0
10%	5
20%	10

40%	20
60%	30
65%	32.5

Table S3. – Designing different concentrations of media

A "v" shape measuring approximately 3 mm was cut into the test strips at the non-wick end. These strips were then inserted into the 0.2 mL PCR tubes, causing them to extend from the top and prevent the test line phase (1 cm from the non-wick end) from being below the solvent line. Each tube included a single test. Triplicate runs of all tests were conducted.

Strips were removed from the tube after 20 mins and were allowed to dry at 20 °C for ~5 minutes. Each test strip was affixed with the test-face facing downwards onto a transparent and colourless sheet of acetate.

Protocol for Test Line Addition for Lateral Flow Test

A volume of 1 μL of 0.5 mg/mL Galɑ1-3Galβ1-4GlcNAc-Bovine Serum Albumin conjugate (Galɑ1-3Galβ1-4GlcNAc-BSA) test line solution was manually spotted onto the test strip using a micropipette equipped with a 10 μL tip. The test line was deposited approximately 1 cm away from the non-wick end of the strip. The strips were dried at 20 °C for 30 minutes.

Protocol for Running Lateral Flow Test

This was designed according to a previously published procedure.[4] The running buffer, with a total volume of 50 μL, was prepared by combining 12.5 μL of AuNPs (OD = 4), 5 μL of lateral flow assay

– 10x HEPES buffer, 2.5 μL of 0.1 mg/mL protein (SBA/BSA), and 30 μL – X (X represents the volume of additional media (saliva, urine, blood) as shown in table S2). Next, the running solution was vigorously agitated on a roller for a duration of 5 minutes. 45 μL of this solution was introduced into a vertically positioned 0.2 mL PCR tube.

Media Concentration<br>(In 50 μL)	Volume (μL)
0%	0
10%	5
20%	10
40%	20
60%	30

Table S4. – Designing different concentrations of media

A "v" shape measuring approximately 3 mm was carved into the test strips at the non-wick end. These strips were then inserted into the 0.2 mL PCR tubes, causing them to extend from the top and prevent the test line (1 cm from the non-wick end) from being below the solvent line. Each tube included a single test. Triplicate runs of all tests were conducted. After 20 minutes the tests were removed and allowed to dry at 20 °C for 2-5 minutes. Each test strip was affixed with the test-face facing downwards onto a transparent and colourless sheet of acetate.

Standard Protocol for Lateral Flow and Flow Through Strip Analysis

The acetate sheets were scanned using a Canon IJ Scan Utility Lite to a pdf file that

was converted to a jpeg, scans were taken within 1 hour of strip drying. The jpeg was analysed in ImageJ 1.51 using the plot profile function to create a data set exported to Microsoft Excel for Mac. The data was exported to Origin 2019 64Bit and trimmed to remove pixel data not from the strip surface. The data was aligned and the triplicates averaged (mean). The data was then reduced by number of groups to 100 data points (nitrocellulose and wick) and plotted as grey value (scale) vs

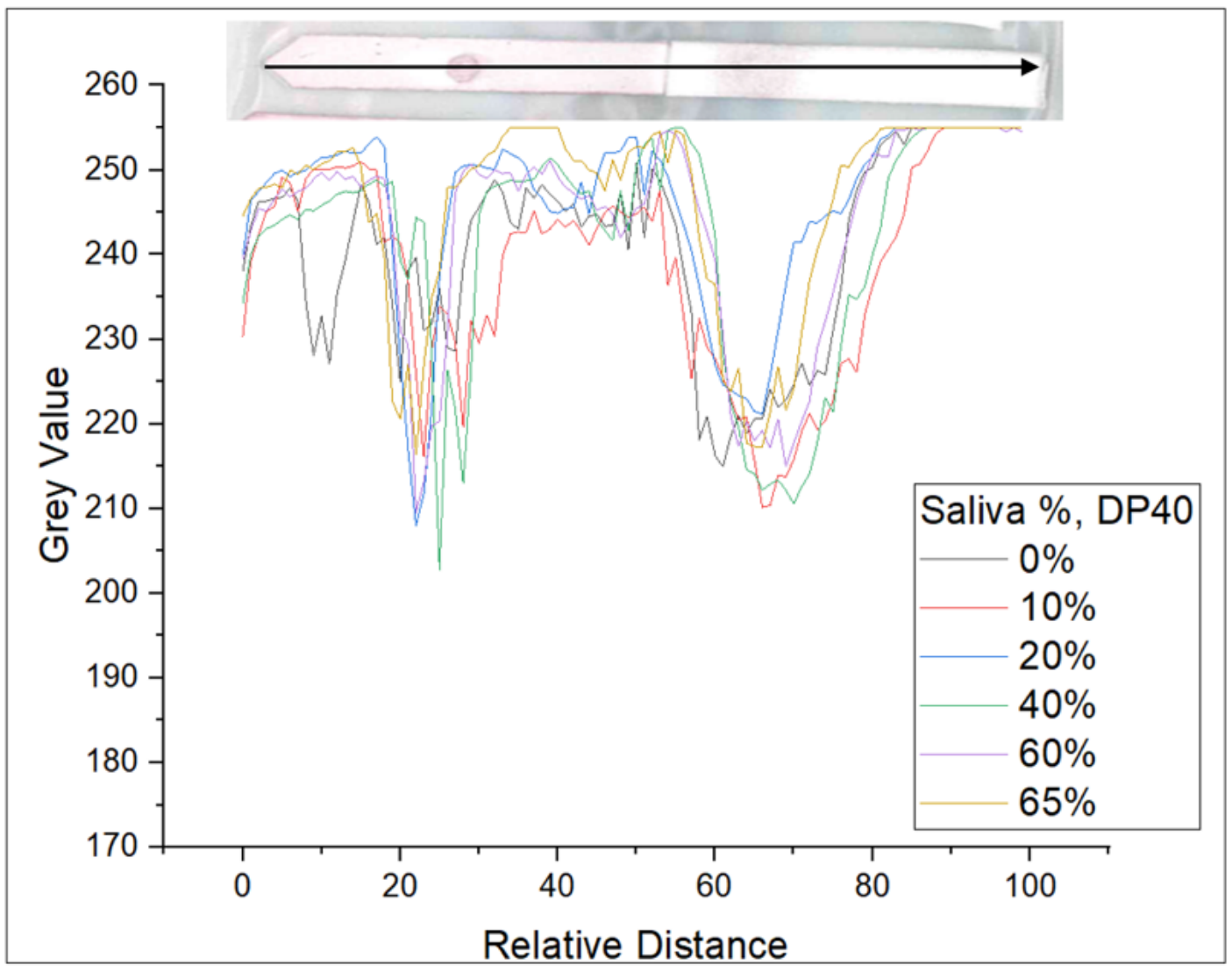

Relative distance along the 100 data points (Figure S2).

Figure S7. – Processed grey value plot (left) and representative dipstick (right)

Lateral Flow Signal to Noise Analysis Relative Distance Pixel

Relative distance pixel 15 to 35 (area around the test line) was averaged (mean) to provide average noise around the test line for strips i.e. pixels that contributed to the signal peak were excluded from the noise calculation. The relative distance pixel was changed for lateral flow with blood to circumvent the dark purple from colloidally unstable AuNPs precipitating and blood aggregating at the solvent front seen on the strips, which is due to the aggregation of AuNPs with SBA in solution. The signal value was determined by selecting the lowest grey value between 15 to

35 relative distance pixels as the test line. Equation 1 was then used to determine the signal to noise ratio.

Error bars were added which account for the standard deviation between the three individual strips. The signal-to-noise ratio (SNR) was calculated independently for each of the three strips in a group using ImageJ gray value plot profiles for each individual strip. Equation 1 was used for each of the strips, and one standard deviation across the three values was calculated in Excel. One standard deviation was then used as the error bars in the bar charts. For graphs representing the signal to noise difference between SBA and BSA, the error for SBA SNR and BSA SNR were added to get total error, illustrated as error bars.

$$\text{Signal to Noise} = \frac{255 - \text{Signal}}{255 - \text{Noise}}$$

Equation S1. – Signal to Noise Ratio (SNR)

$$\text{Signal to Noise Difference} = \text{SNR}_{\text{SBA}} - \text{SNR}_{\text{BSA}}$$

Equation S2. – Signal to Noise Difference between SBA and BSA

Flow-Through Test Results

Percentage %	Saliva		Urine		Blood	
	SBA	BSA	SBA	BSA	SBA	BSA
0%	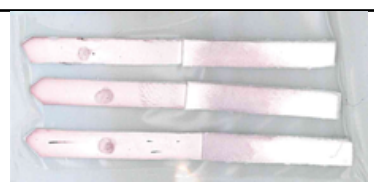	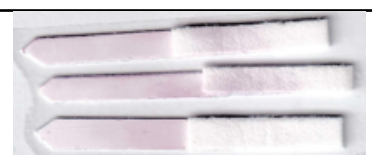	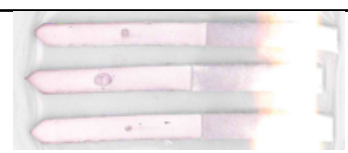	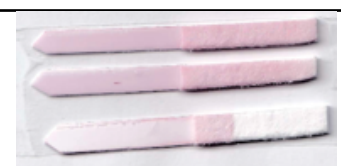	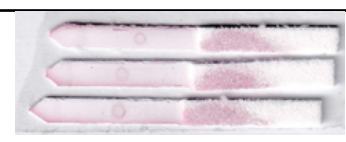	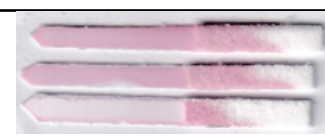
10%	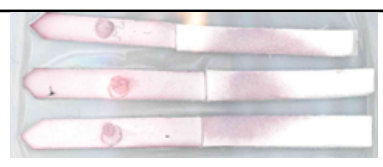	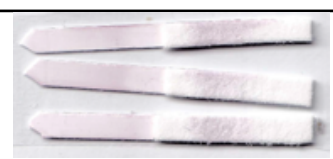	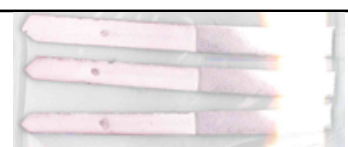	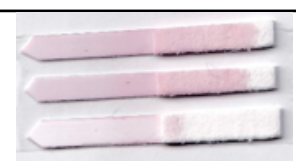	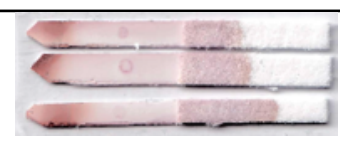	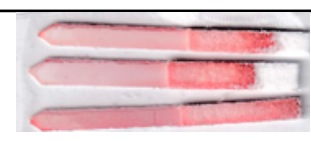
20%	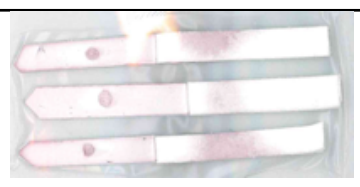	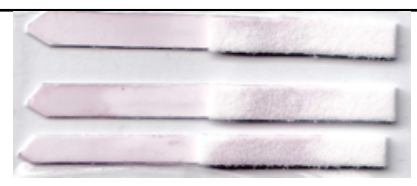	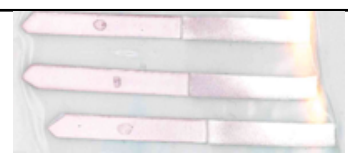	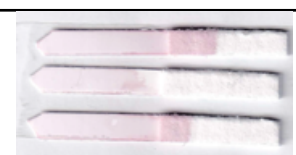	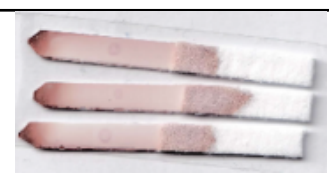	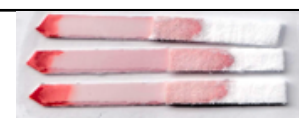
40%	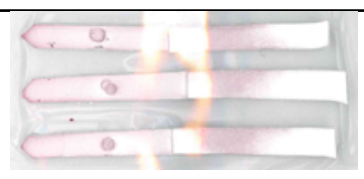	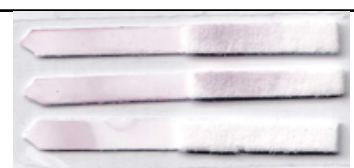	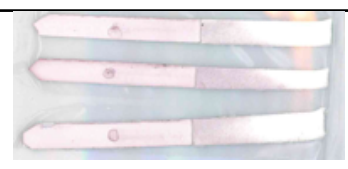	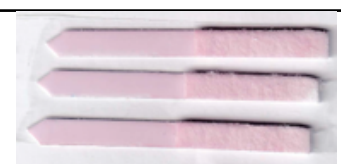	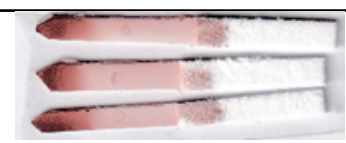	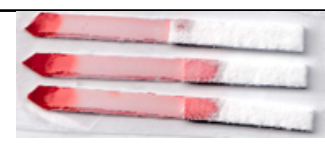
60%	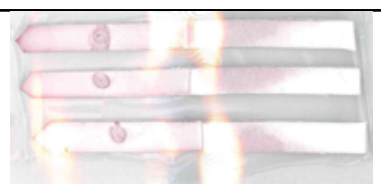	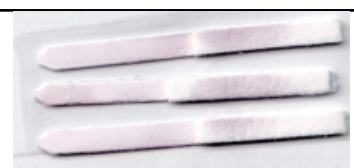	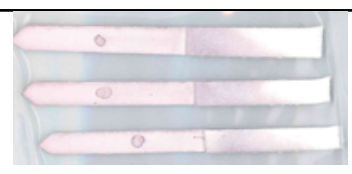	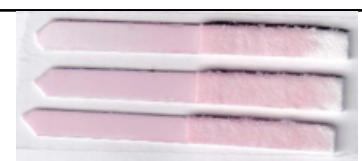	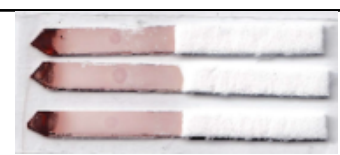	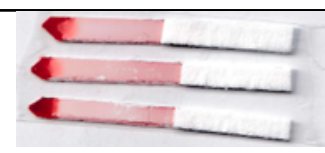
65%	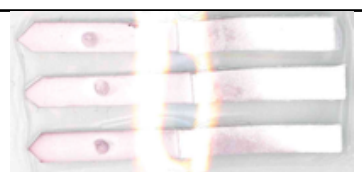	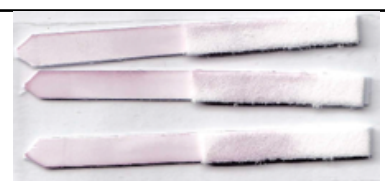	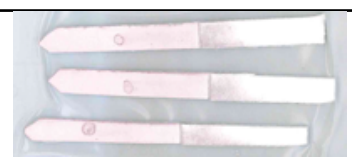	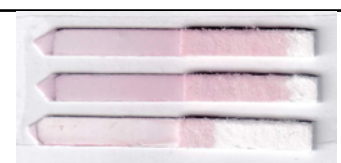	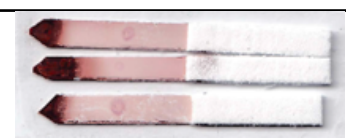	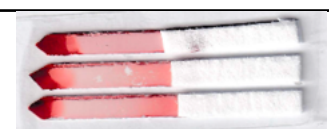

Table S5. – Scans of flow-through strips using Gal-PHEA$_{40}$@AuNP$_{16}$, in doped buffers (varying concentrations of dopant saliva, urine and blood), with a 0.5 mg/mL SBA or BSA test line

Percentage %	Saliva	Urine	Blood
0%	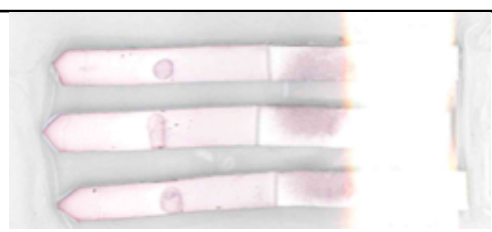	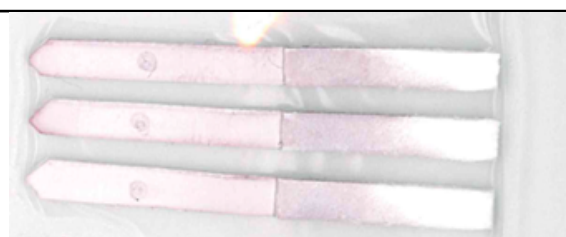	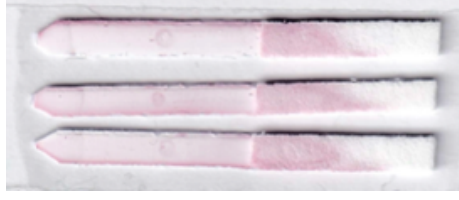
10%	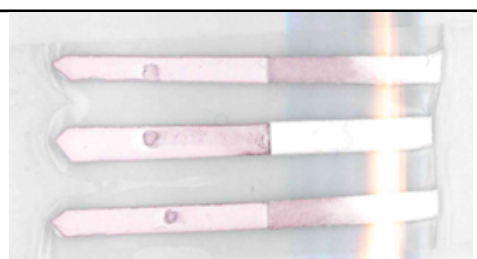	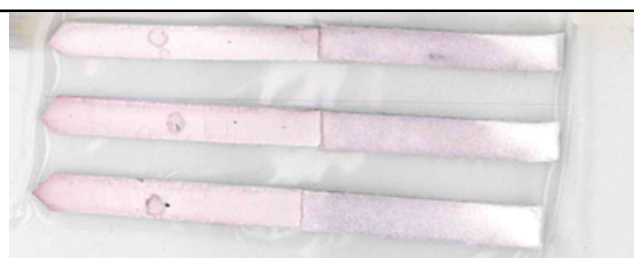	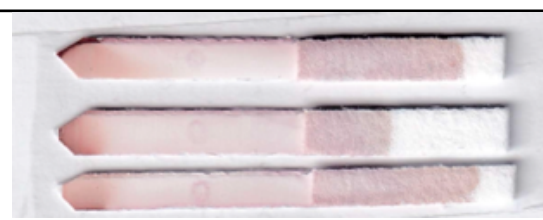
20%	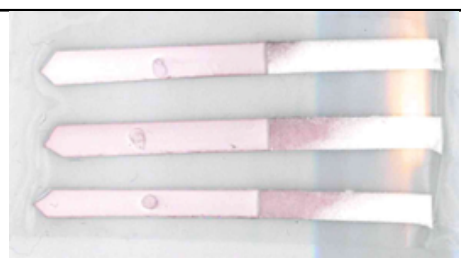	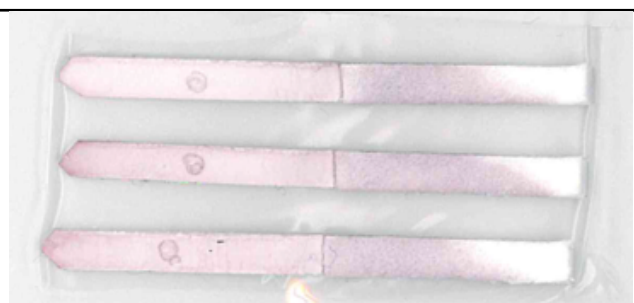	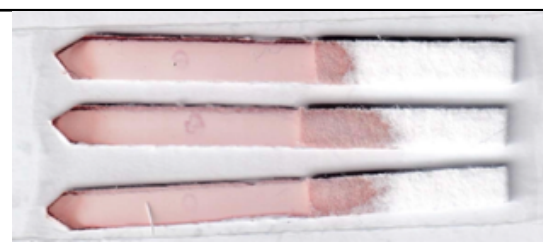
40%	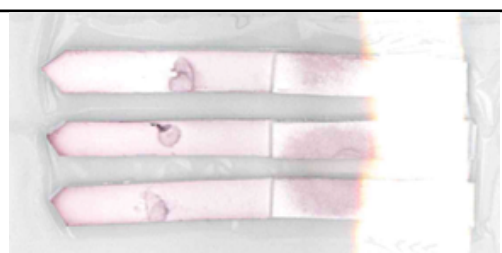	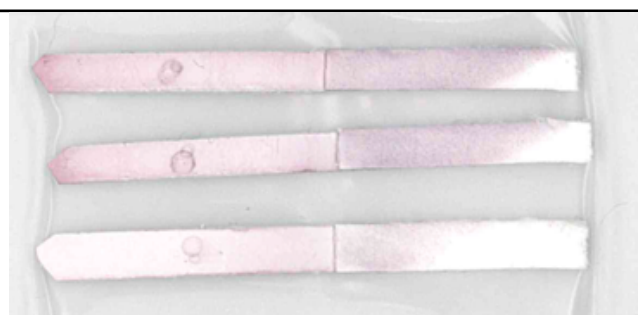	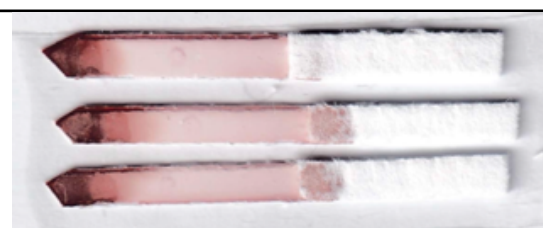
60%	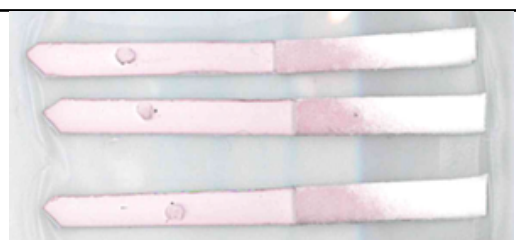	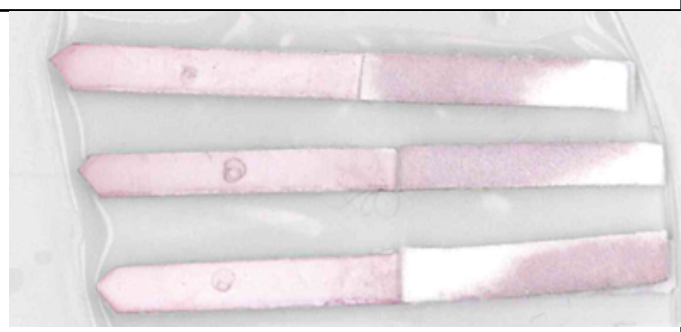	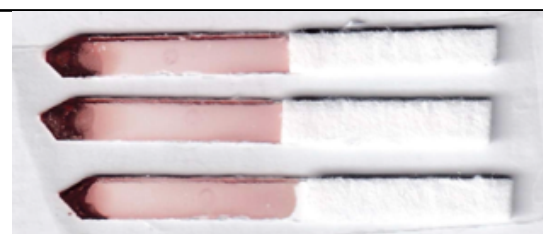
65%	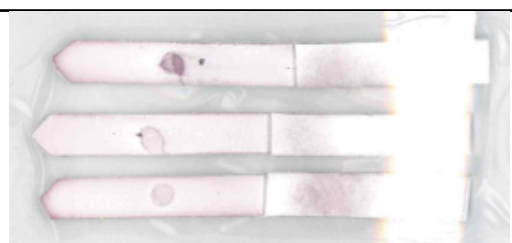	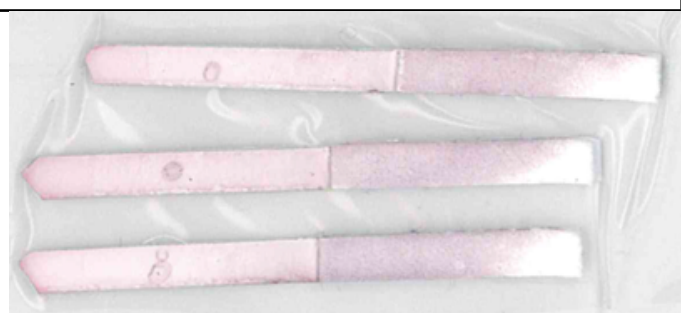	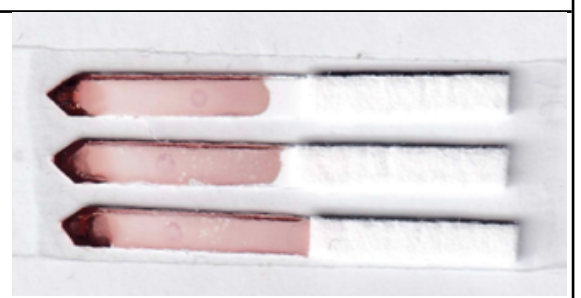

Table S6. – Scans of flow-through strips using Gal-PHEA$_{40}$@AuNP$_{16}$, in doped buffers (varying concentrations of dopant saliva, urine and blood), with a 0.4 mg/mL SBA test line

Percentage %	Saliva	Urine	Blood
0%	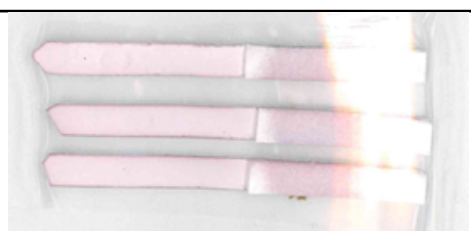	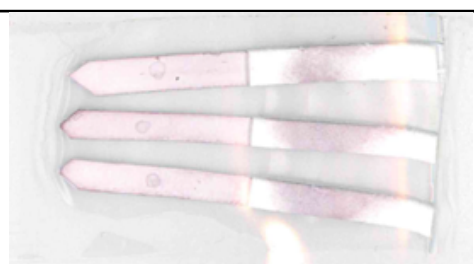	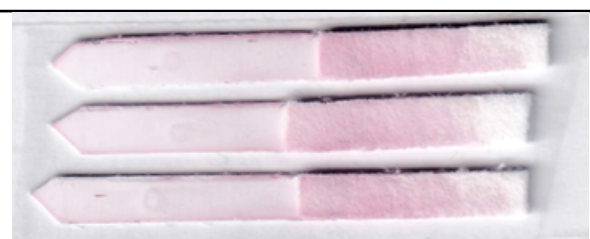
10%	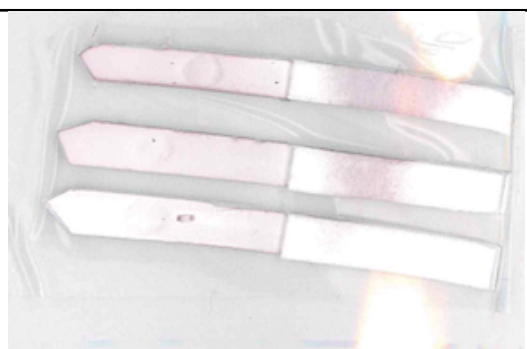	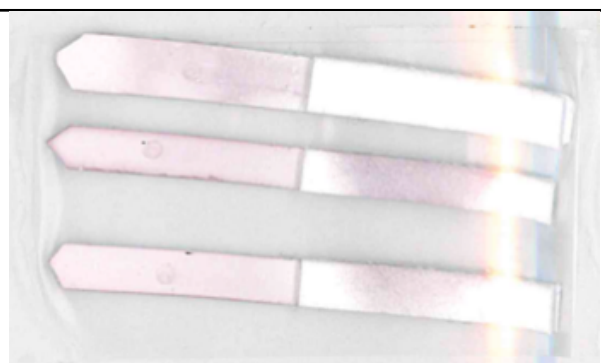	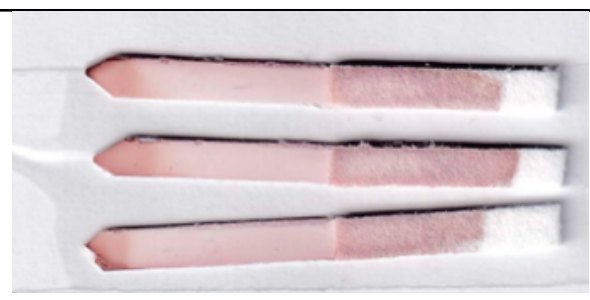
20%	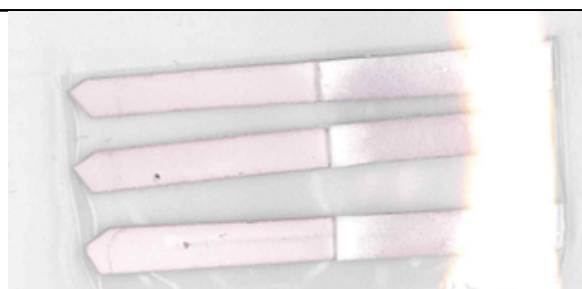	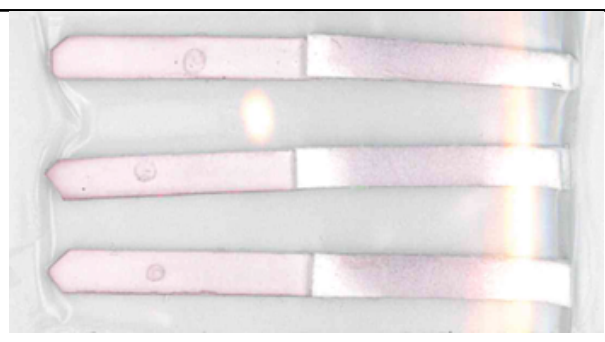	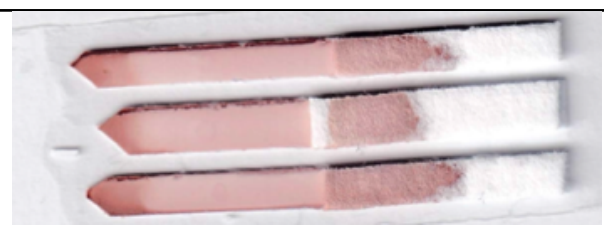
40%	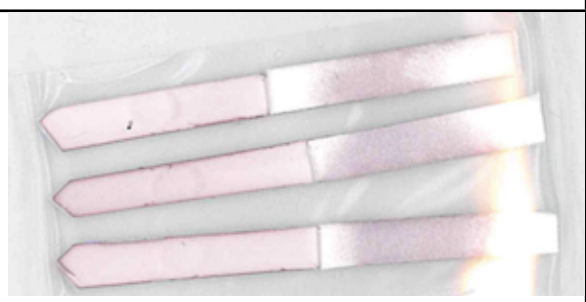	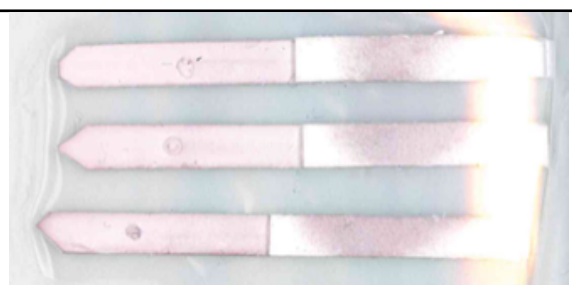	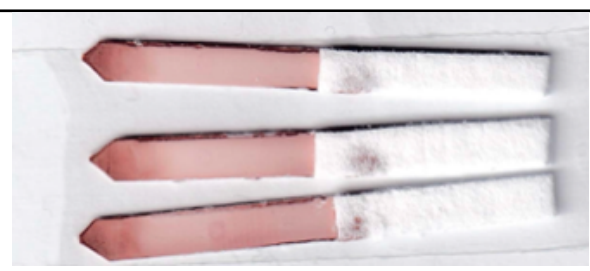
60%	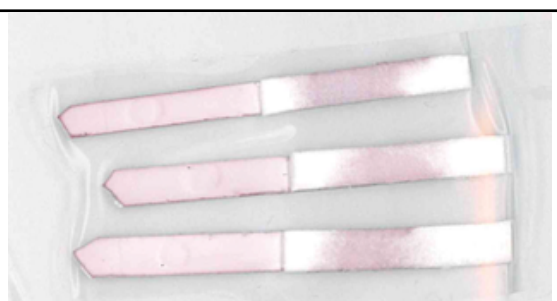	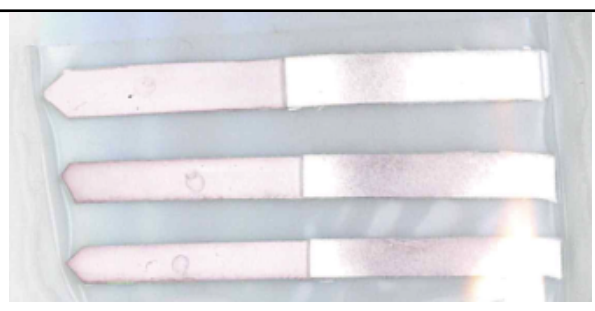	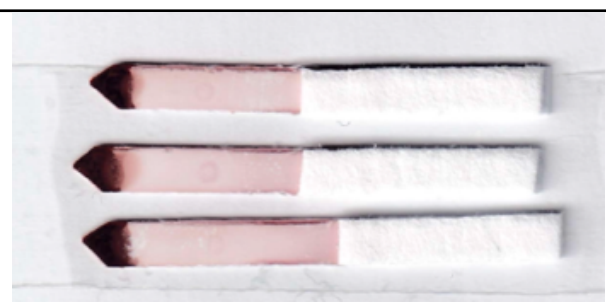
65%	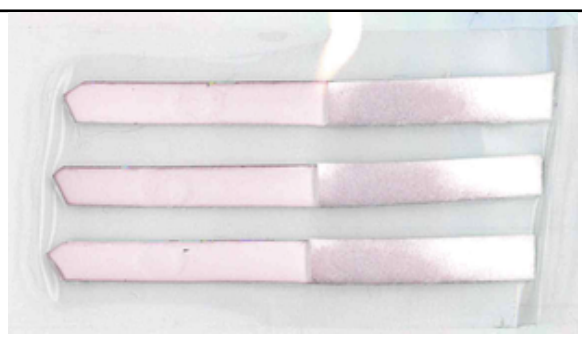	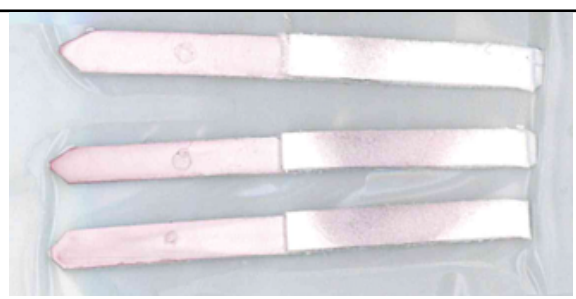	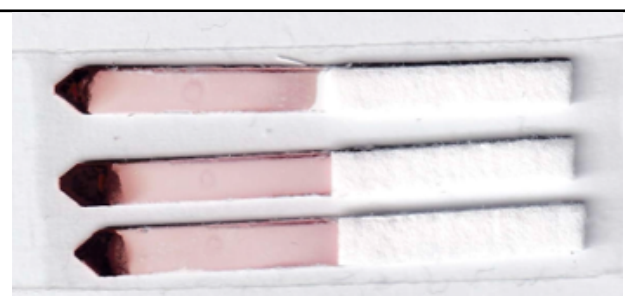

Table S7. – Scans of flow-through strips using Gal-PHEA$_{40}$@AuNP$_{16}$, in doped buffers (varying concentrations of dopant saliva, urine and blood), with a 0.3 mg/mL SBA line

Percentage %	Saliva	Urine	Blood
0%	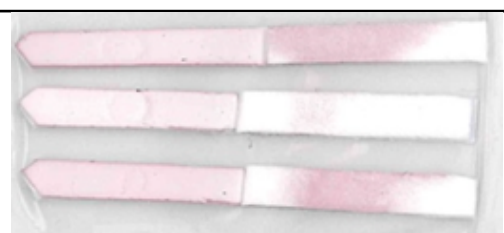	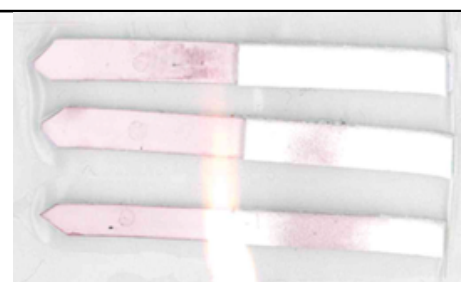	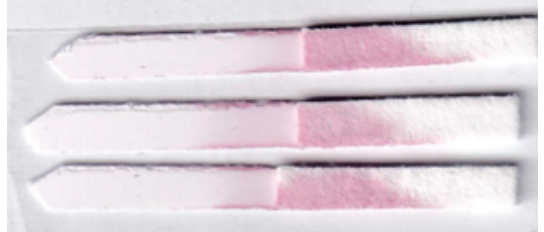
10%	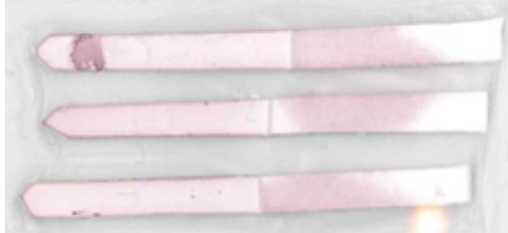	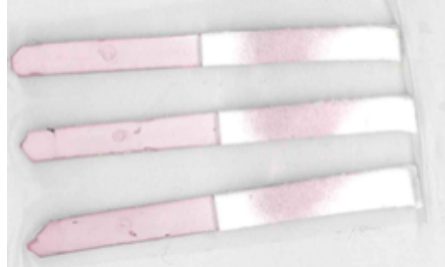	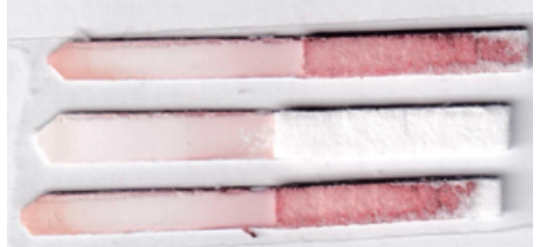
20%	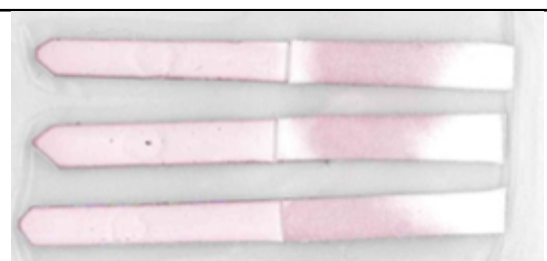	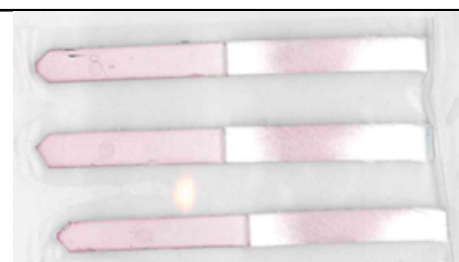	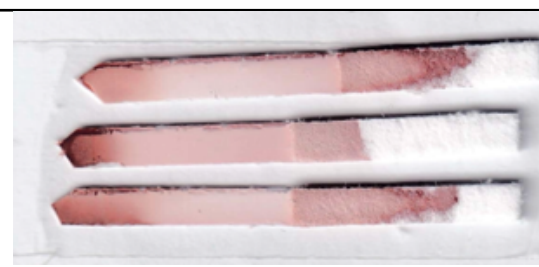
40%	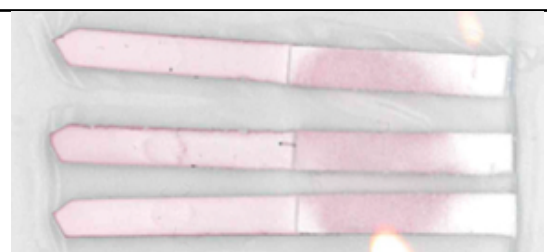	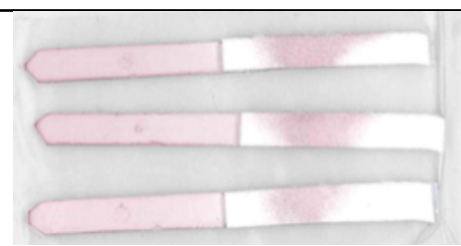	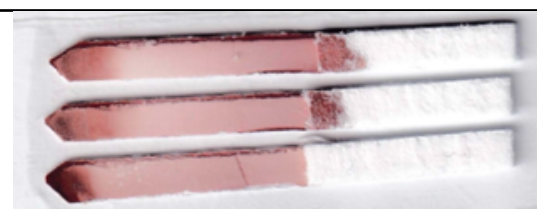
60%	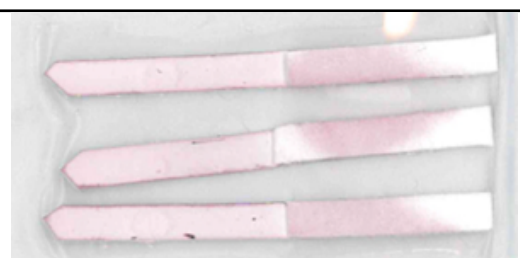	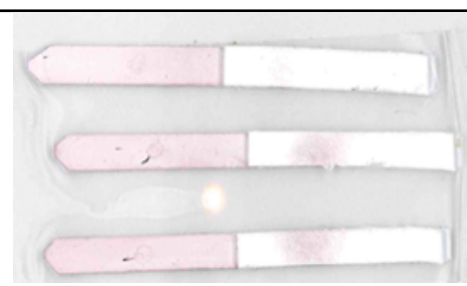	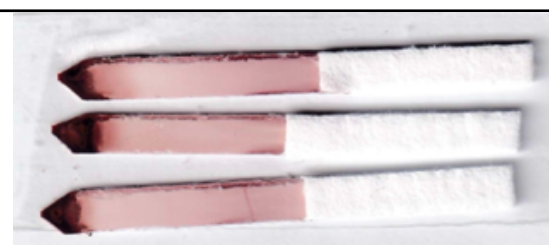
65%	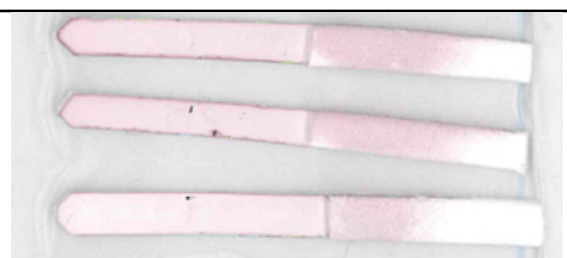	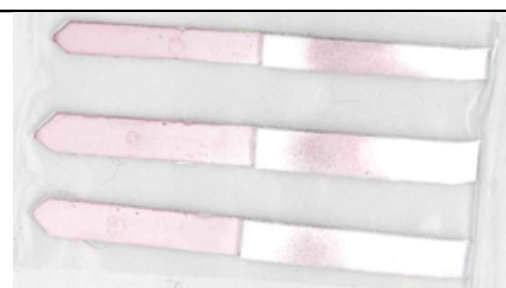	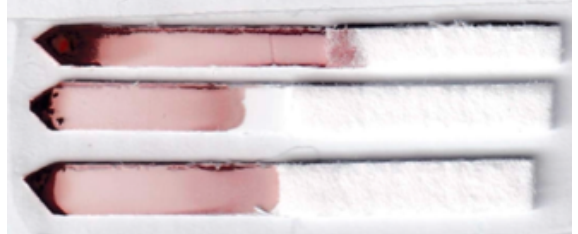

Table S8. – Scans of flow-through strips using Gal-PHEA$_{40}$@AuNP$_{16}$, in doped buffers (varying concentrations of dopant saliva, urine and blood), with a 0.2 mg/mL SBA test line

Percentage %	Saliva	Urine	Blood
0%	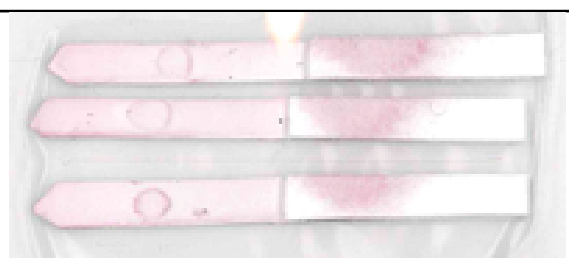	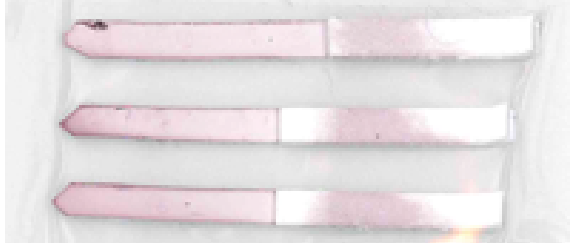	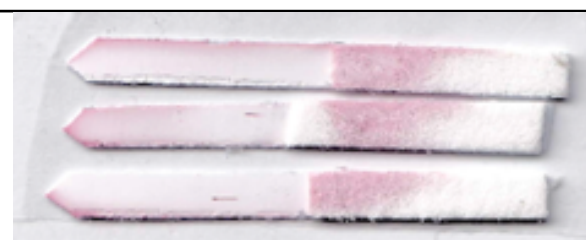
10%	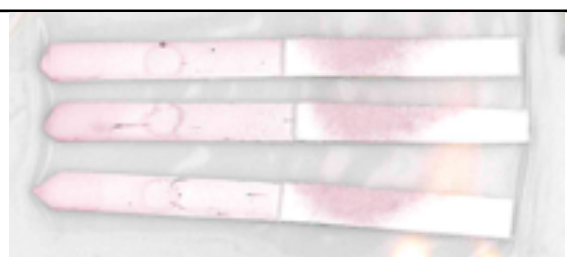	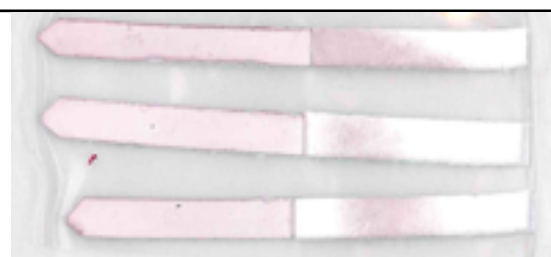	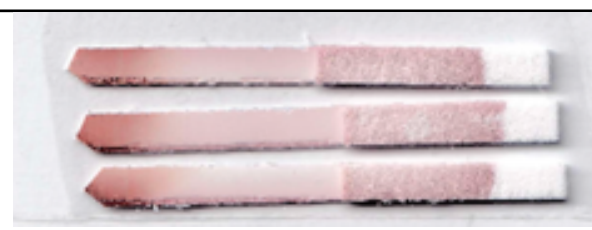
20%	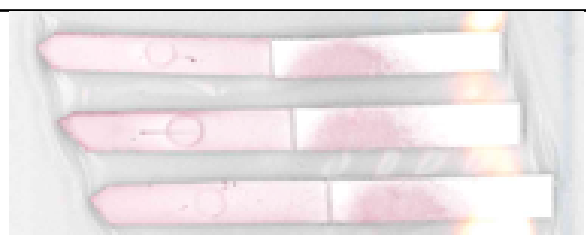	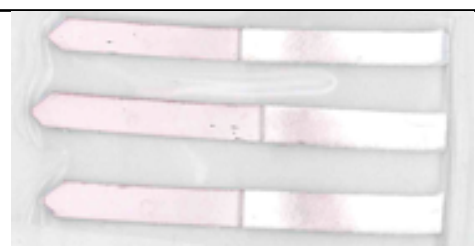	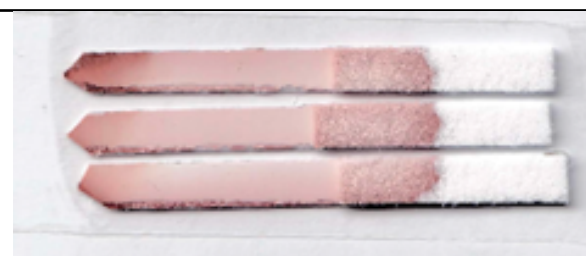
40%	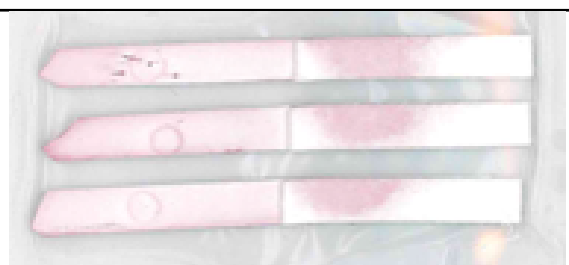	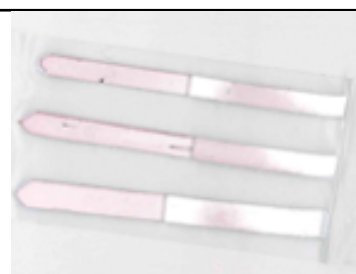	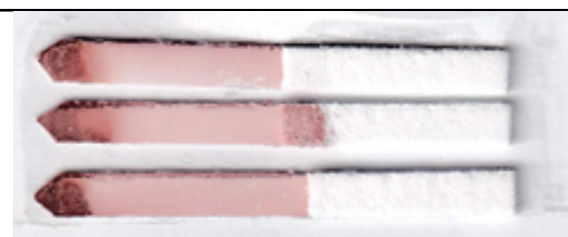
60%	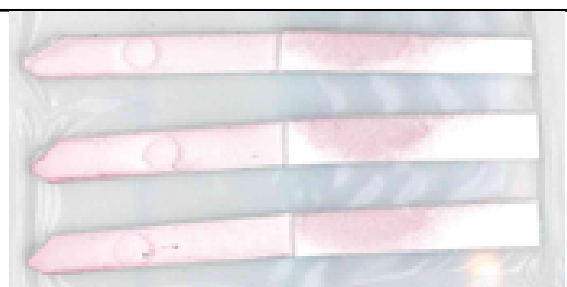	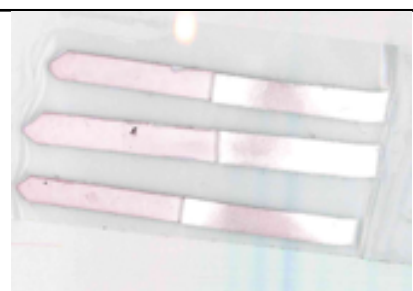	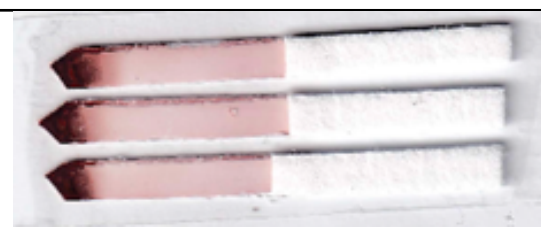
65%	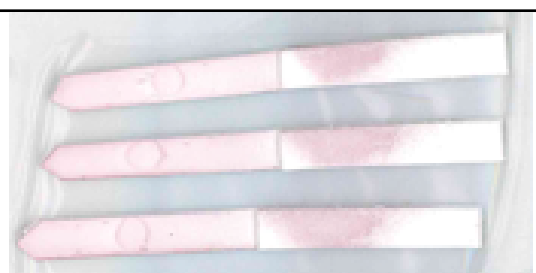	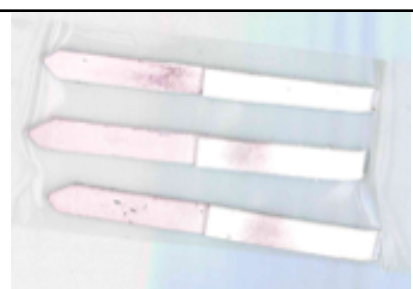	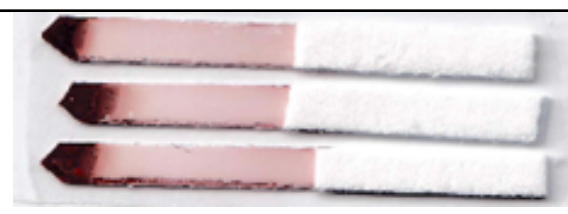

Table S9. – Scans of flow-through strips using Gal-PHEA$_{40}$@AuNP$_{16}$, in doped buffers (varying concentrations of dopant saliva, urine and blood), with a 0.1 mg/mL SBA test line

Percentage<br><br>%	Saliva		Urine		Blood	
	SBA	BSA	SBA	BSA	SBA	BSA
0%	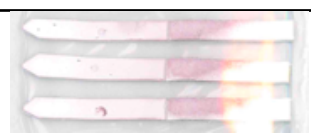	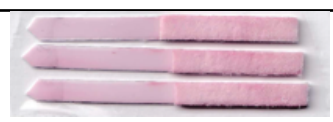	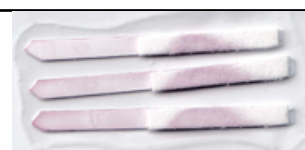	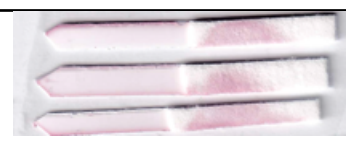	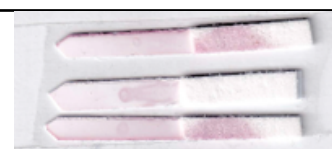	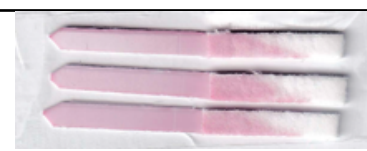
10%	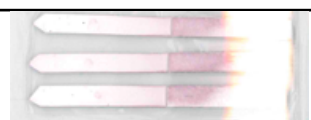	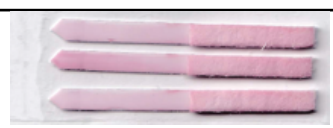	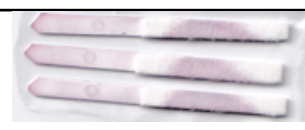	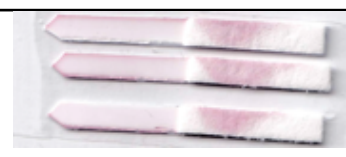	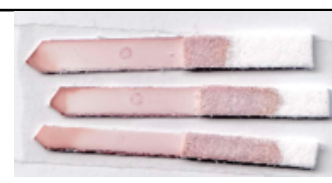	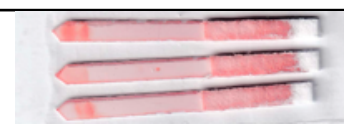
20%	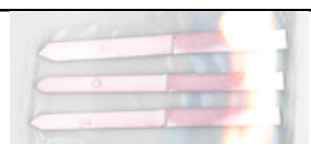	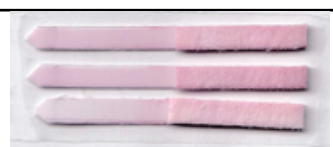	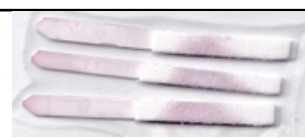	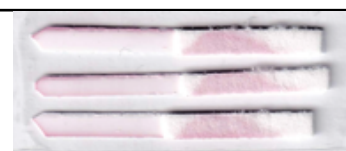	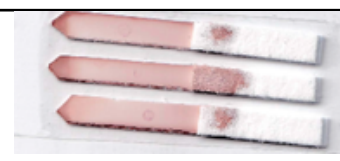	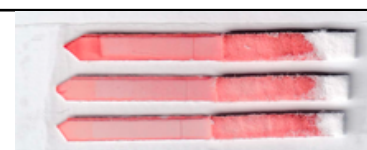
40%	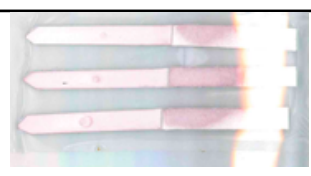	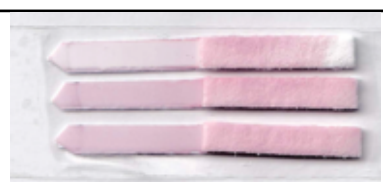	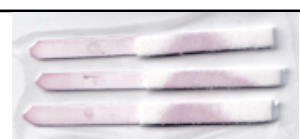	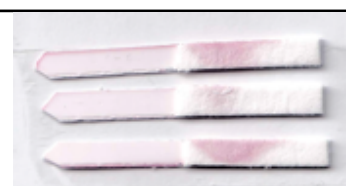	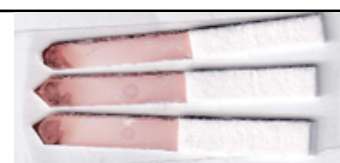	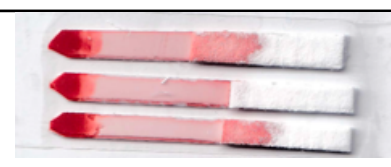
60%	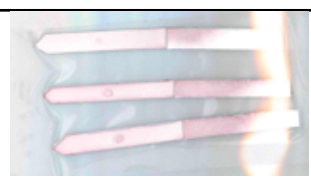	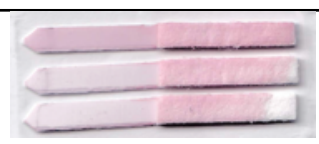	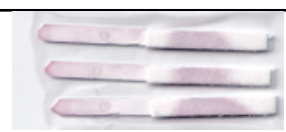	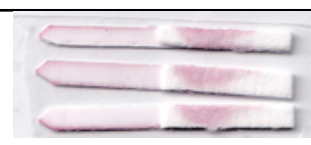	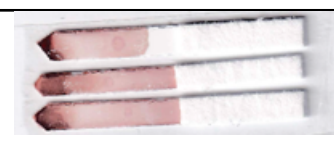	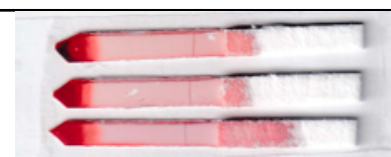
65%	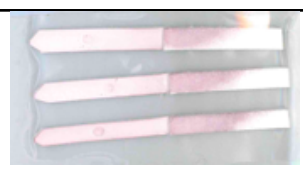	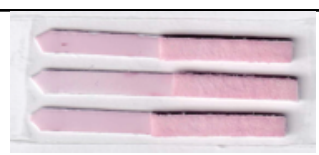	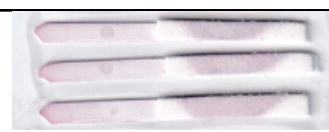	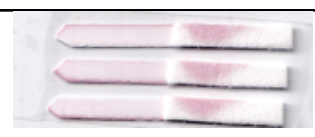	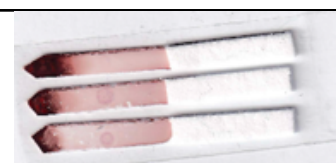	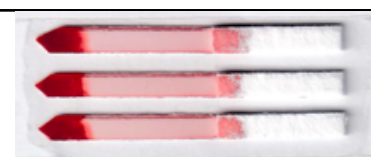

Table S10. – Scans of flow-through strips using Gal-PHEA$_{72}$@AuNP$_{16}$, in doped buffers (varying concentrations of dopant saliva, urine and blood), with a 0.5 mg/mL SBA or BSA test line

Percentage %	Saliva	Urine	Blood
0%	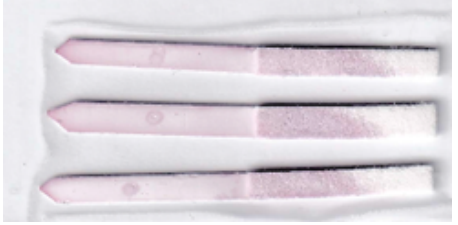	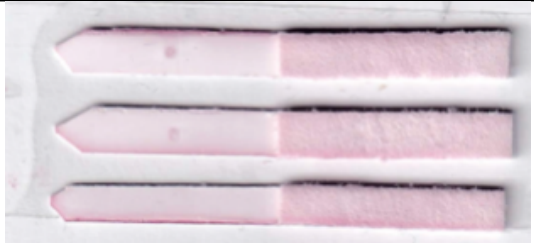	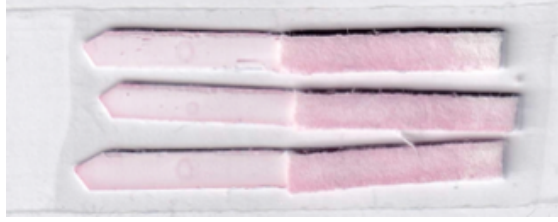
10%	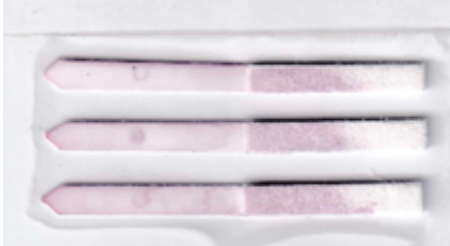	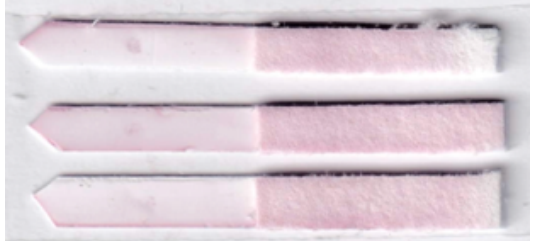	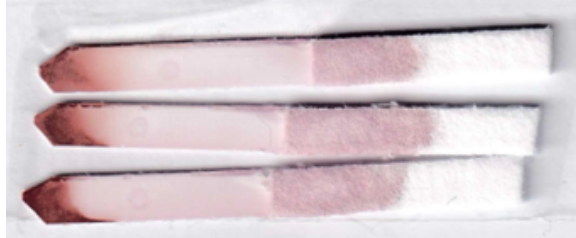
20%	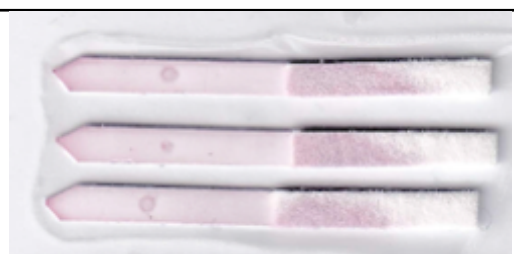	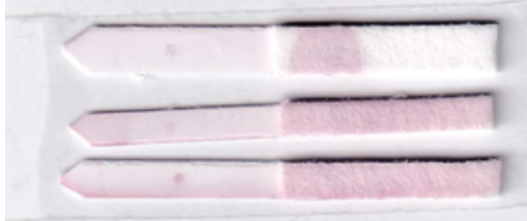	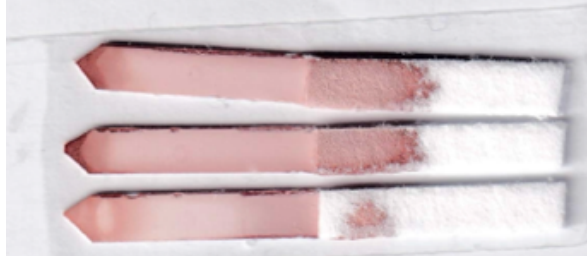
40%	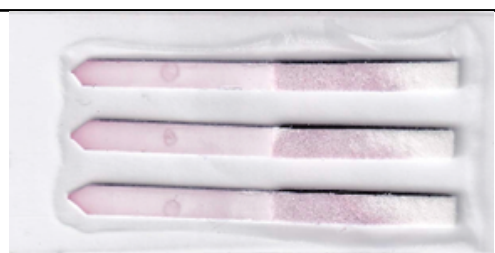	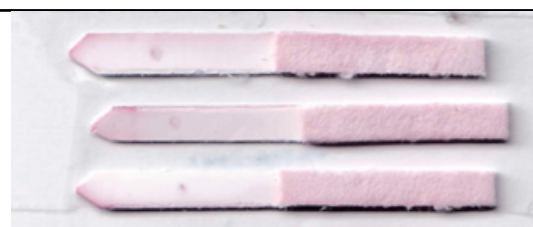	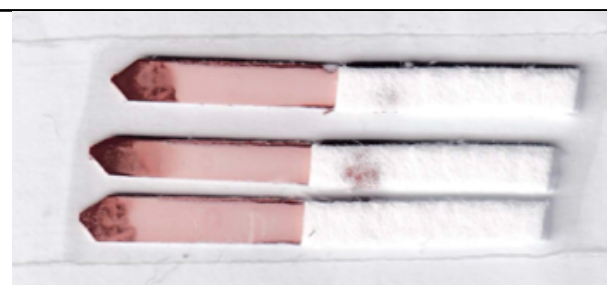
60%	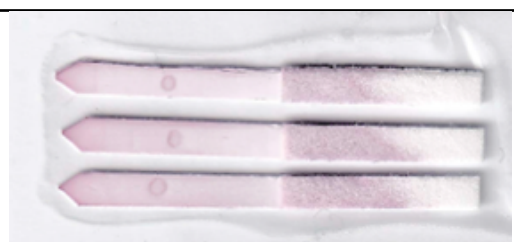	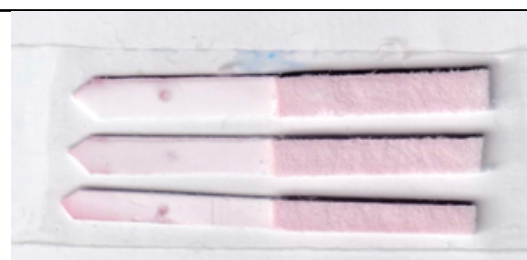	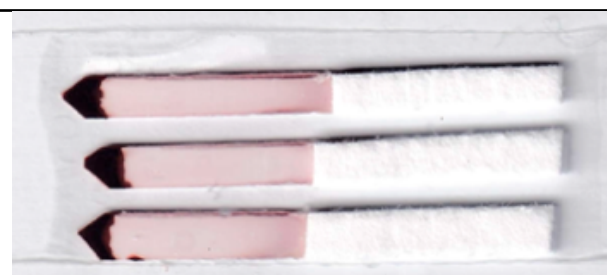
65%	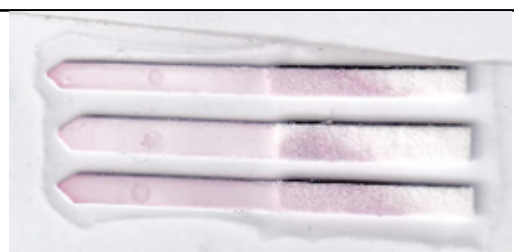	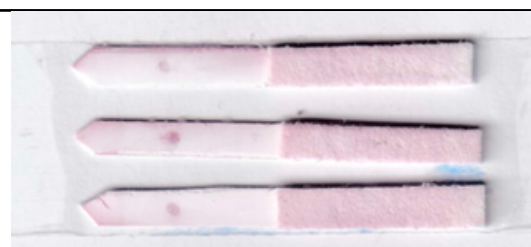	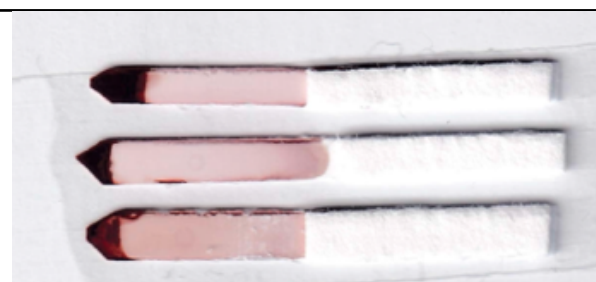

Table S11. – Scans of flow-through strips using Gal-PHEA$_{72}$@AuNP$_{16}$, in doped buffers (varying concentrations of dopant saliva, urine and blood), with a 0.4 mg/mL SBA test line

Percentage %	Saliva	Urine	Blood
0%	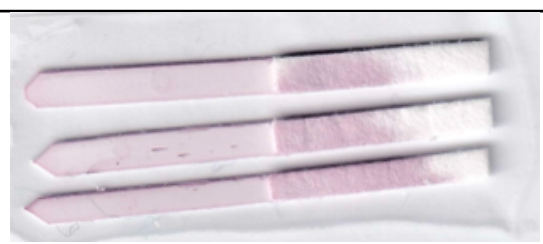	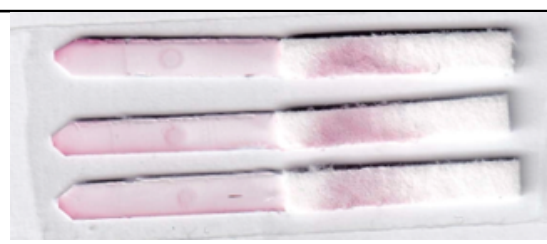	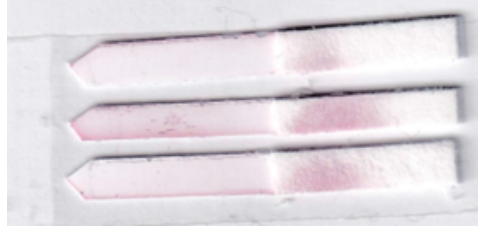
10%	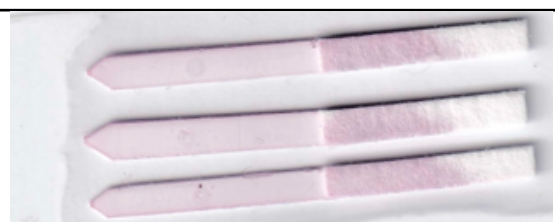	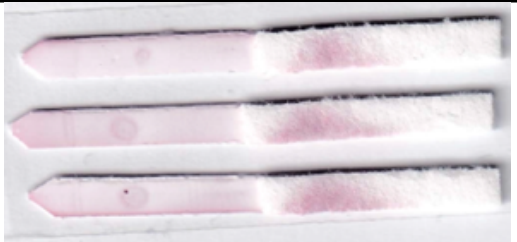	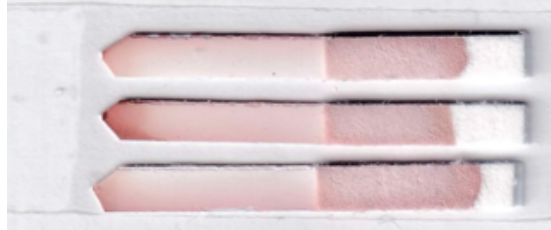
20%	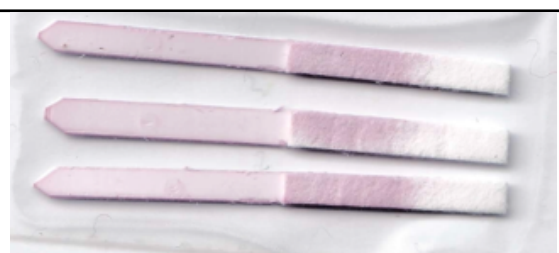	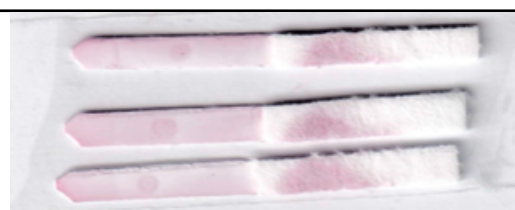	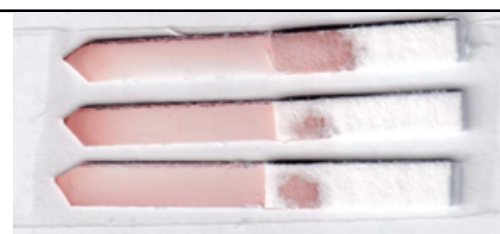
40%	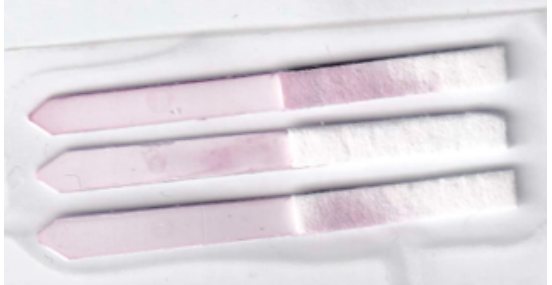	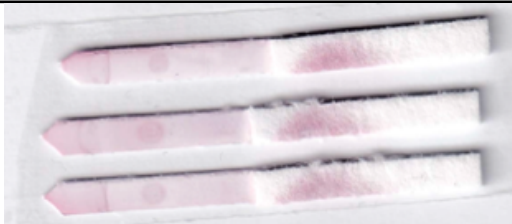	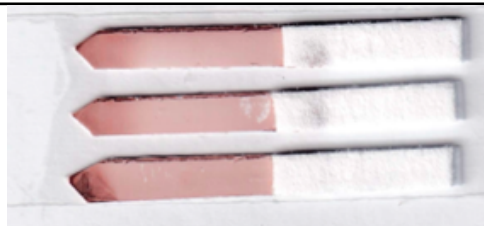
60%	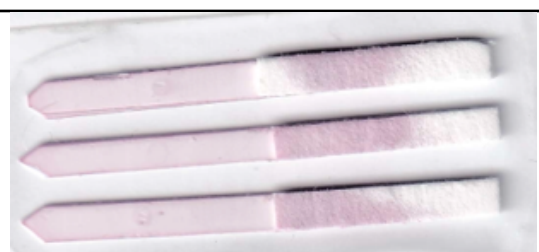	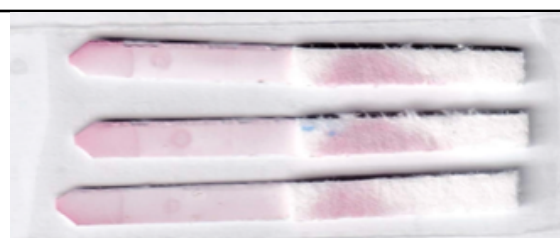	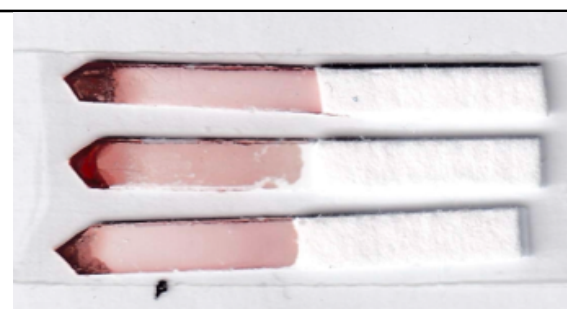
65%	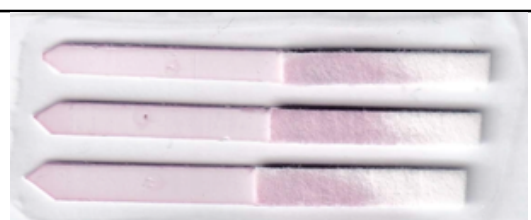	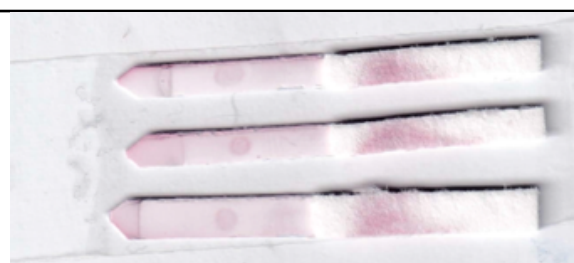	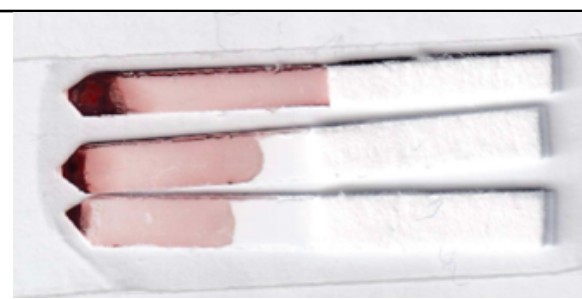

Table S12. – Scans of flow-through strips using Gal-PHEA$_{72}$@AuNP$_{16}$, in doped buffers (varying concentrations of dopant saliva, urine and blood), with a 0.3 mg/mL SBA test line

Percentage %	Saliva	Urine	Blood
0%	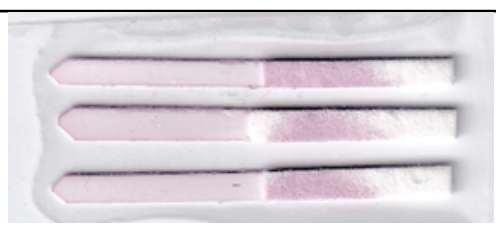	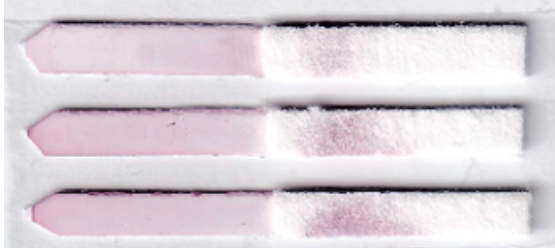	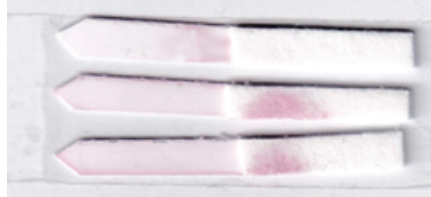
10%	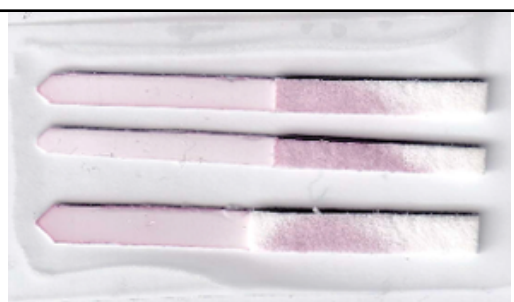	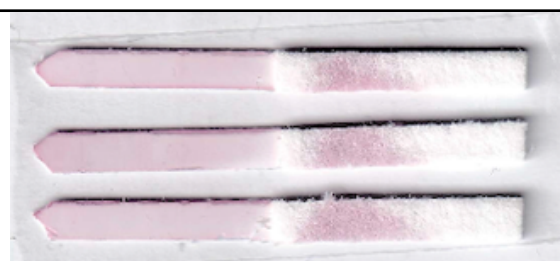	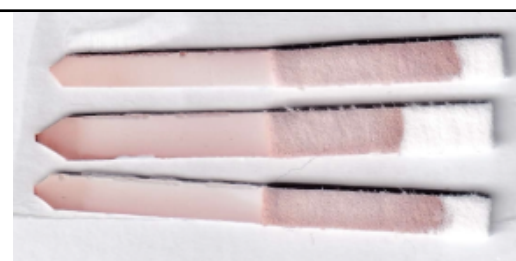
20%	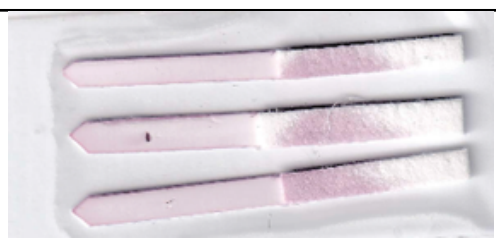	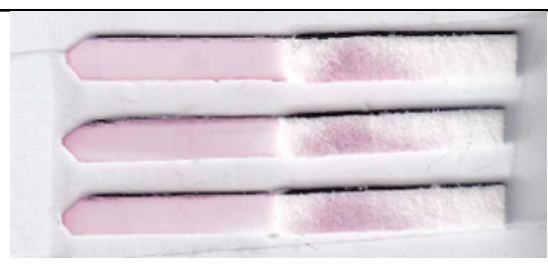	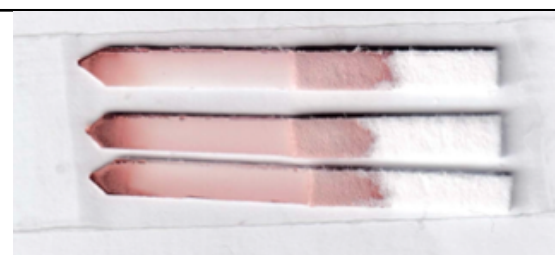
40%	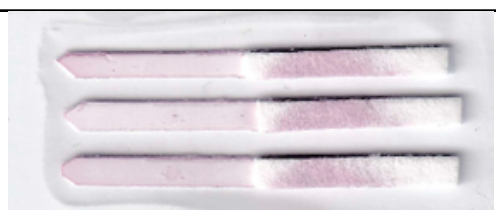	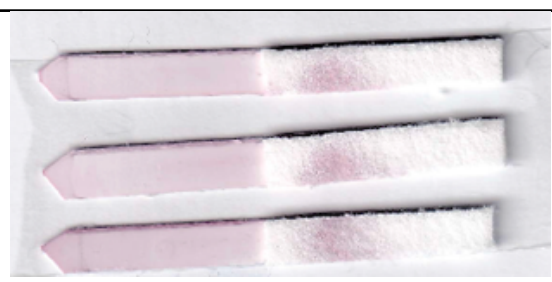	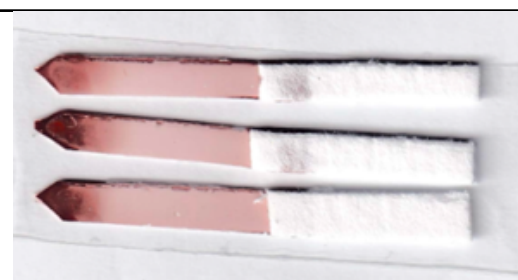
60%	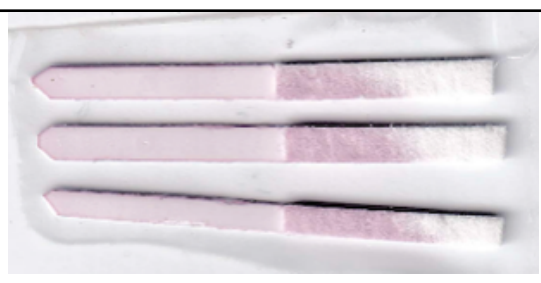	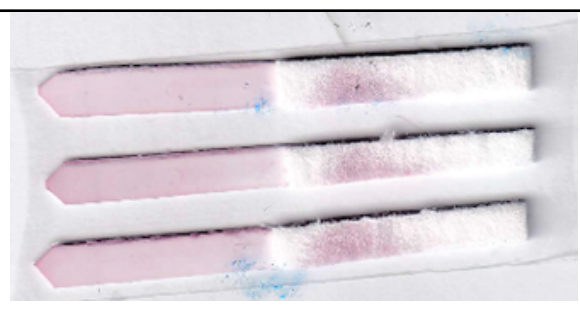	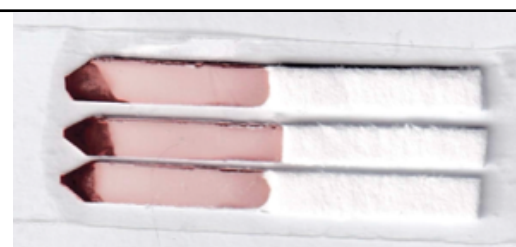
65%	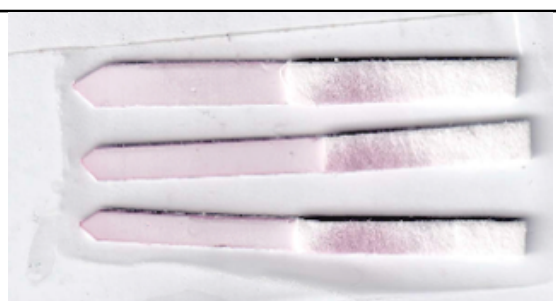	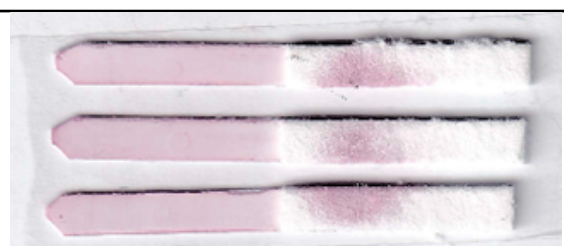	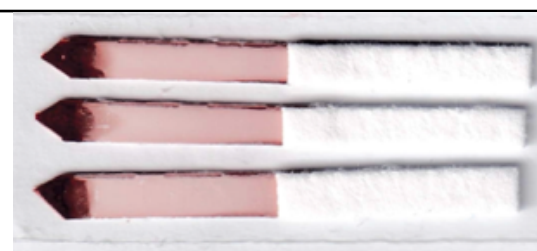

Table S13. – Scans of flow-through strips using Gal-PHEA$_{72}$@AuNP$_{16}$, in doped buffers (varying concentrations of dopant saliva, urine and blood), with a 0.2 mg/mL SBA test line

Percentage %	Saliva	Urine	Blood
0%	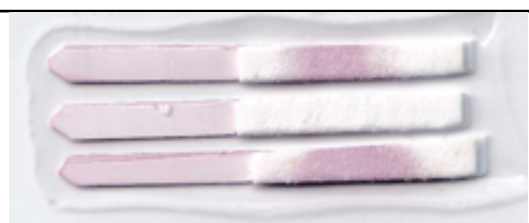	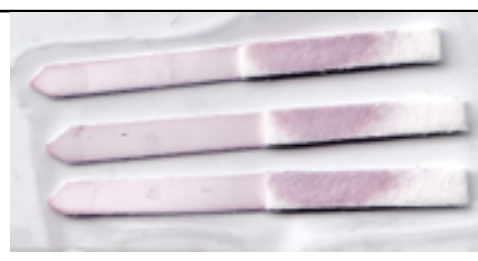	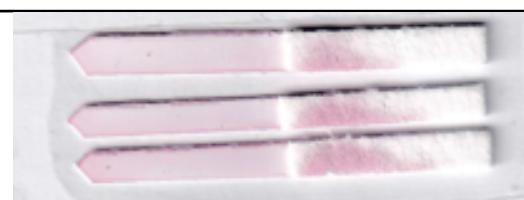
10%	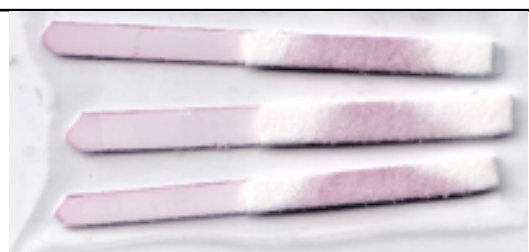	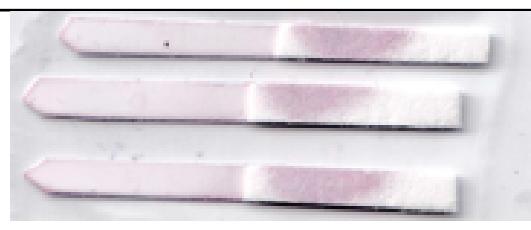	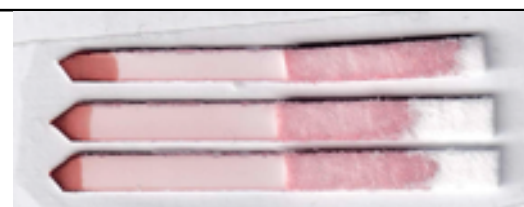
20%	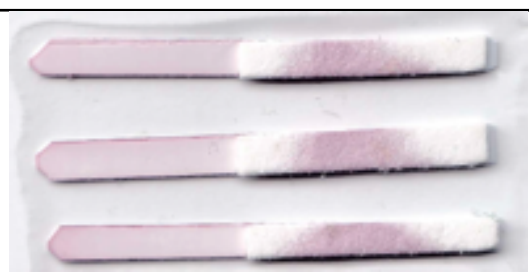	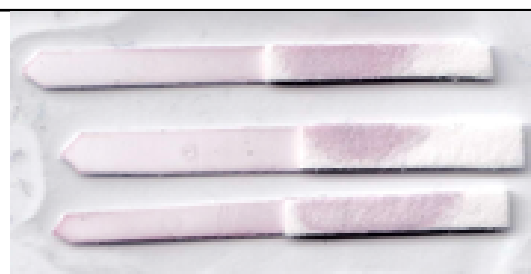	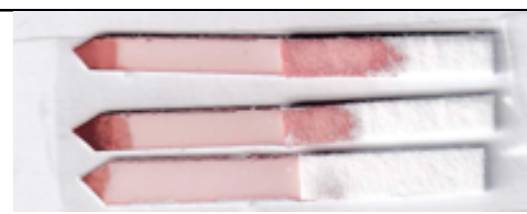
40%	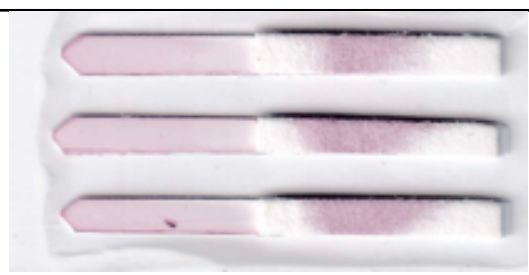	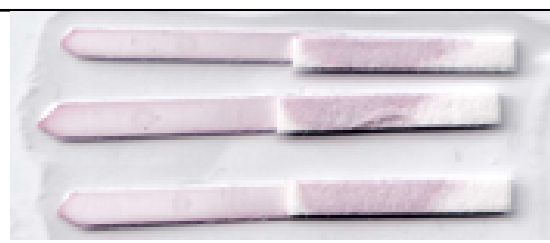	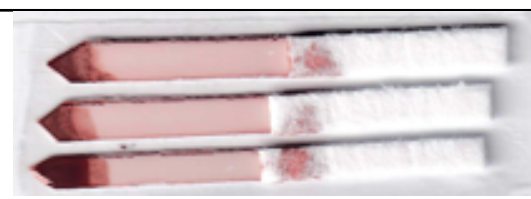
60%	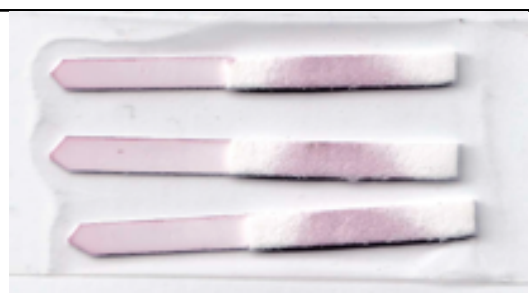	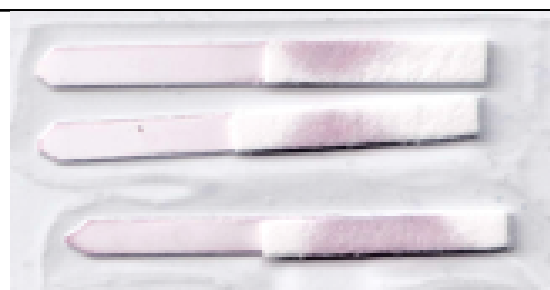	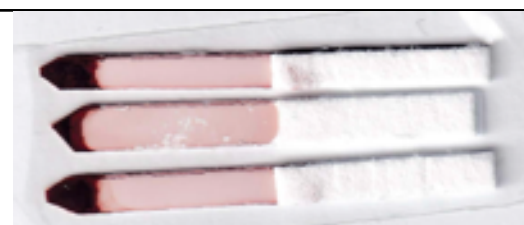
65%	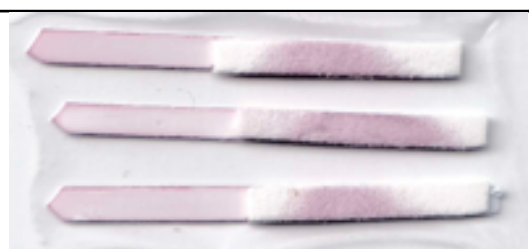	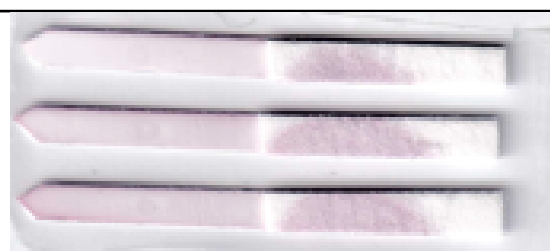	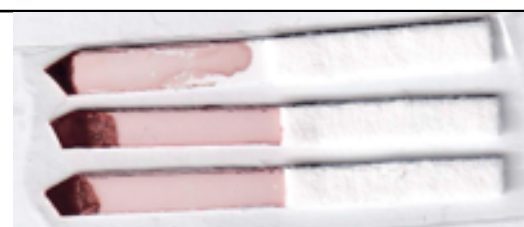

Table S14. – Scans of flow-through strips using Gal-PHEA$_{72}$@AuNP$_{16}$, in doped buffers (varying concentrations of dopant saliva, urine and blood), with a 0.1 mg/mL SBA test line

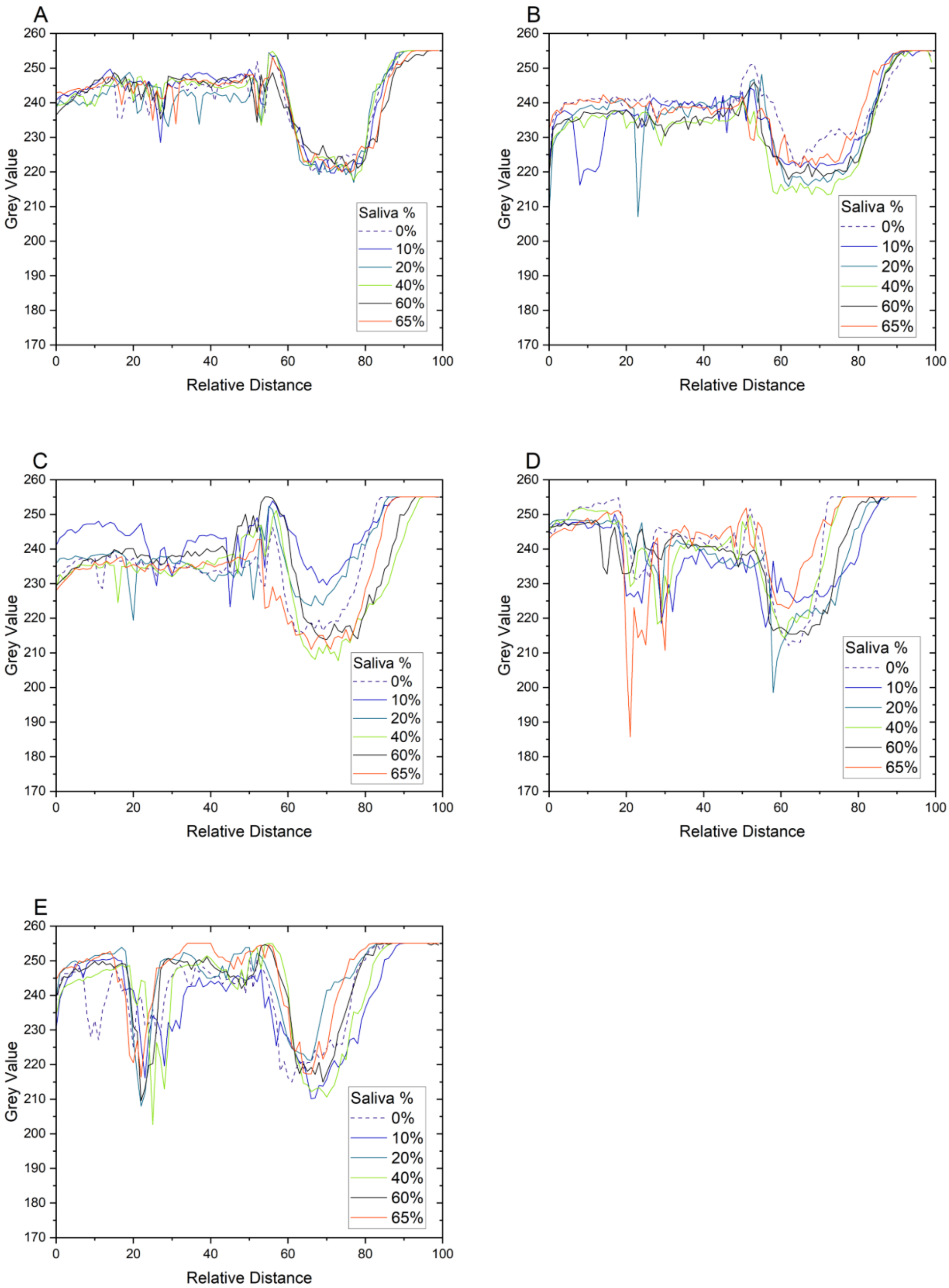
A
B
C
D
E
Grey Value
Relative Distance
Saliva %
0%
10%
20%
40%
60%
65%

Figure S8. – Analysis of scanned flow-through strips using Gal-PHEA$_{40}$@AuNP$_{16}$ in saliva doped buffer at varying concentrations, with varying concentrations of SBA A.) 0.1 mg/mL B.) 0.2 mg/mL C.) 0.3 mg/mL D.) 0.4 mg/mL E.) 0.5 mg/mL.

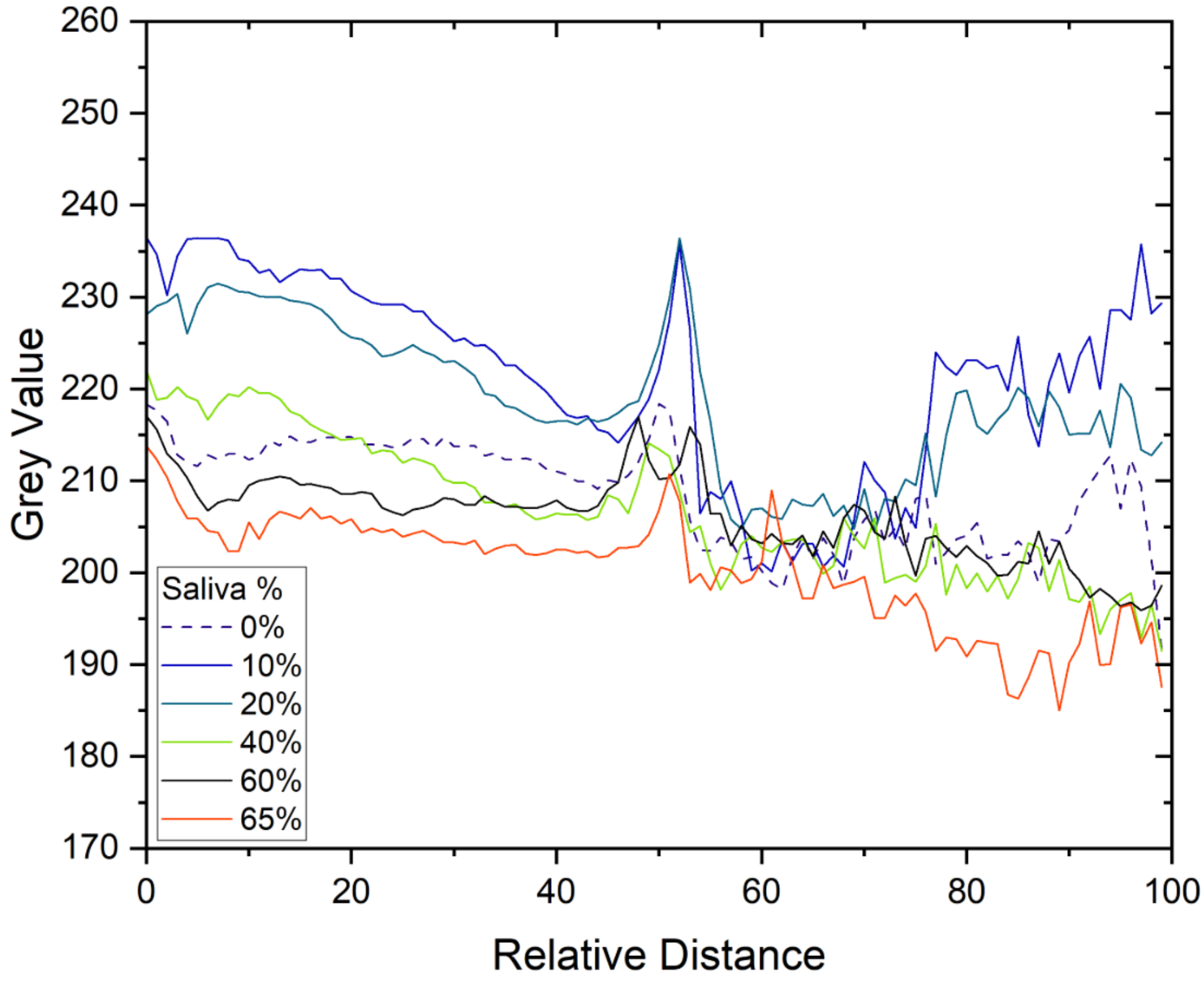

Figure S9. – Analysis of scanned flow-through strips using Gal-PHEA$_{40}$@AuNP$_{16}$ in saliva doped buffer at varying concentrations, with concentration of BSA 0.5 mg/mL.

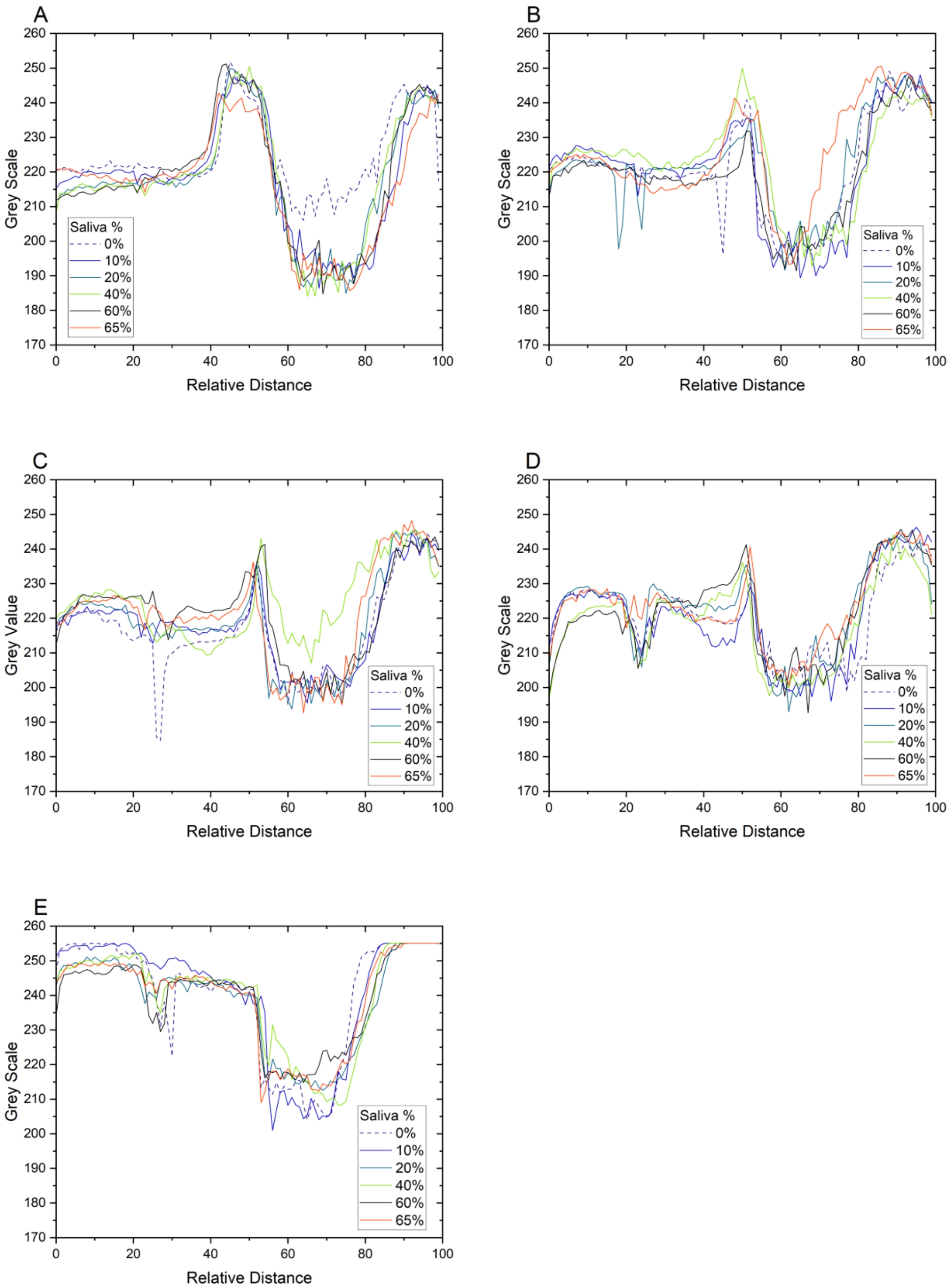
A
Grey Scale
Relative Distance
Saliva %
0%
10%
20%
40%
60%
65%
B
Grey Scale
Relative Distance
Saliva %
0%
10%
20%
40%
60%
65%
C
Grey Value
Relative Distance
Saliva %
0%
10%
20%
40%
60%
65%
D
Grey Scale
Relative Distance
Saliva %
0%
10%
20%
40%
60%
65%
E
Grey Scale
Relative Distance
Saliva %
0%
10%
20%
40%
60%
65%

Figure S10. – Analysis of scanned flow-through strips using Gal-PHEA$_{72}$@AuNP$_{16}$ in saliva doped buffer at varying concentrations, with varying concentrations of SBA A.) 0.1 mg/mL B.) 0.2 mg/mL C.) 0.3 mg/mL D.) 0.4 mg/mL E.) 0.5 mg/mL.

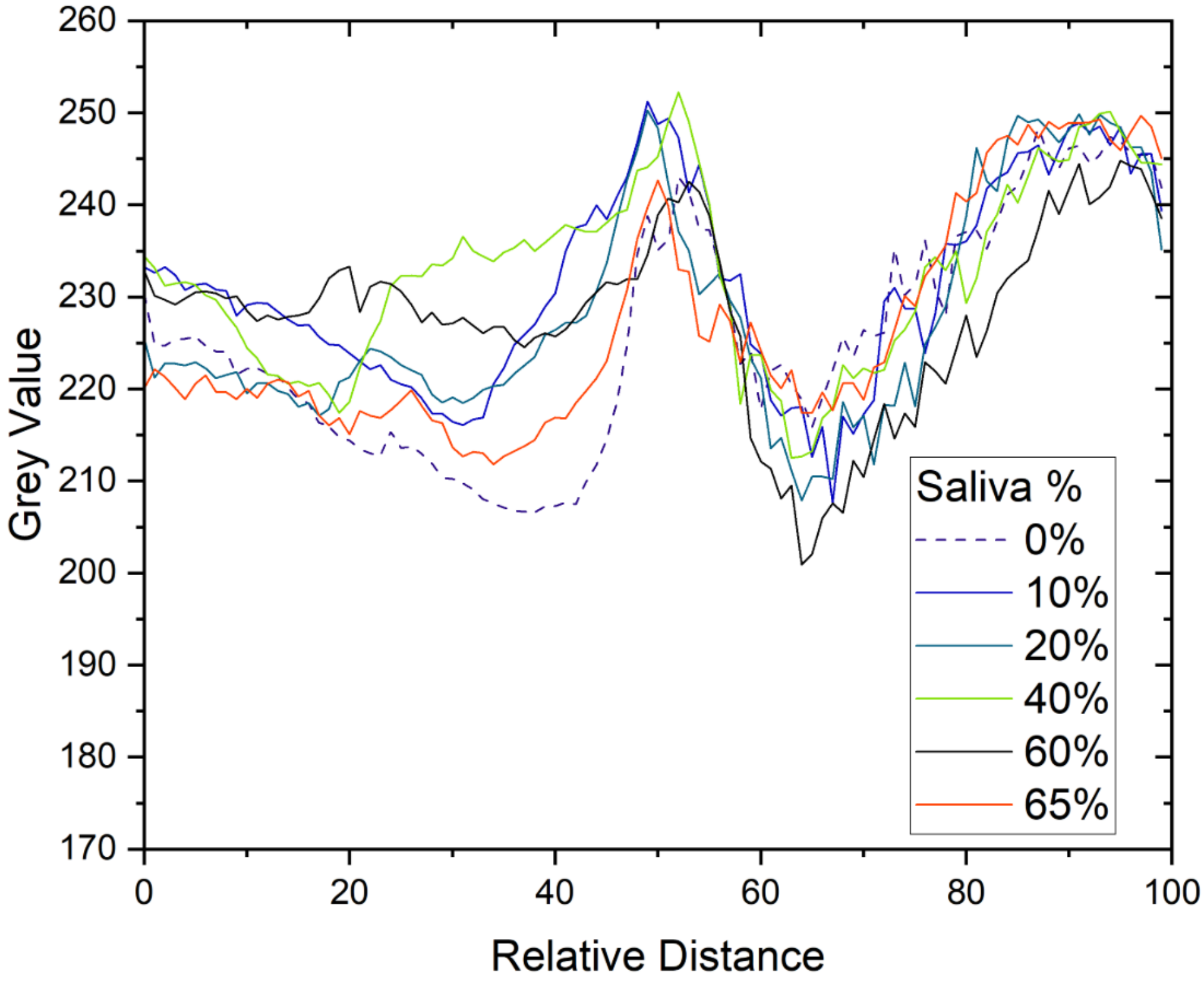

Figure S11. – Analysis of scanned flow-through strips using Gal-PHEA$_{72}$@AuNP$_{16}$ in saliva doped buffer at varying concentrations, with concentration of BSA 0.5 mg/mL.

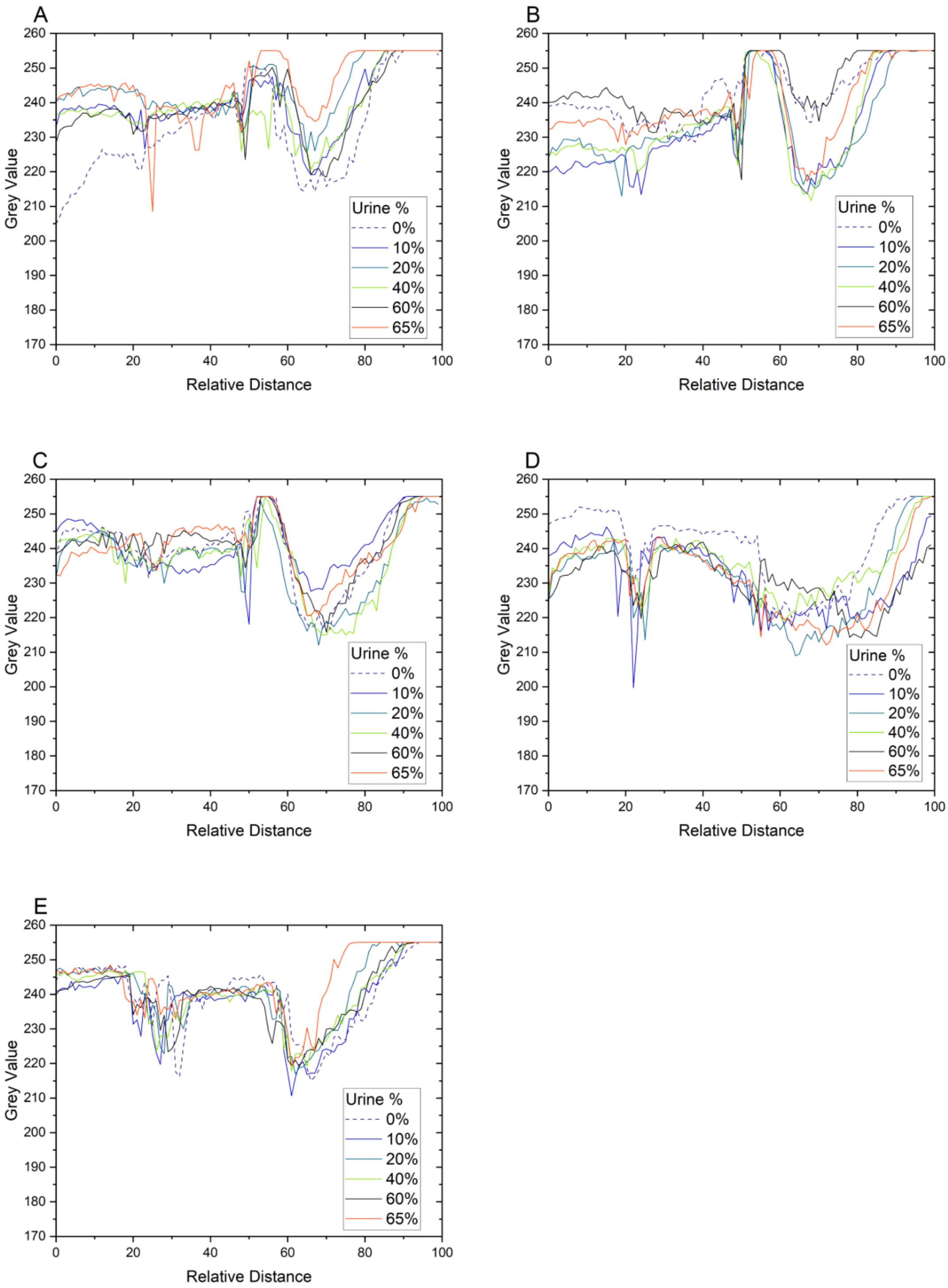
A
Grey Value
Relative Distance
Urine %
0%
10%
20%
40%
60%
65%
B
Grey Value
Relative Distance
Urine %
0%
10%
20%
40%
60%
65%
C
Grey Value
Relative Distance
Urine %
0%
10%
20%
40%
60%
65%
D
Grey Value
Relative Distance
Urine %
0%
10%
20%
40%
60%
65%
E
Grey Value
Relative Distance
Urine %
0%
10%
20%
40%
60%
65%

Figure S12. – Analysis of scanned flow-through strips using Gal-PHEA$_{40}$@AuNP$_{16}$ in urine doped buffer at varying concentrations, with varying concentrations of SBA A.) 0.1 mg/mL B.) 0.2 mg/mL C.) 0.3 mg/mL D.) 0.4 mg/mL E.) 0.5 mg/mL.

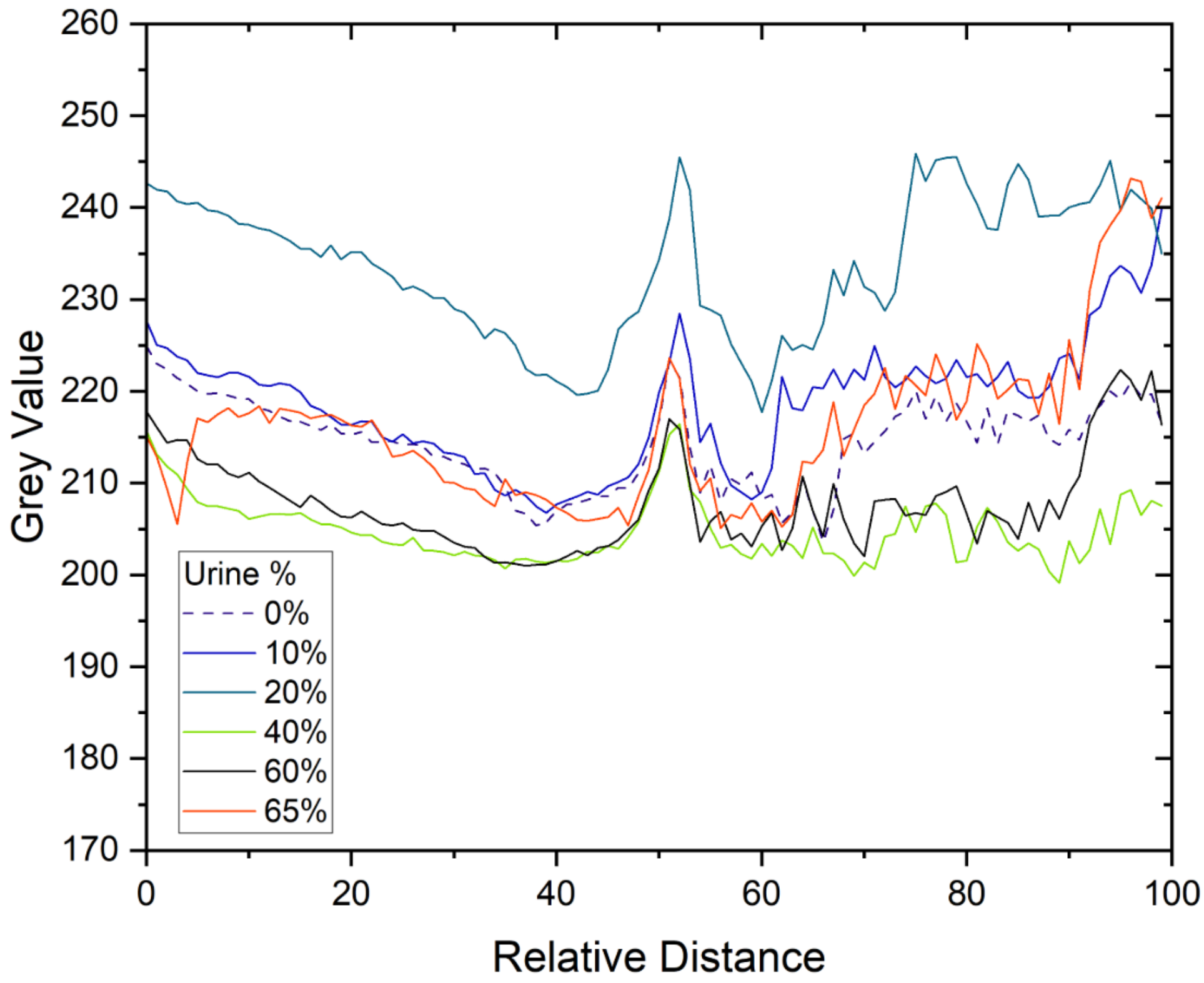

Figure S13. – Analysis of scanned flow-through strips using Gal-PHEA$_{40}$@AuNP$_{16}$ in urine doped buffer at varying concentrations, with concentration of BSA 0.5 mg/mL.

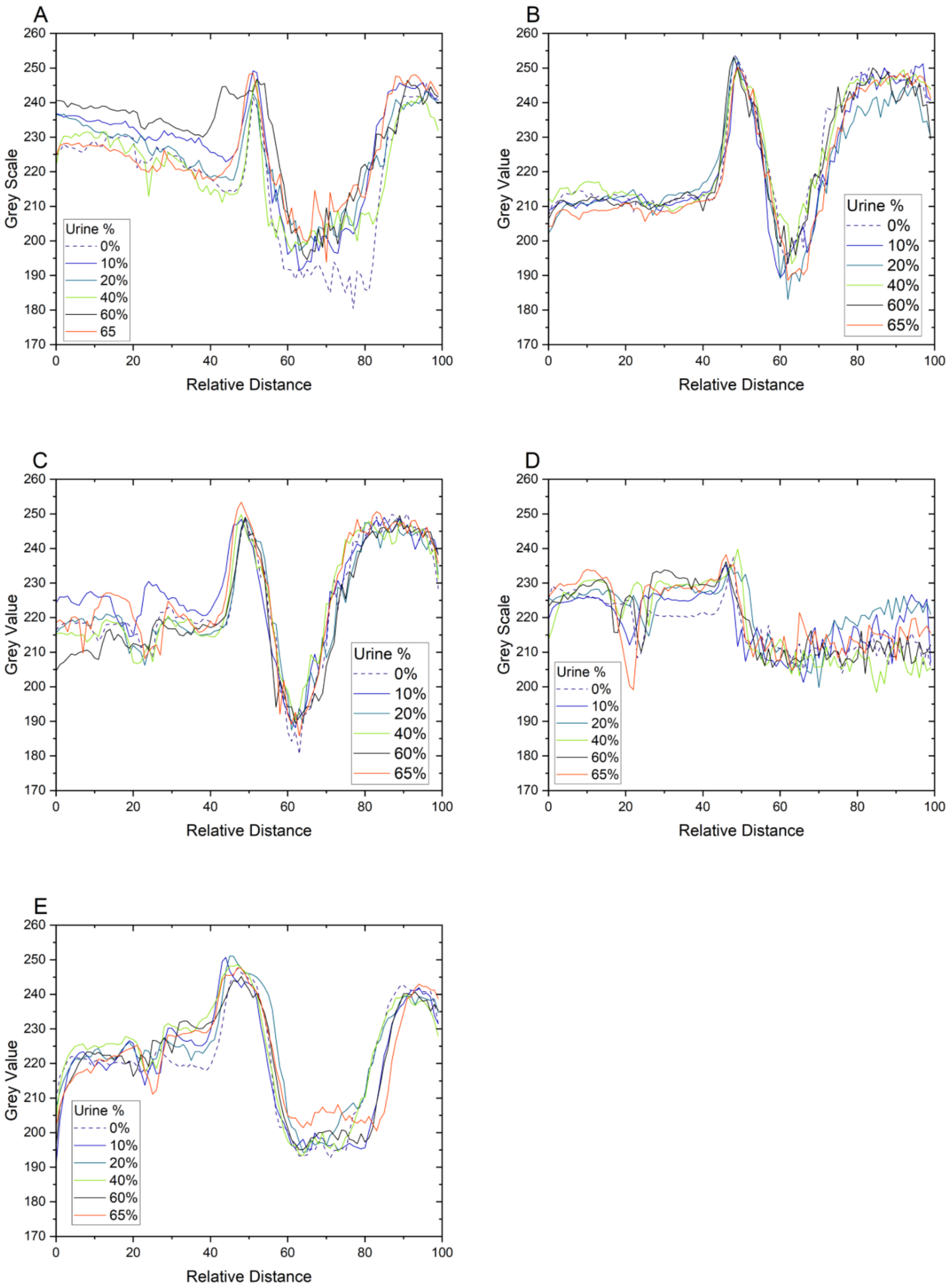

Figure S14. – Analysis of scanned flow-through strips using Gal-PHEA$_{72}$@AuNP$_{16}$ in urine doped buffer at varying concentrations, with varying concentrations of SBA A.) 0.1 mg/mL B.) 0.2 mg/mL C.) 0.3 mg/mL D.) 0.4 mg/mL E.) 0.5 mg/mL.

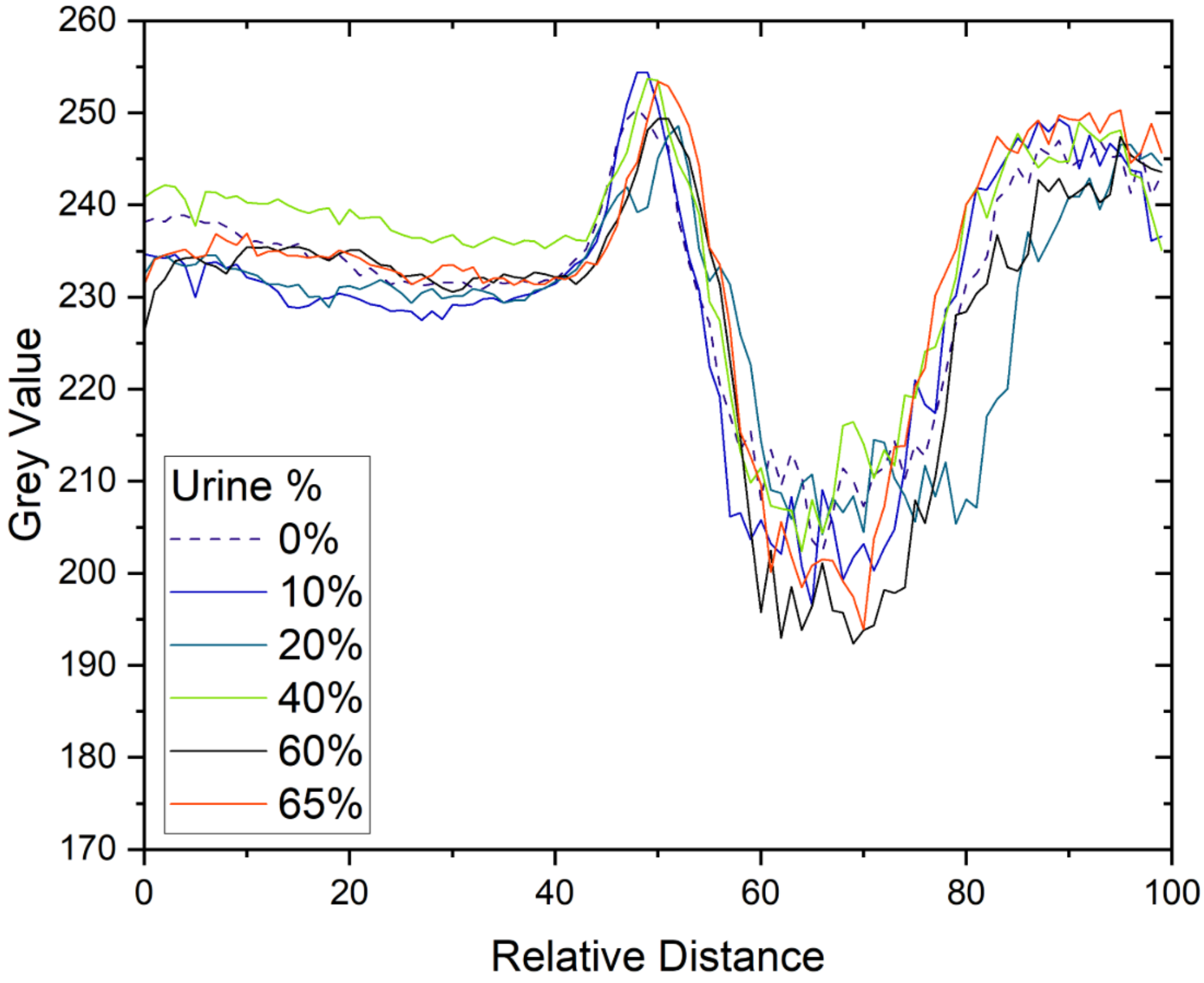

Figure S15. – Analysis of scanned flow-through strips using Gal-PHEA$_{72}$@AuNP$_{16}$ in urine doped buffer at varying concentrations, with concentration of BSA 0.5 mg/mL.

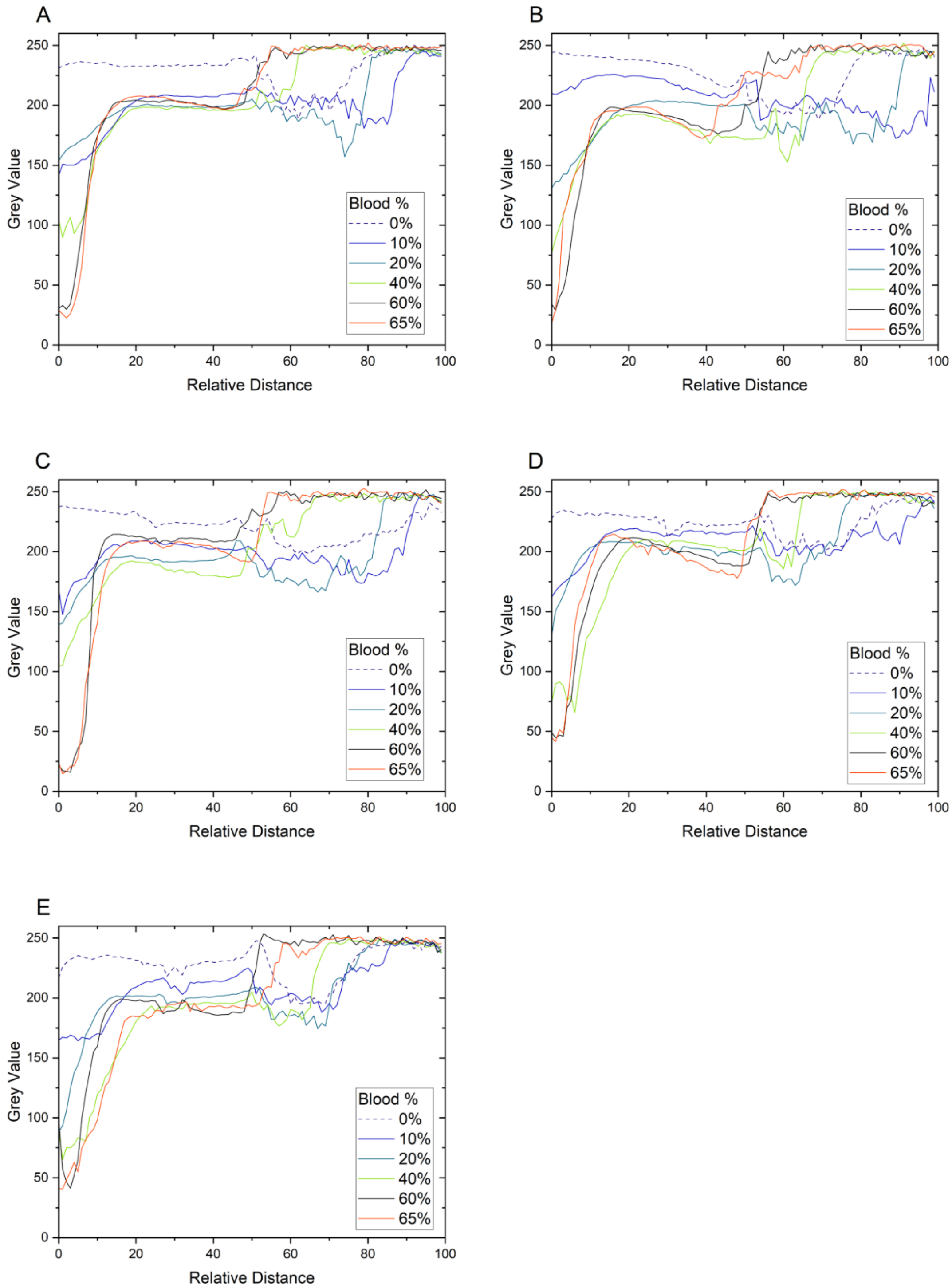
A
B
C
D
E
Grey Value
Relative Distance
Blood %
0%
10%
20%
40%
60%
65%

Figure S16. – Analysis of scanned flow-through strips using Gal-PHEA$_{40}$@AuNP$_{16}$ in blood doped buffer at varying concentrations, with varying concentrations of SBA A.) 0.1 mg/mL B.) 0.2 mg/mL C.) 0.3 mg/mL D.) 0.4 mg/mL E.) 0.5 mg/mL.

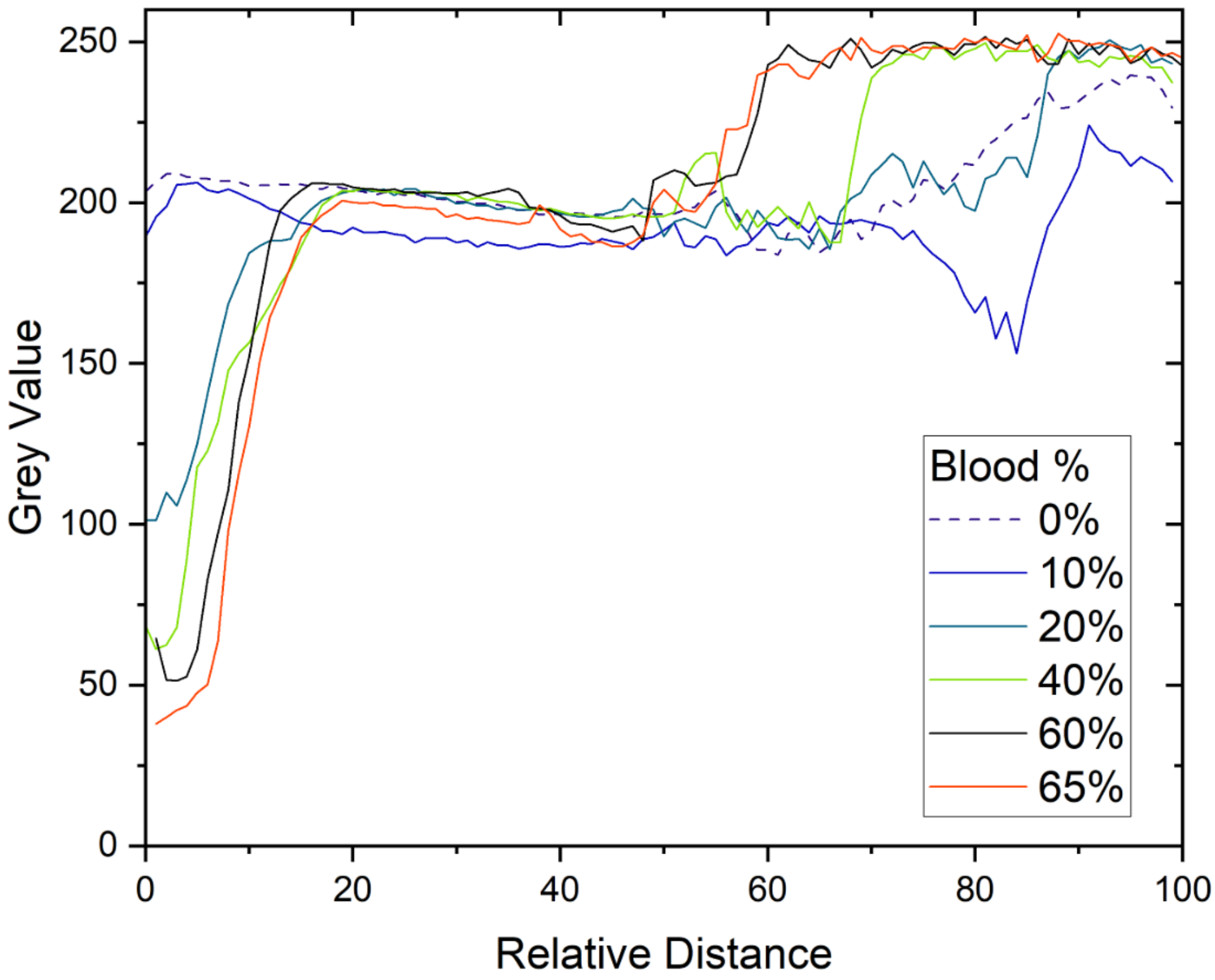

Figure S17. – Analysis of scanned flow-through strips using Gal-PHEA$_{40}$@AuNP$_{16}$ in blood doped buffer at varying concentrations, with concentration of BSA 0.5 mg/mL.

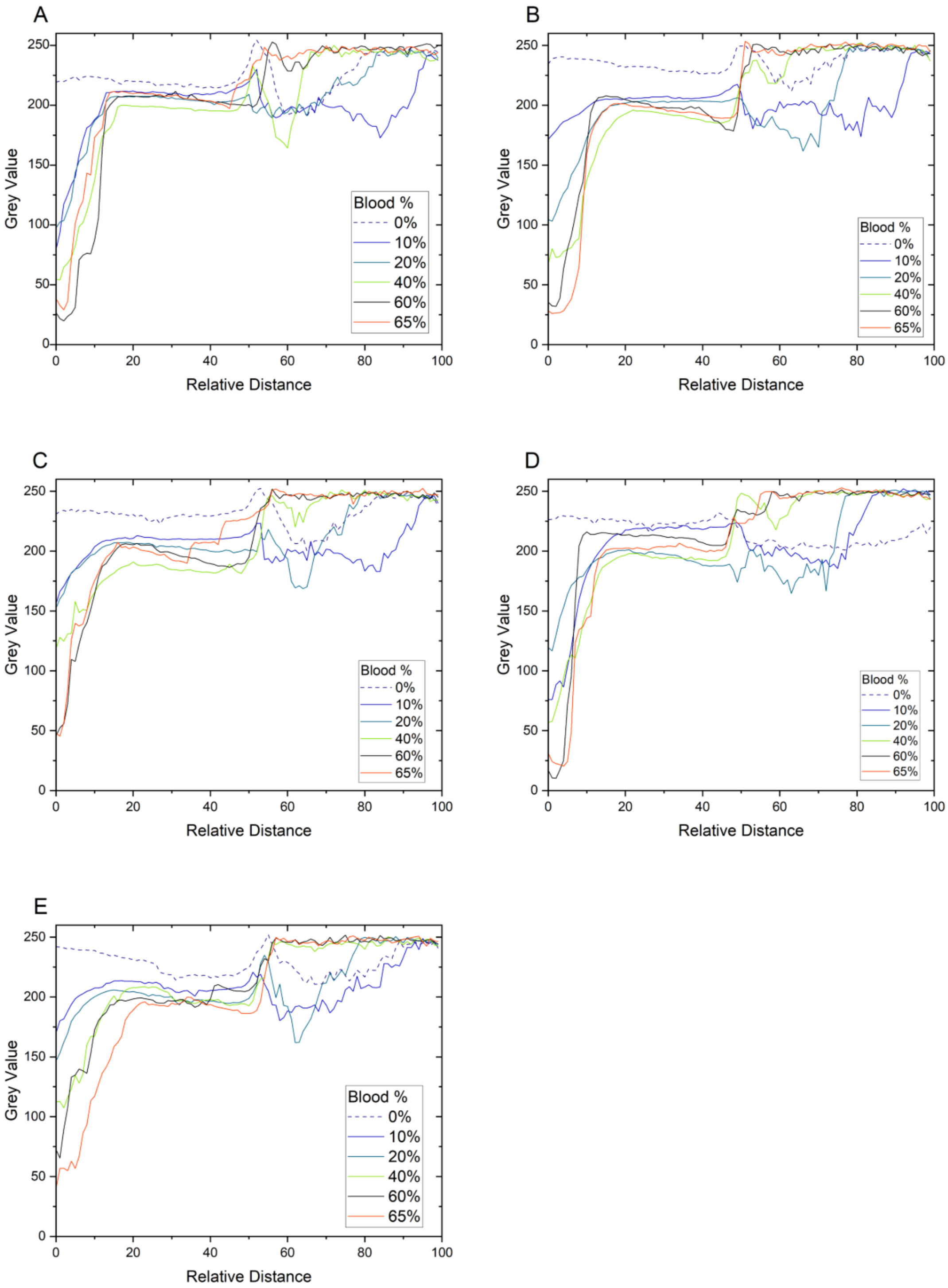
A
B
C
D
E
Grey Value
Relative Distance
0
50
100
150
200
250
0
20
40
60
80
100
Blood %
0%
10%
20%
40%
60%
65%

Figure S18. – Analysis of scanned flow-through strips using Gal-PHEA$_{72}$@AuNP$_{16}$ in blood doped buffer at varying concentrations, with varying concentrations of SBA A.) 0.1 mg/mL B.) 0.2 mg/mL C.) 0.3 mg/mL D.) 0.4 mg/mL E.) 0.5 mg/mL.

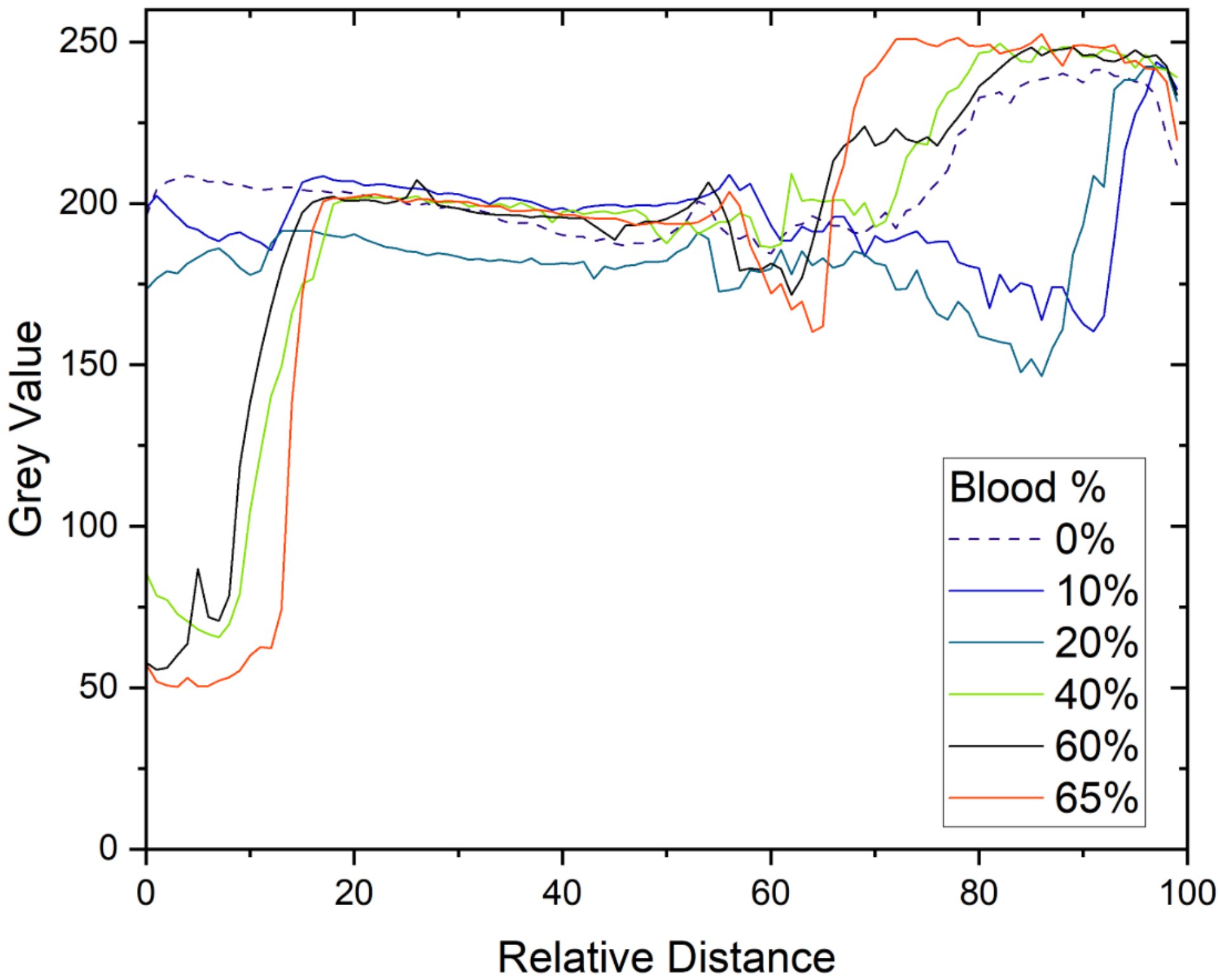

Figure S19. – Analysis of scanned flow-through strips using Gal-PHEA$_{72}$@AuNP$_{16}$ in blood doped buffer at varying concentrations, with concentration of BSA 0.5 mg/mL.

Signal to Noise Results for Flow-Through

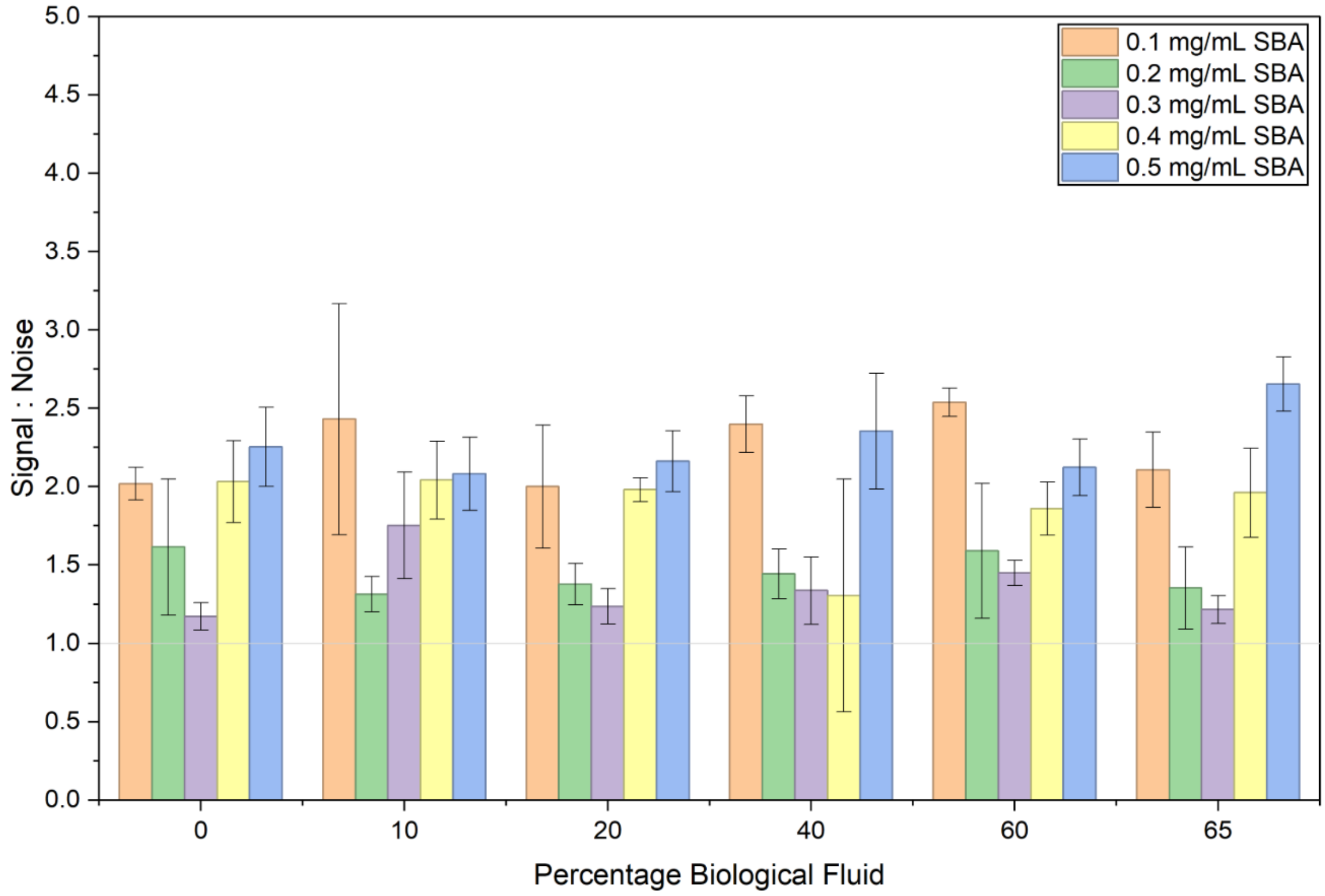

Figure S20. – Signal to noise analysis of Gal-$PHEA_{40}$@$AuNP_{16}$, with saliva doped buffer at varying percentages, and varying SBA concentrations of 0.1 mg/mL to 0.5 mg/mL.

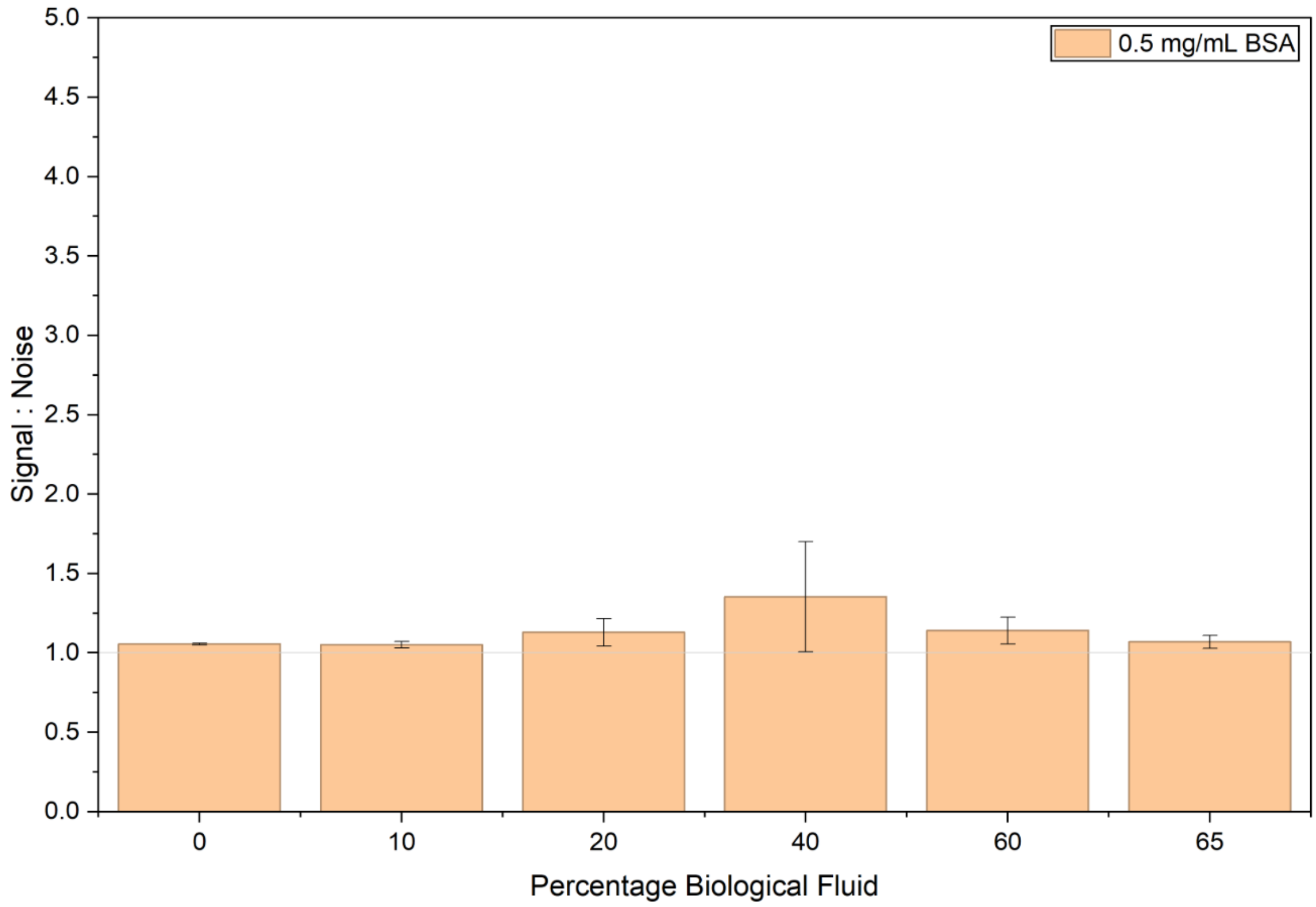

Figure S21. – Signal to noise analysis of Gal-PHEA$_{40}$@AuNP$_{16}$, with saliva doped buffer at varying percentages, and BSA concentration of 0.5 mg/mL.

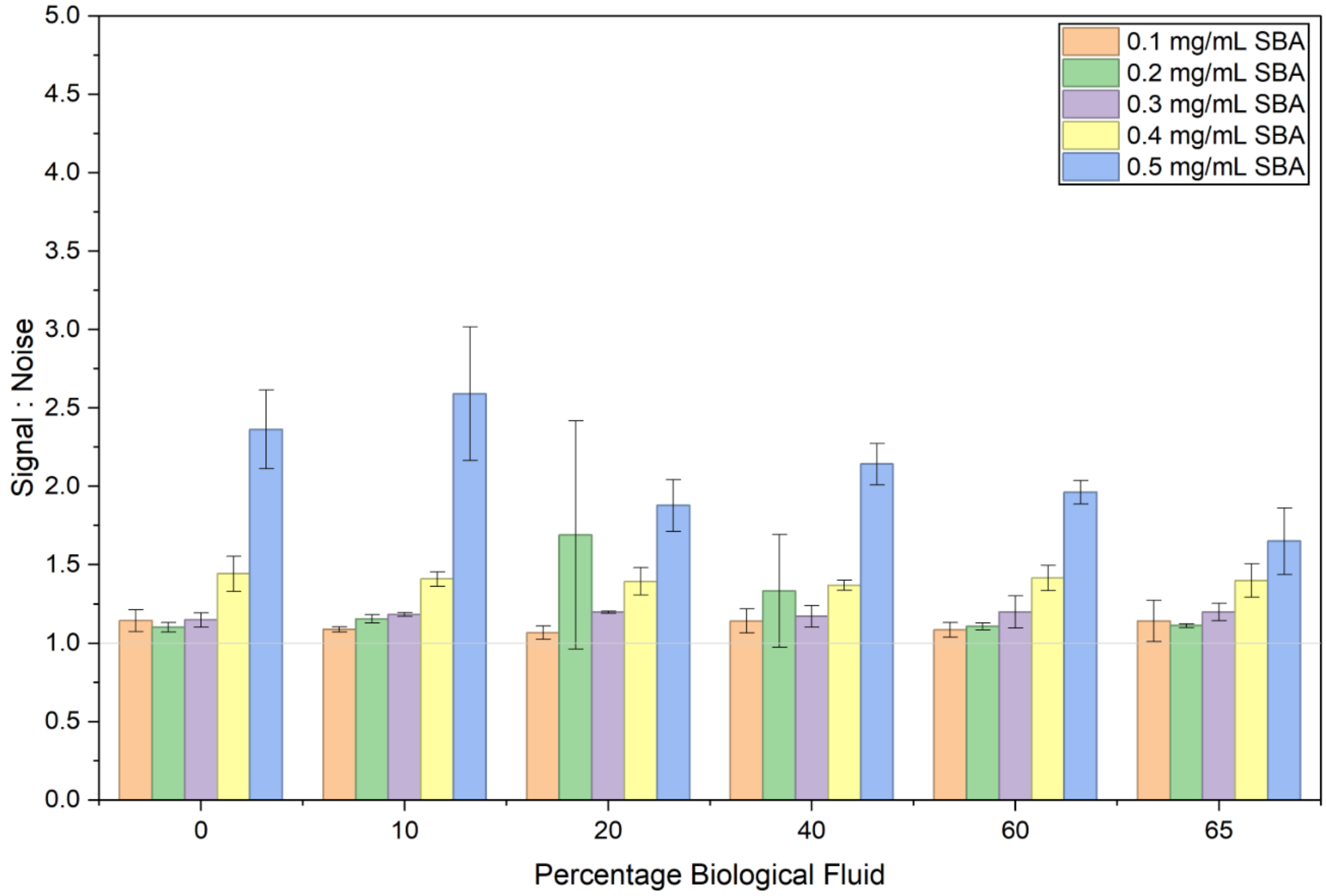

Figure S22. – Signal to noise analysis of Gal-PHEA$_{72}$@AuNP$_{16}$, with saliva doped buffer at varying percentages, and varying SBA concentrations of 0.1 mg/mL to 0.5 mg/mL.

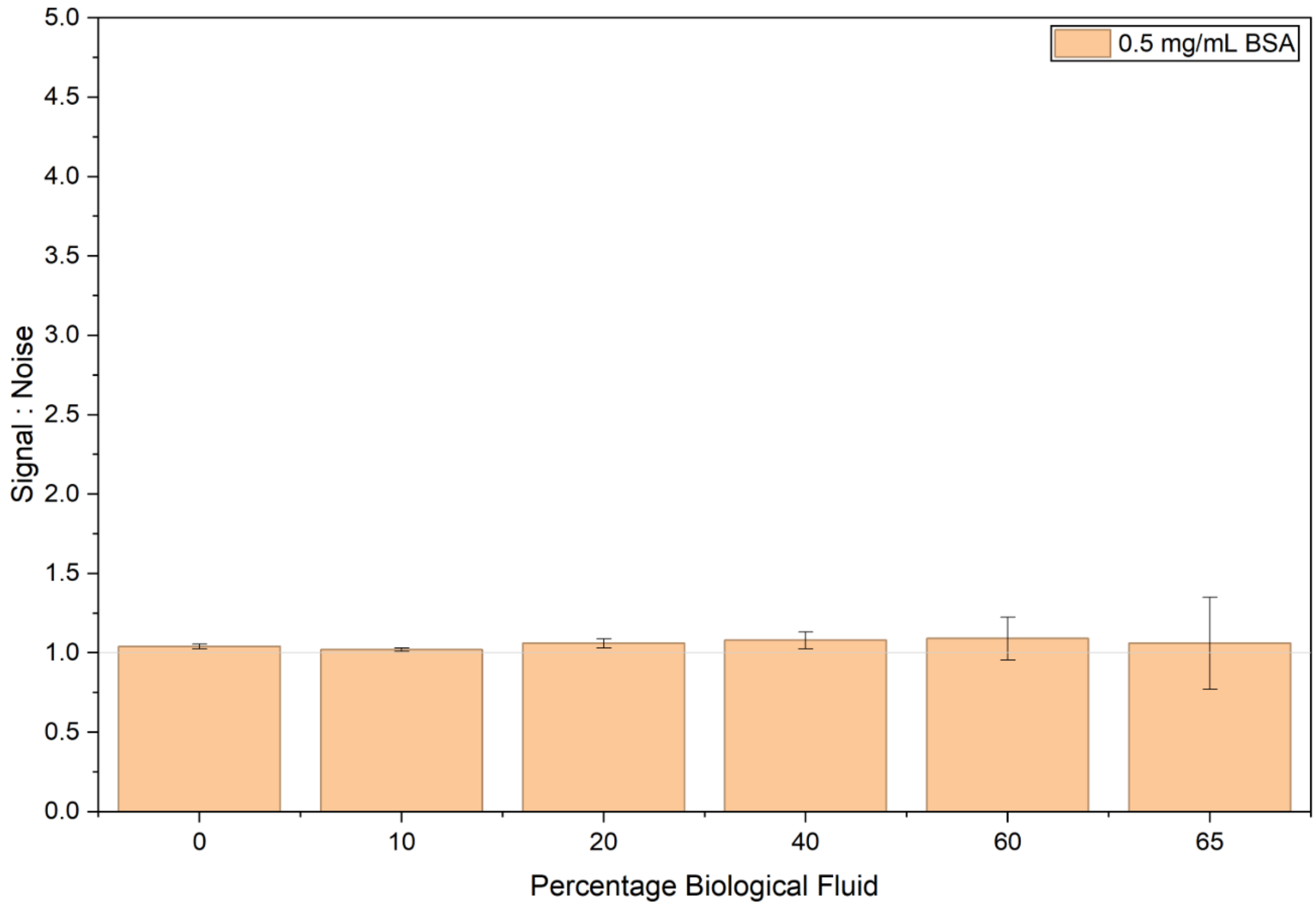

Figure S23. – Signal to noise analysis of Gal-PHEA$_{72}$@AuNP$_{16}$, with saliva doped buffer at varying percentages, and BSA concentration of 0.5 mg/mL.

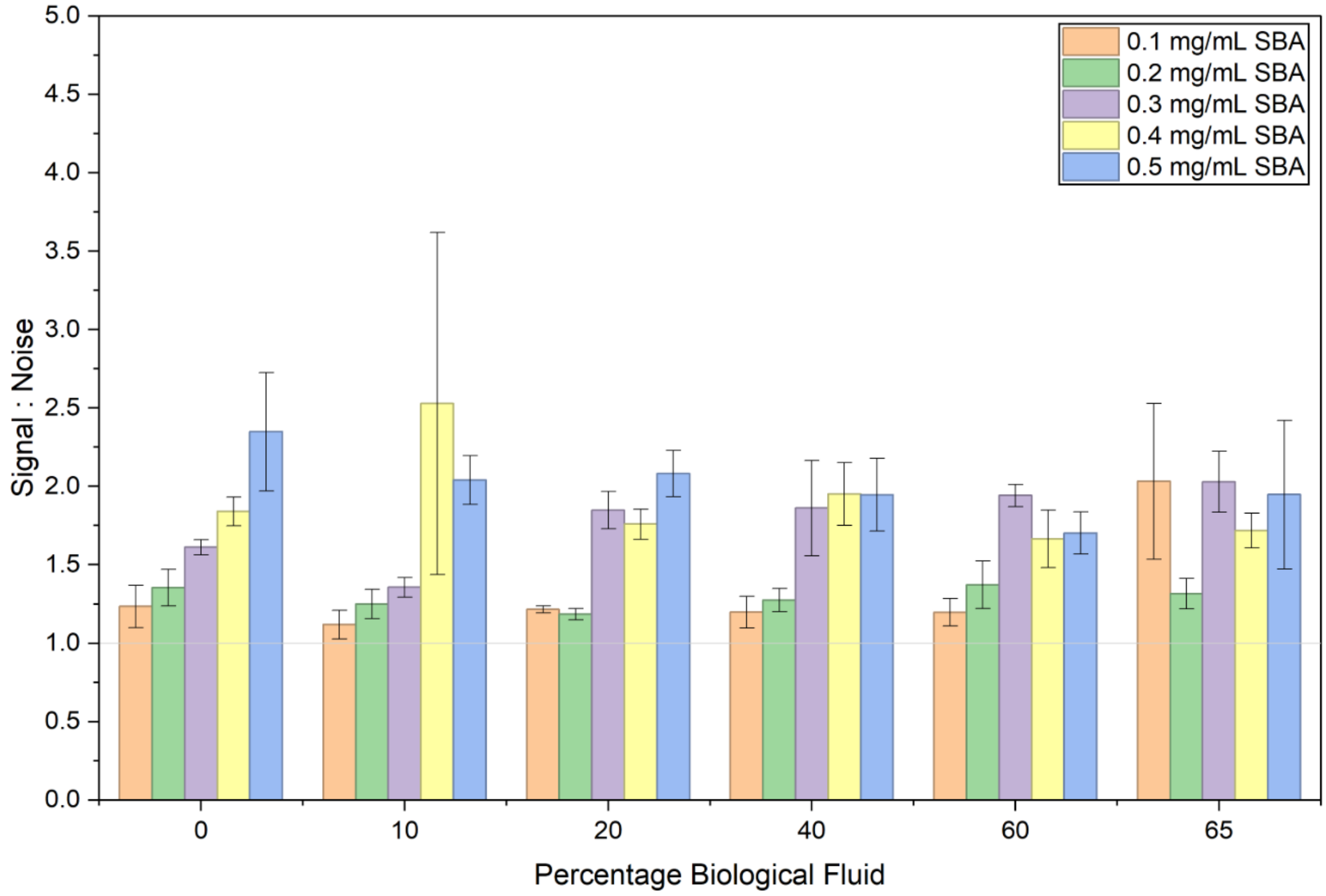

Figure S24. – Signal to noise analysis of Gal-PHEA$_{40}$@AuNP$_{16}$, with urine doped buffer at varying percentages, and varying SBA concentrations of 0.1 mg/mL to 0.5 mg/mL.

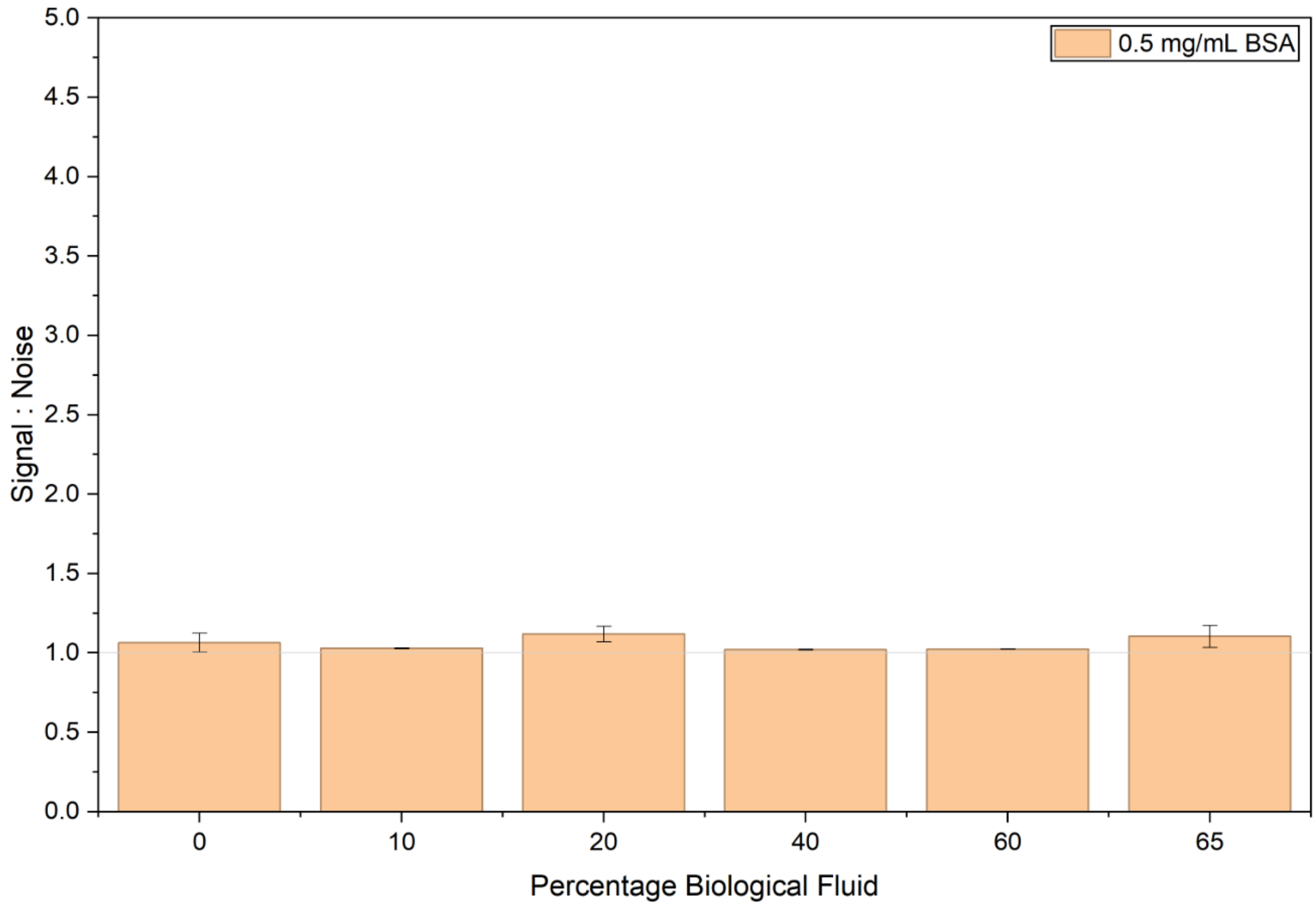

Figure S25. – Signal to noise analysis of Gal-PHEA$_{40}$@AuNP$_{16}$, with urine doped buffer at varying percentages, and BSA concentration of 0.5 mg/mL.

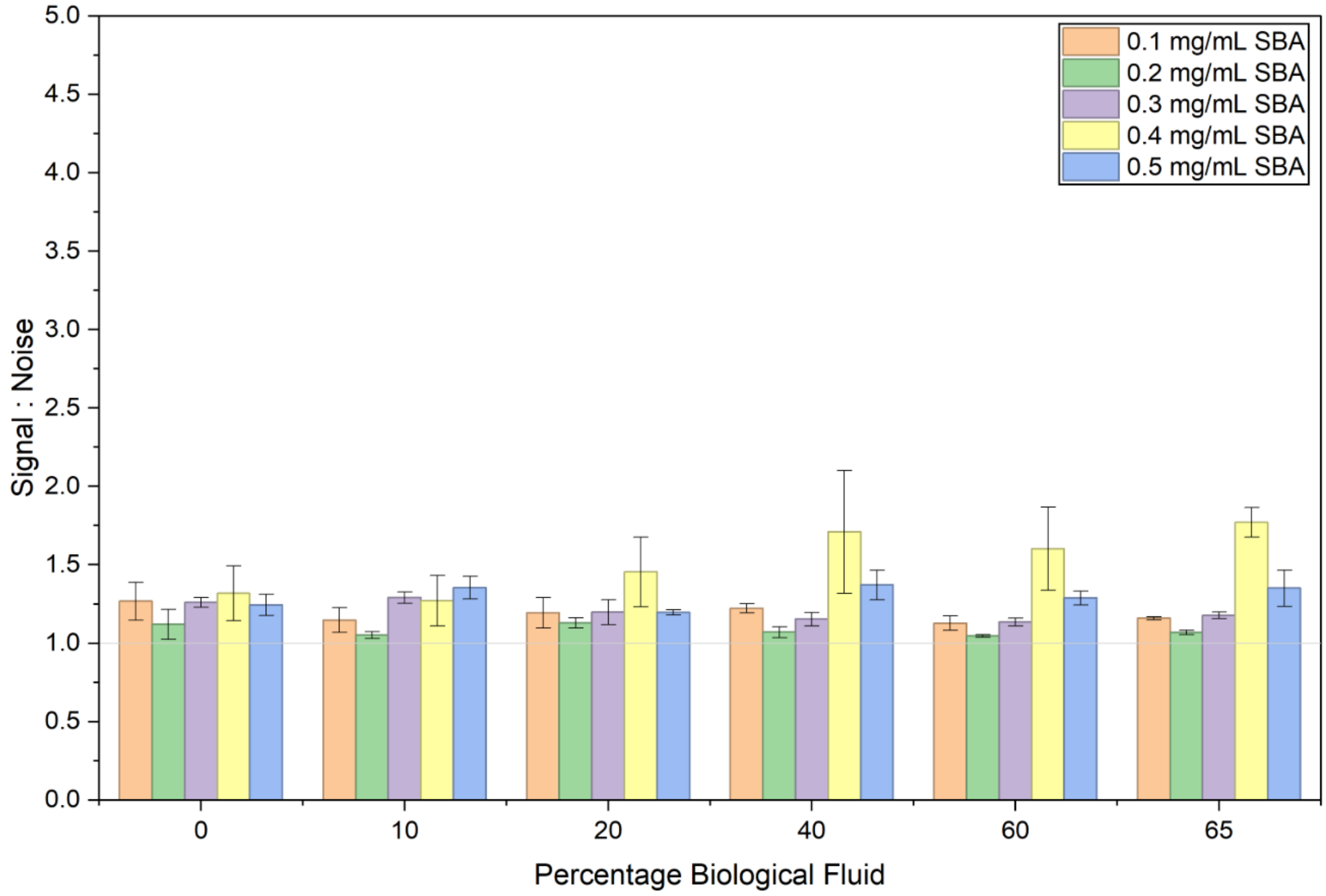

Figure S26. – Signal to noise analysis of Gal-PHEA$_{72}$@AuNP$_{16}$, with urine doped buffer at varying percentages, and varying SBA concentrations of 0.1 mg/mL to 0.5 mg/mL.

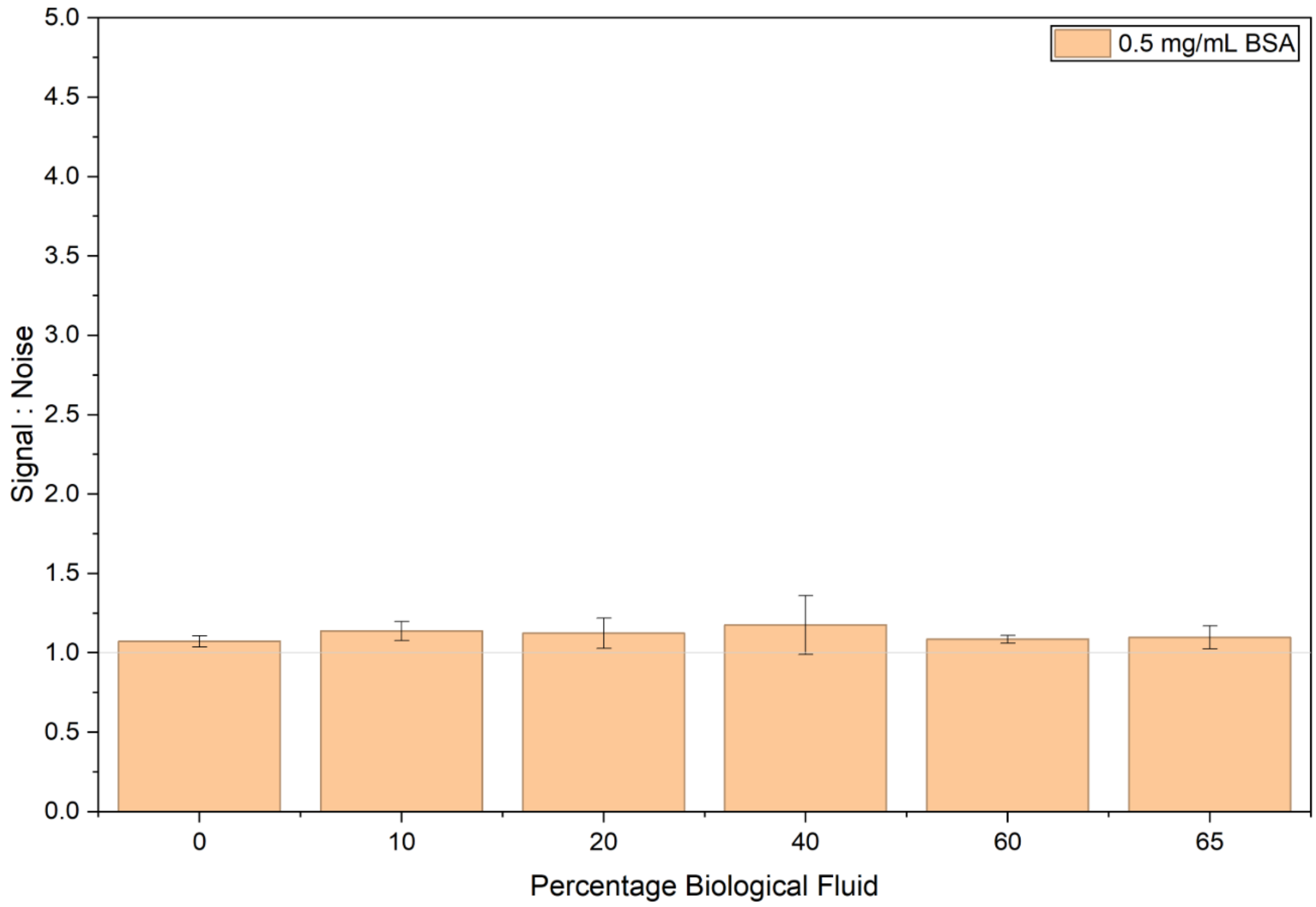

Figure S27. – Signal to noise analysis of Gal-$PHEA_{72}$@$AuNP_{16}$, with urine doped buffer at varying percentages, and BSA concentration of 0.5 mg/mL.

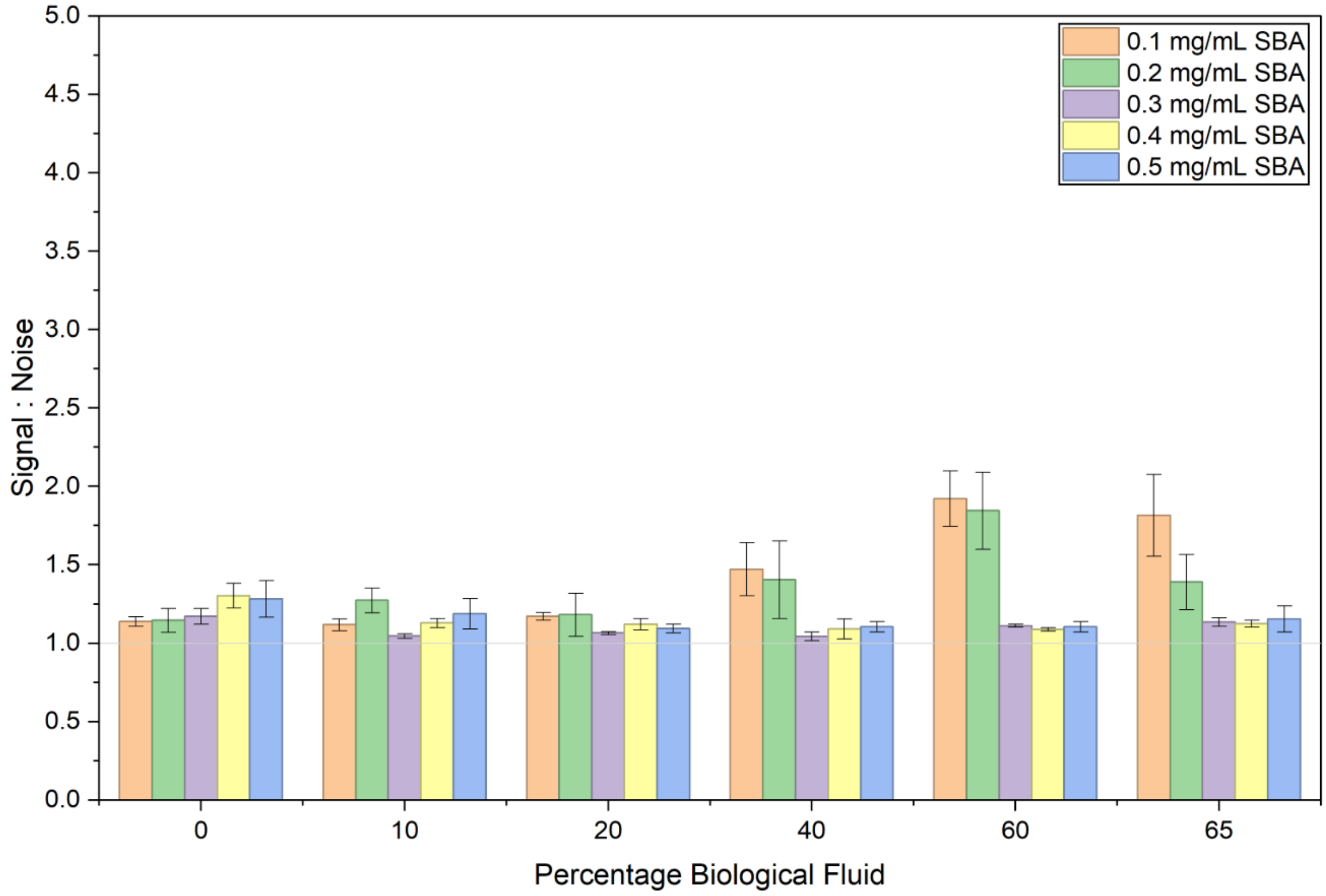

Figure S28. – Signal to noise analysis of Gal-PHEA$_{40}$@AuNP$_{16}$, with blood doped buffer at varying percentages, and varying SBA concentrations of 0.1 mg/mL to 0.5 mg/mL.

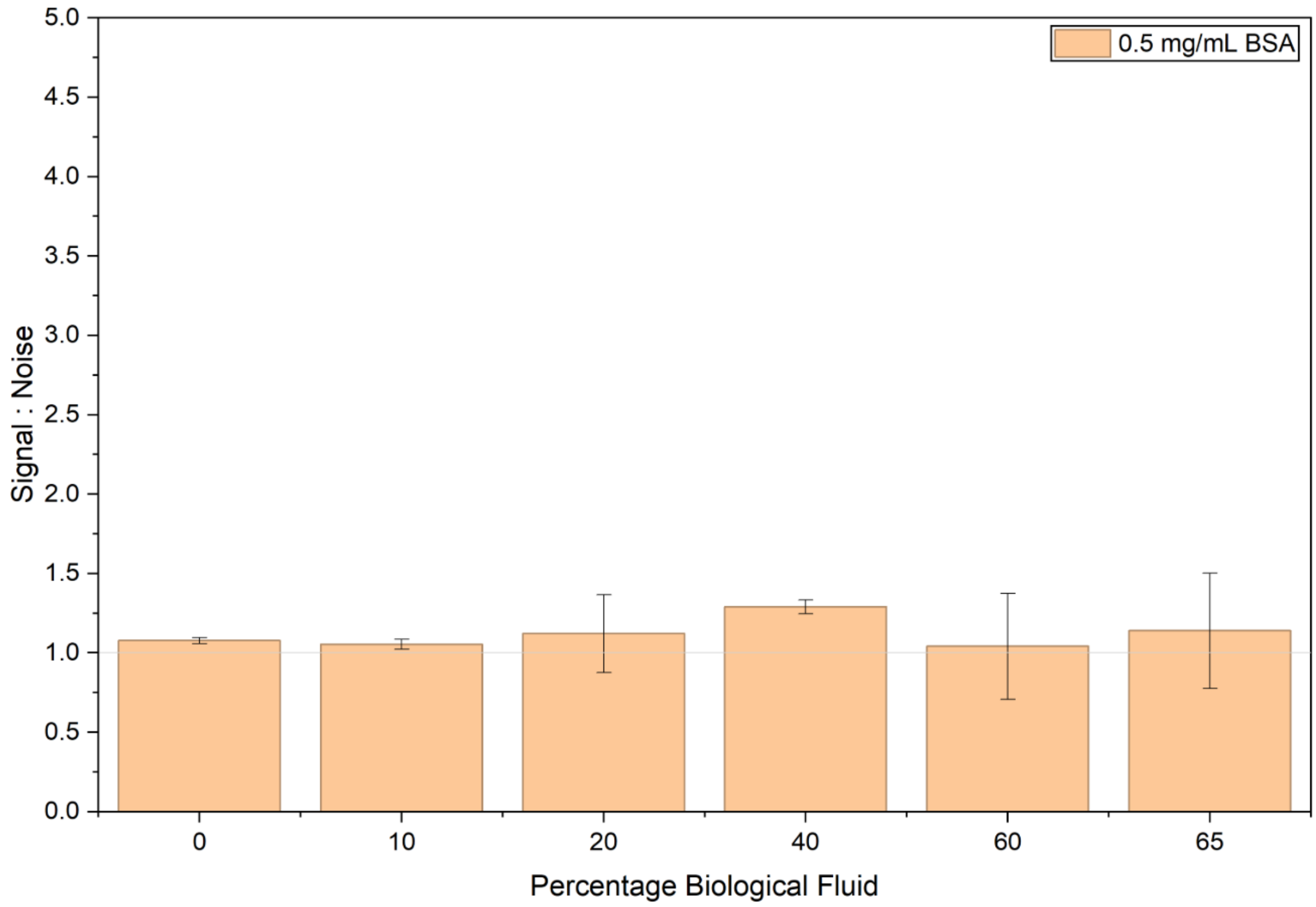

Figure S29. – Signal to noise analysis of Gal-PHEA$_{40}$@AuNP$_{16}$, with blood doped buffer at varying percentages, and BSA concentration of 0.5 mg/mL.

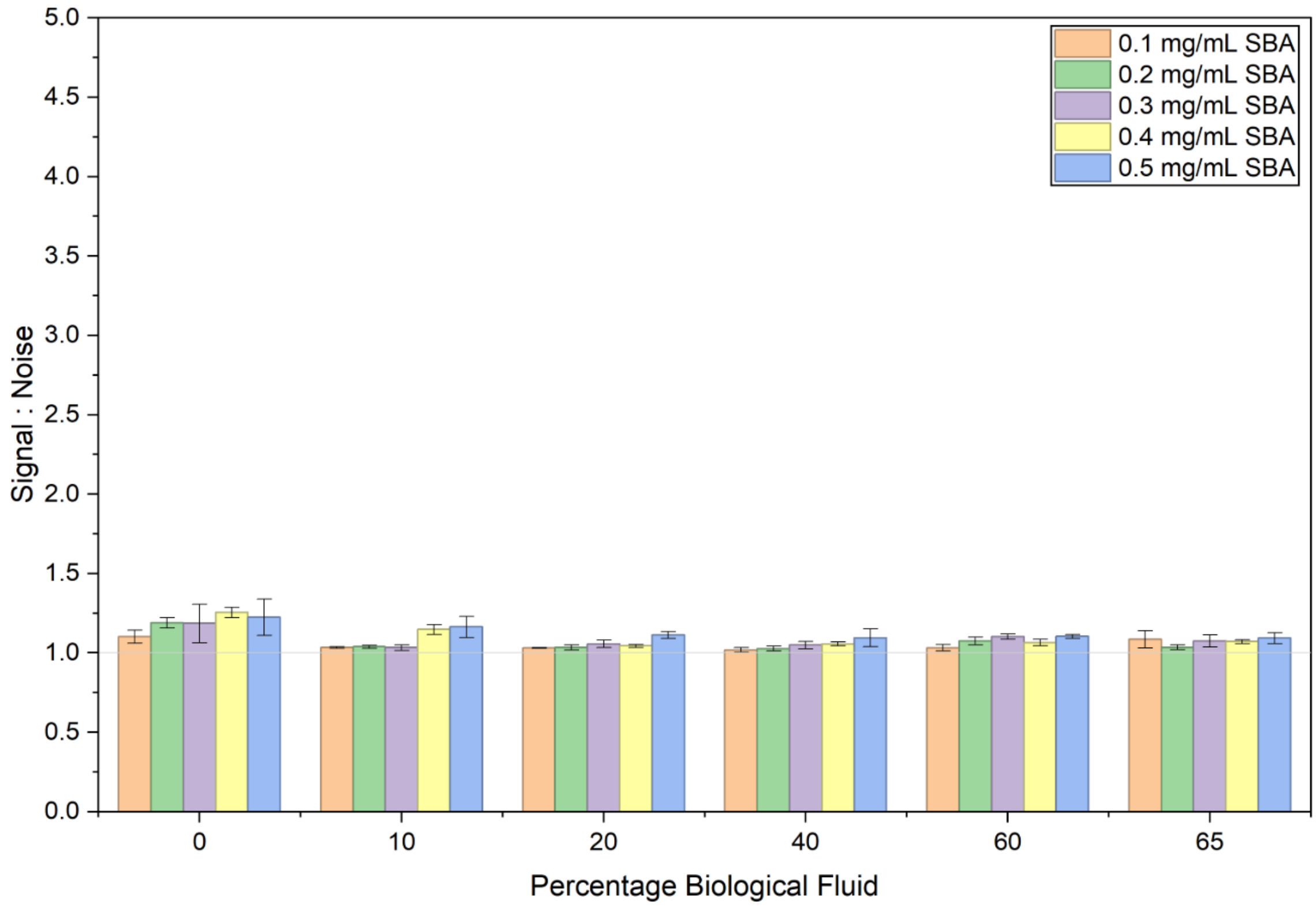

Figure S30. – Signal to noise analysis of Gal-PHEA$_{72}$@AuNP$_{16}$, with blood doped buffer at varying percentages, and varying SBA concentrations of 0.1 mg/mL to 0.5 mg/mL.

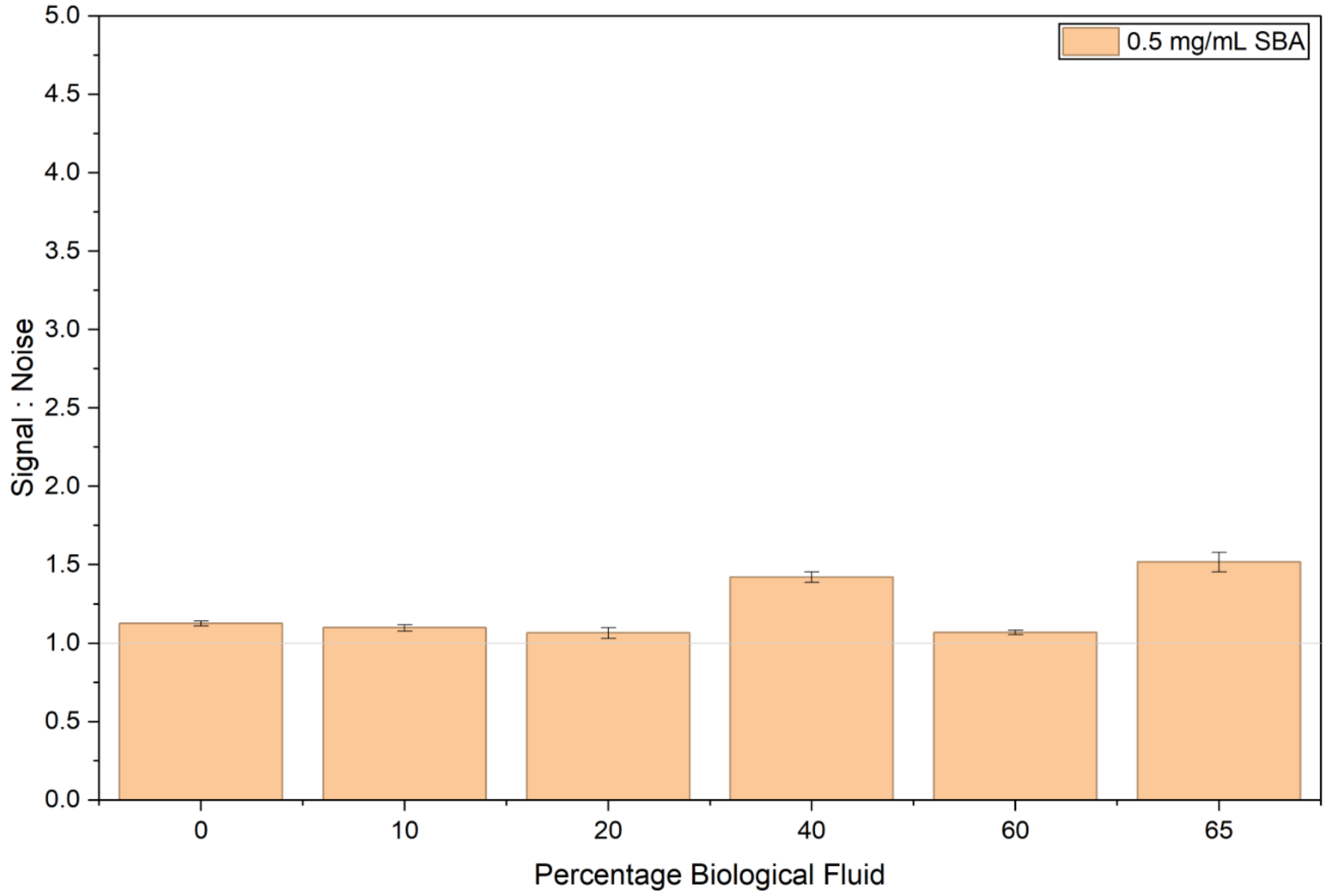

Figure S31. – Signal to noise analysis of Gal-PHEA$_{72}$@AuNP$_{16}$, with blood doped buffer at varying percentages, and BSA concentration of 0.5 mg/mL.

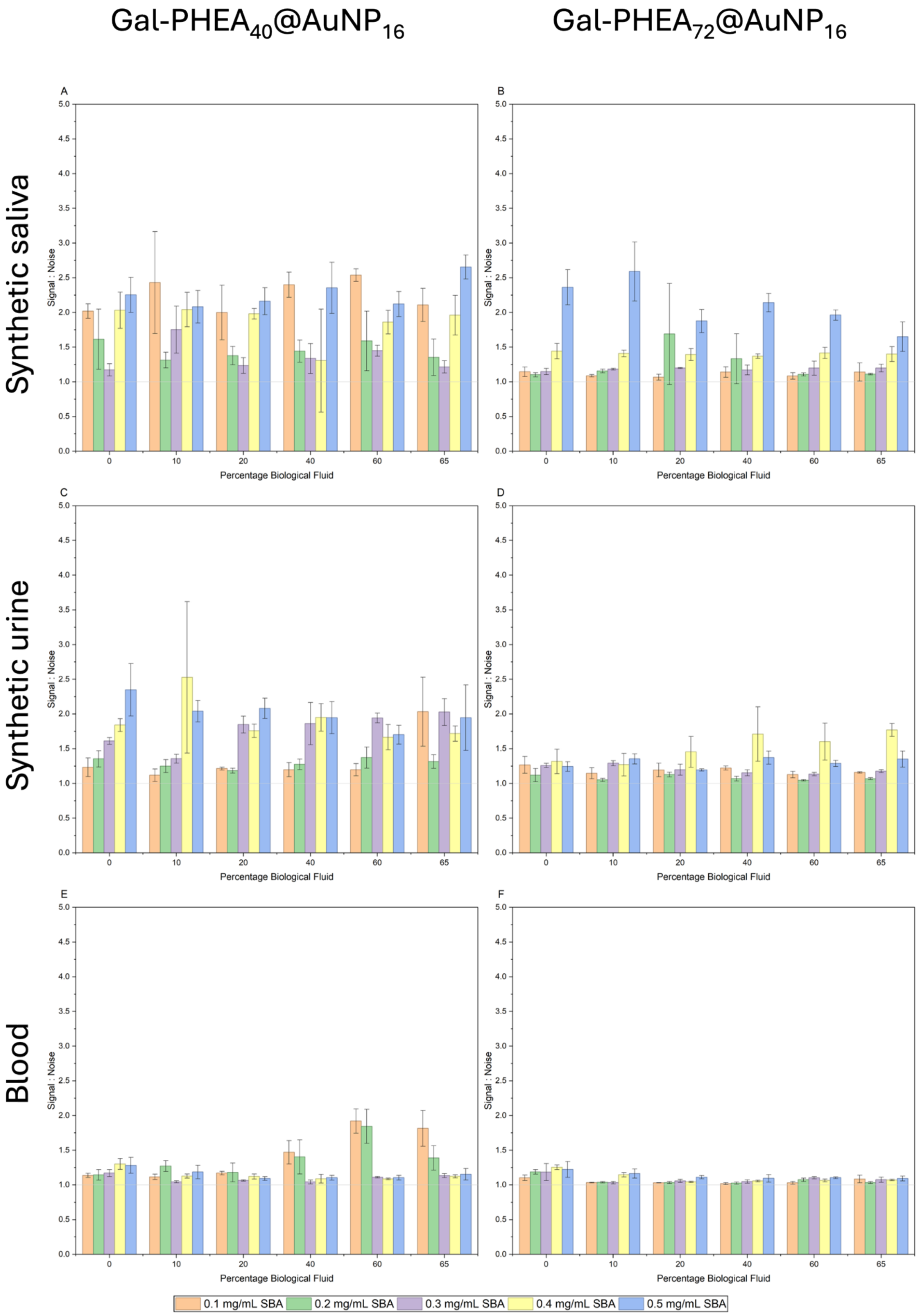

Figure S32. – Signal to noise analysis of doped buffers at varying percentages of media, and varying SBA concentrations of 0.1 mg/mL to 0.5 mg/mL. A) Gal-PHEA$_{40}$@AuNP$_{16}$ in saliva, B) Gal-

$PHEA_{72}$@$AuNP_{16}$ in saliva, C) Gal-$PHEA_{40}$@$AuNP_{16}$ in urine, D) Gal-$PHEA_{72}$@$AuNP_{16}$ in urine, E) Gal-$PHEA_{40}$@$AuNP_{16}$ in blood, and F) Gal-$PHEA_{72}$@$AuNP_{16}$ in blood.

Lateral Flow Test Results

Percentage %	Saliva		Urine		Blood	
	SBA	BSA	SBA	BSA	SBA	BSA
0	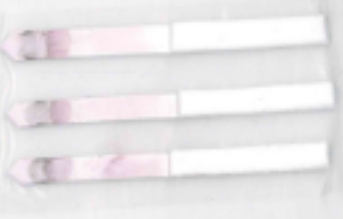	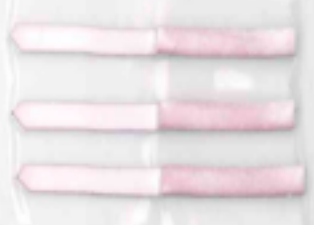	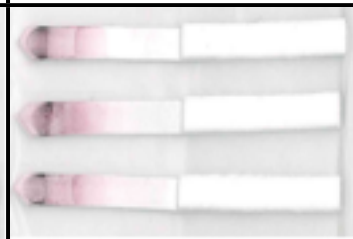	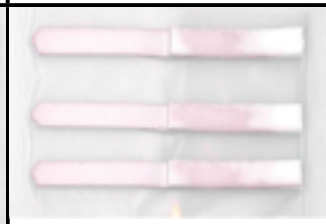	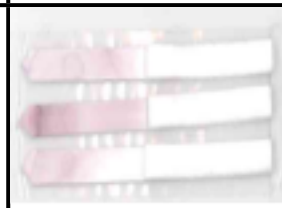	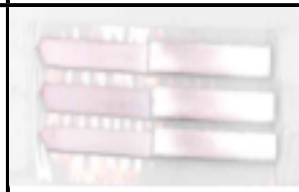
10	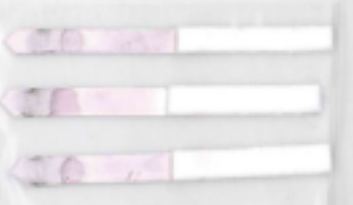	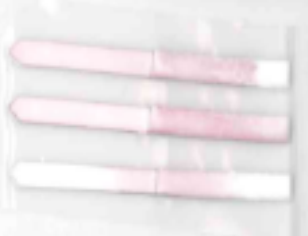	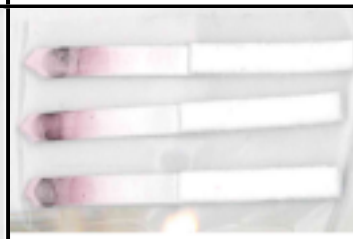	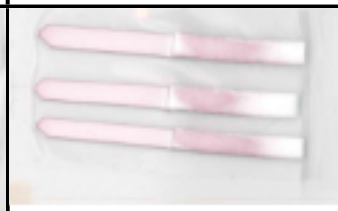	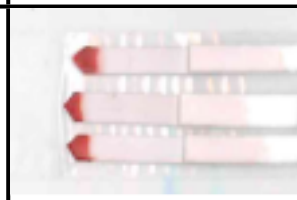	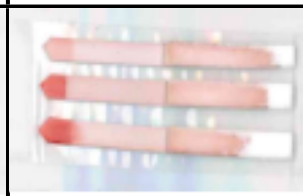
20	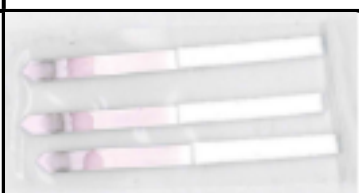	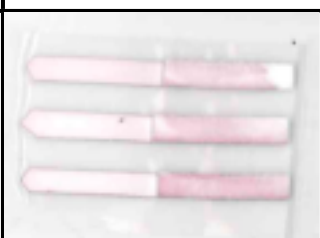	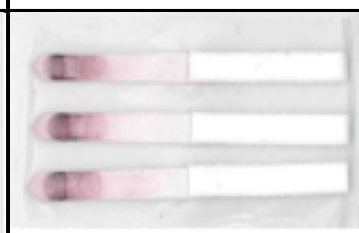	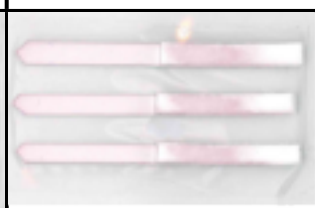	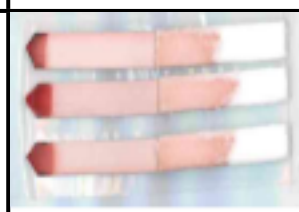	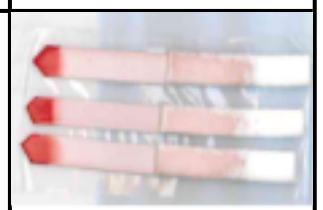
40	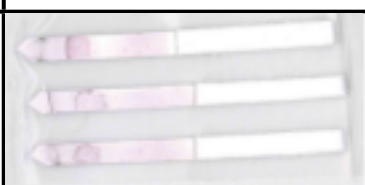	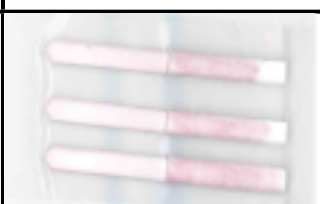	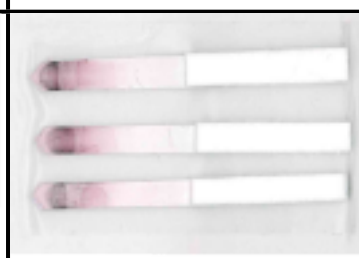	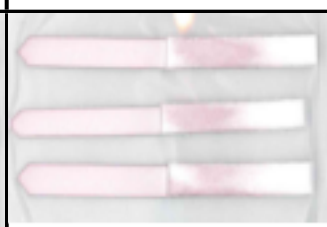	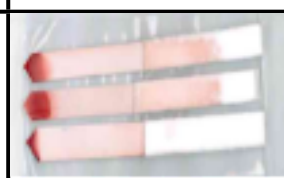	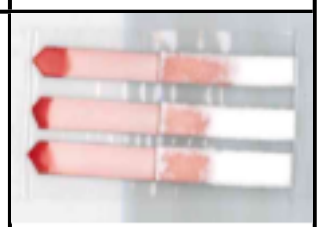
60	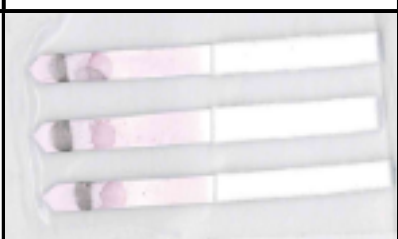	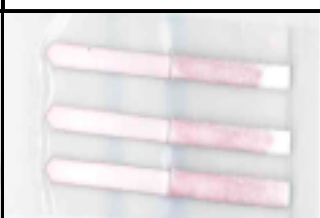	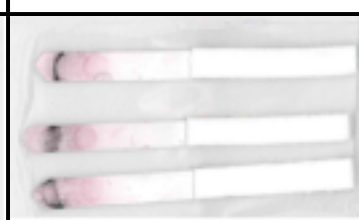	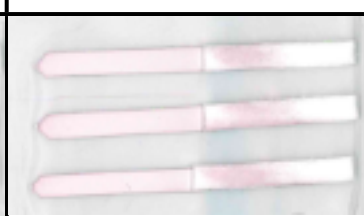	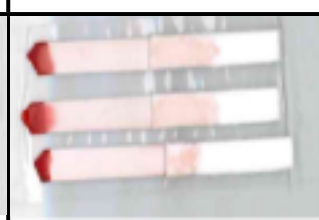	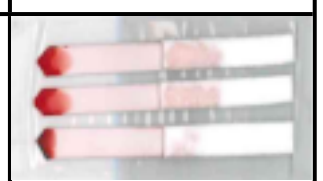

Table S15. – Scan of lateral flow strips using Gal-PHEA$_{40}$@AuNP$_{16}$ (OD=4) in doped buffers (varying concentrations of dopant saliva, urine and blood), and 0.1 mg/mL SBA or BSA in buffer, with a 0.5 mg/mL test line of Galɑ1-3Galβ1-4GlcNAc-BSA.

Percentage%	Saliva		Urine		Blood	
	SBA	BSA	SBA	BSA	SBA	BSA
0	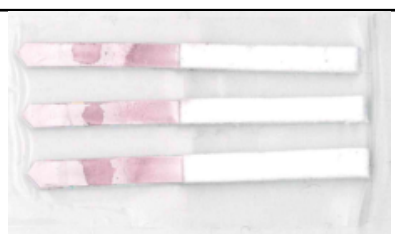	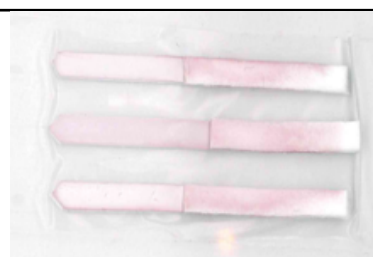	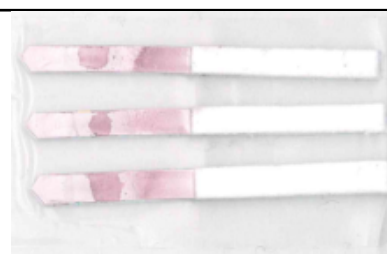	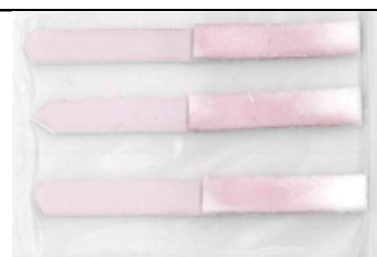	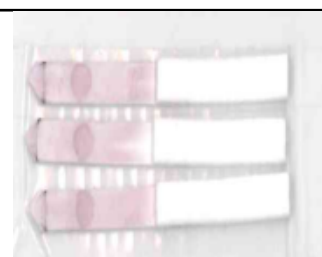	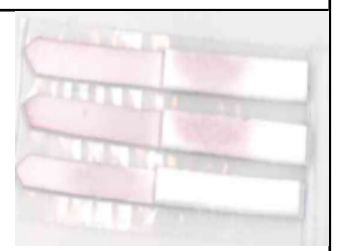
10	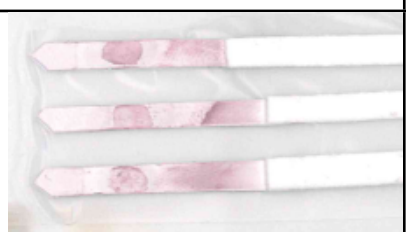	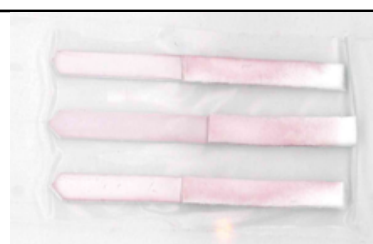	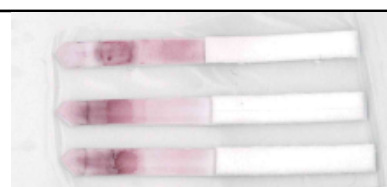	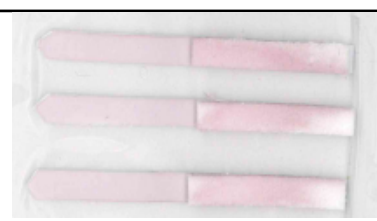	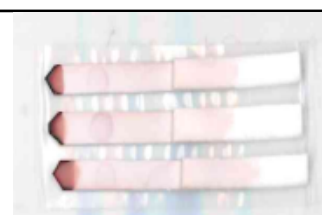	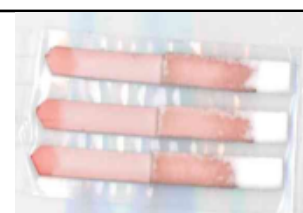
20	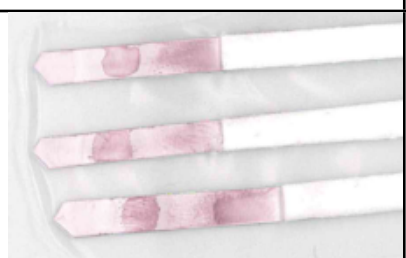	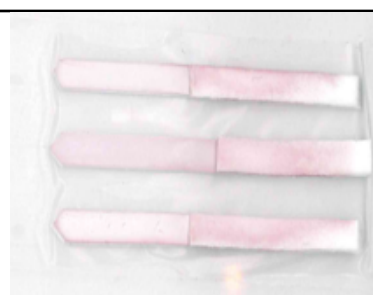	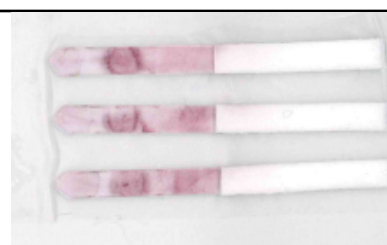	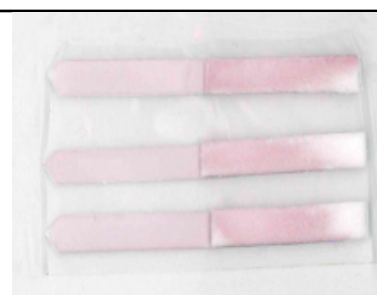	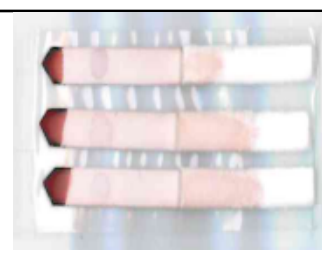	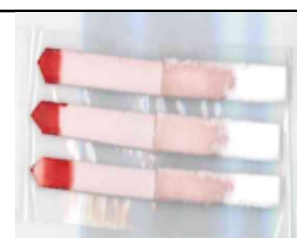
40	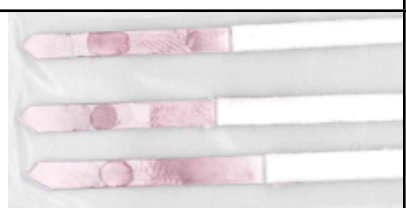	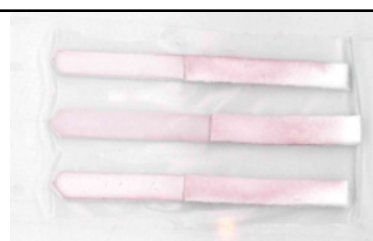	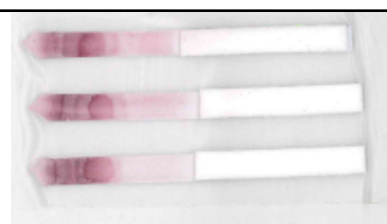	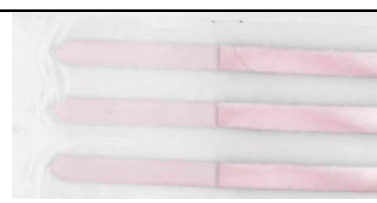	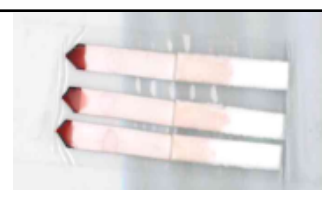	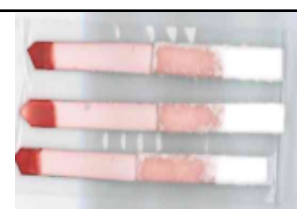
60	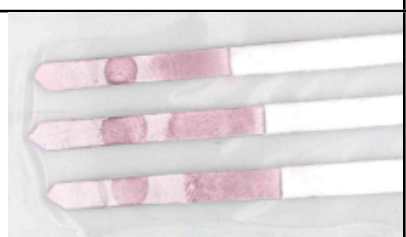	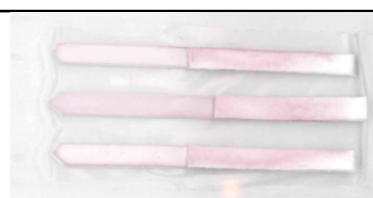	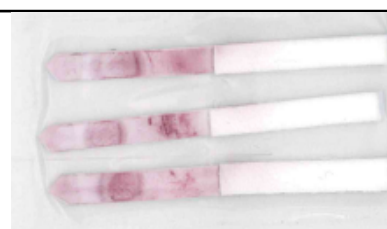	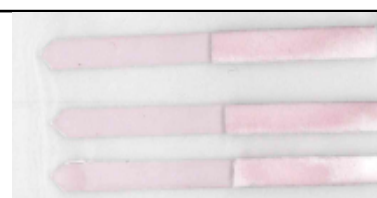	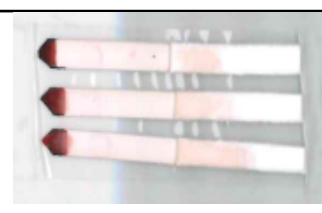	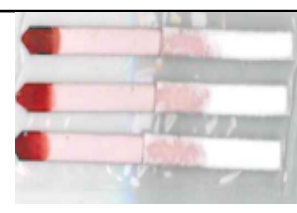

Table S16. – Scan of lateral flow strips using Gal-PHEA$_{72}$@AuNP$_{16}$ (OD=4) in doped buffers (varying concentrations of dopant saliva, urine and blood), and 0.1 mg/mL SBA or BSA in buffer, with a 0.5 mg/mL test line of Galɑ1-3Galβ1-4GlcNAc-BSA.

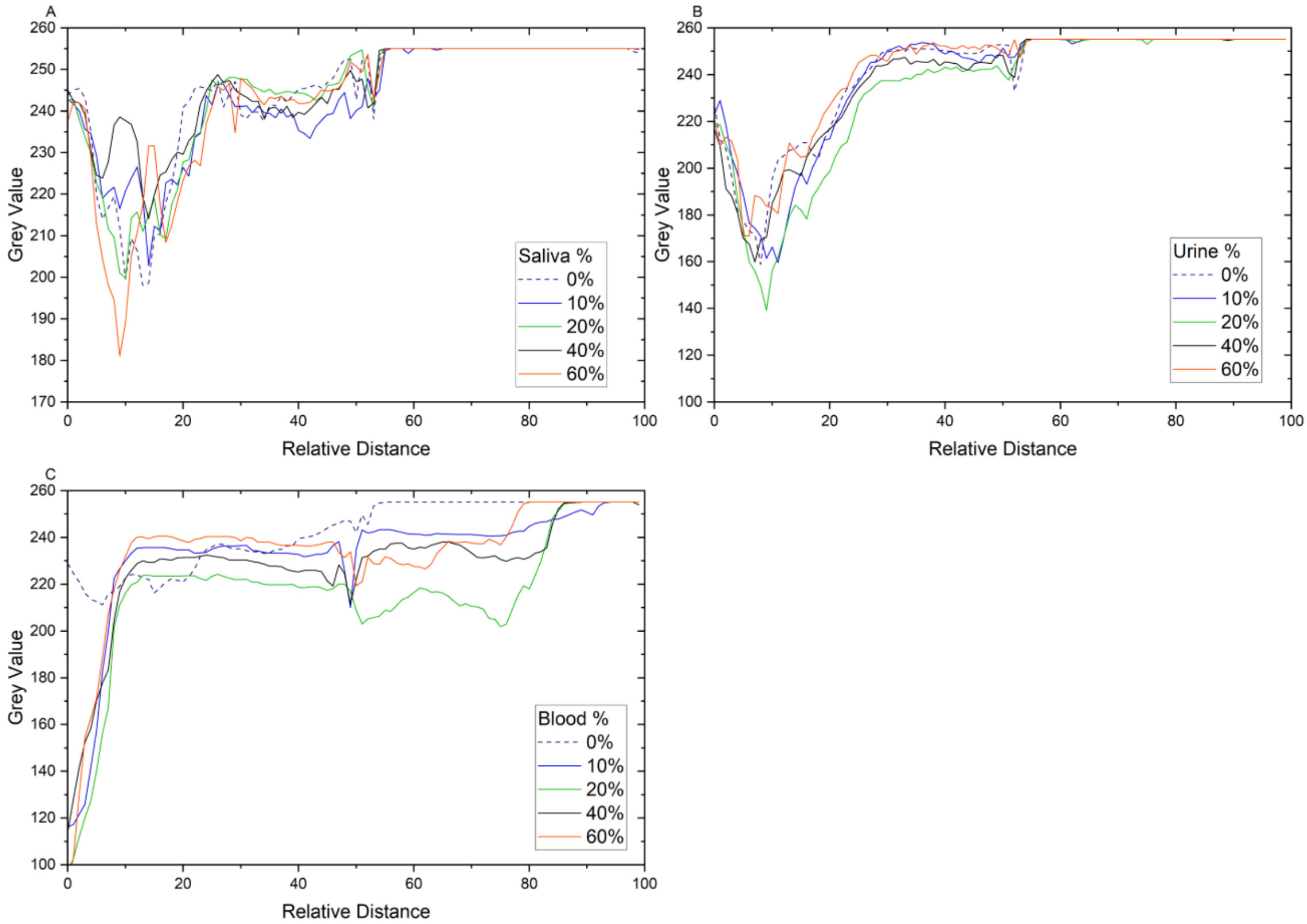

Figure S33. – Analysis of scanned lateral flow strips using Gal-PHEA$_{40}$@AuNP$_{16}$, in doped buffers (varying concentrations of dopant (A) saliva, (B) urine and (C) blood), and 0.1 mg/mL SBA in buffer, with a 0.5 mg/mL test line of Galɑ1-3Galβ1-4GlcNAc-BSA.

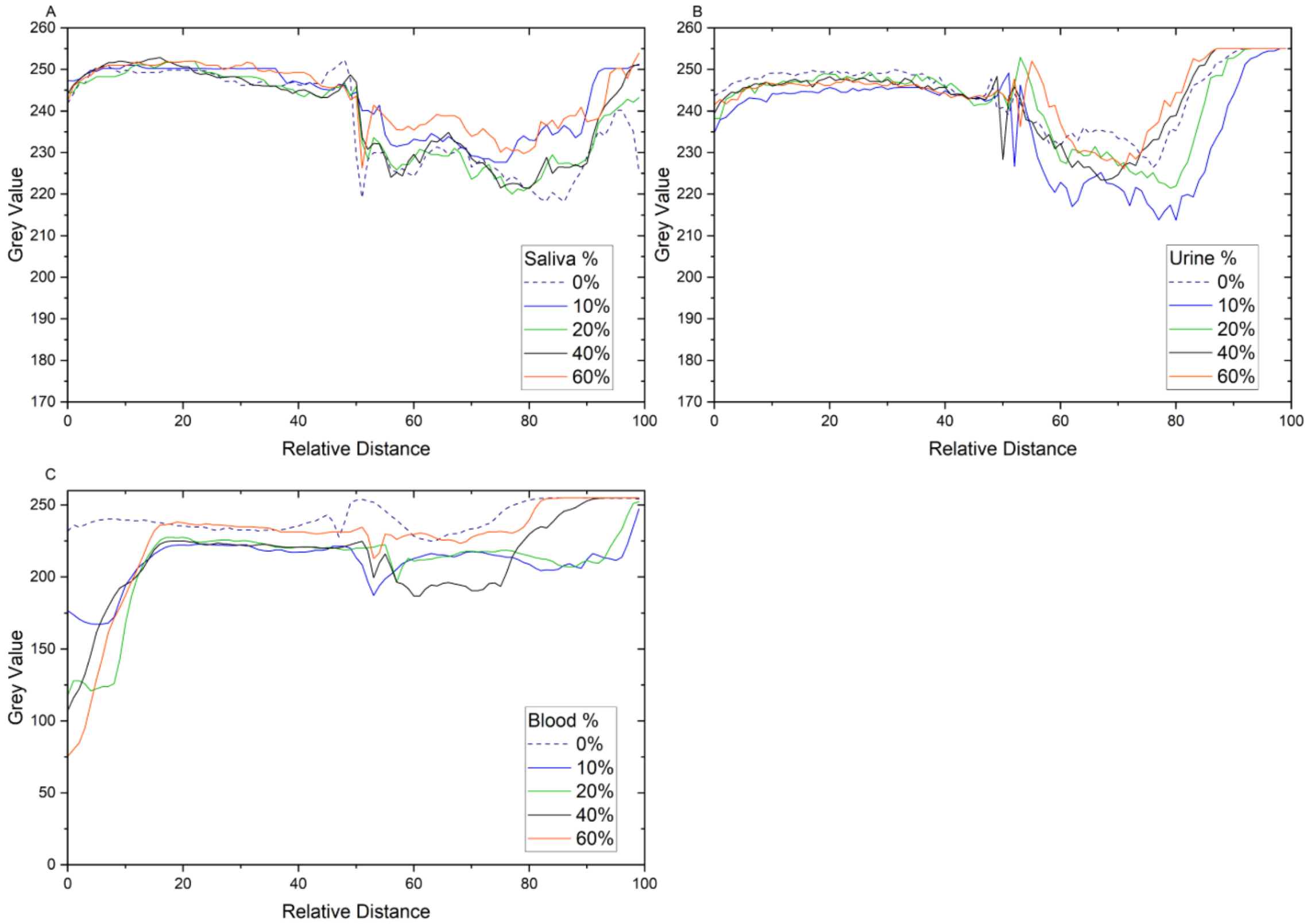

Figure S34. – Analysis of scanned lateral flow strips using Gal-PHEA$_{40}$@AuNP$_{16}$, in doped buffers (varying concentrations of dopant (A) saliva, (B) urine and (C) blood), and 0.1 mg/mL BSA in buffer, with a 0.5 mg/mL test line of Galɑ1-3Galβ1-4GlcNAc-BSA.

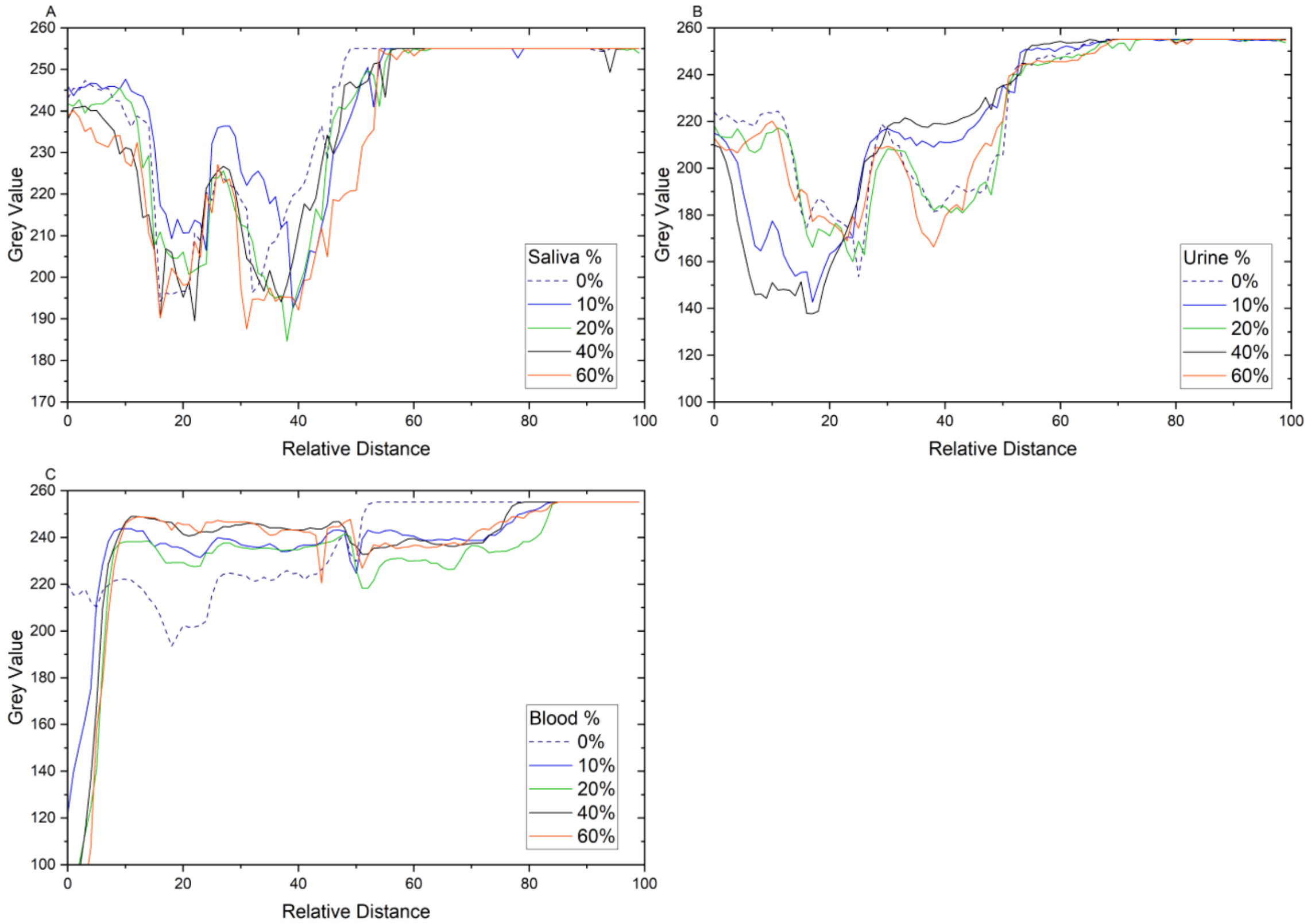

Figure S35. – Analysis of scanned lateral flow strips using Gal-PHEA$_{72}$@AuNP$_{16}$, in doped buffers (varying concentrations of dopant (A) saliva, (B) urine and (C) blood), and 0.1 mg/mL SBA in buffer, with a 0.5 mg/mL test line of Galɑ1-3Galβ1-4GlcNAc-BSA.

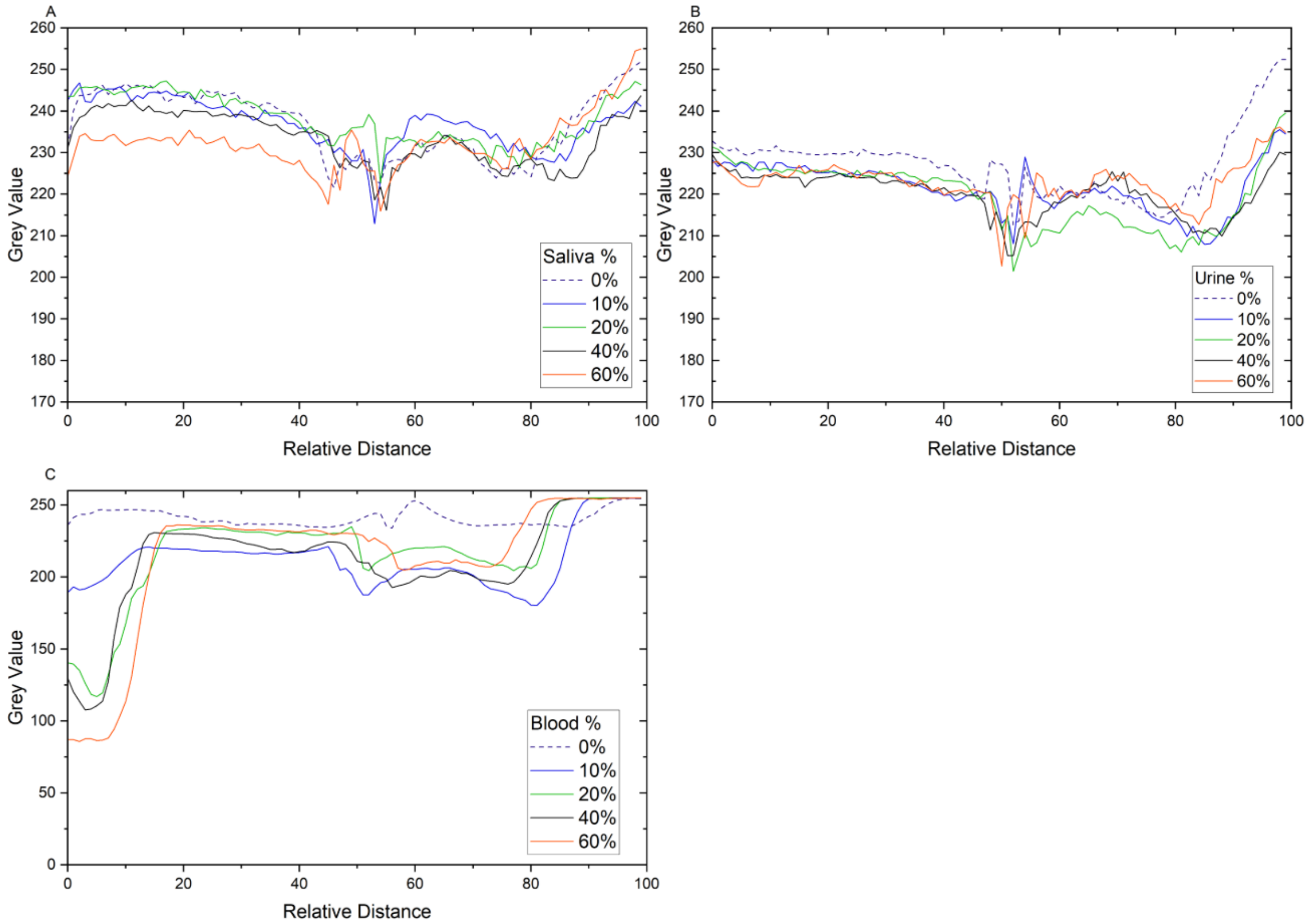

Figure S36. – Analysis of scanned lateral flow strips using Gal-PHEA$_{72}$@AuNP$_{16}$, in doped buffers (varying concentrations of dopant (A) saliva, (B) urine and (C) blood), and 0.1 mg/mL BSA in buffer, with a 0. 5 mg/mL test line of Galɑ1-3Galβ1-4GlcNAc-BSA.

Signal to Noise Results for Lateral Flow

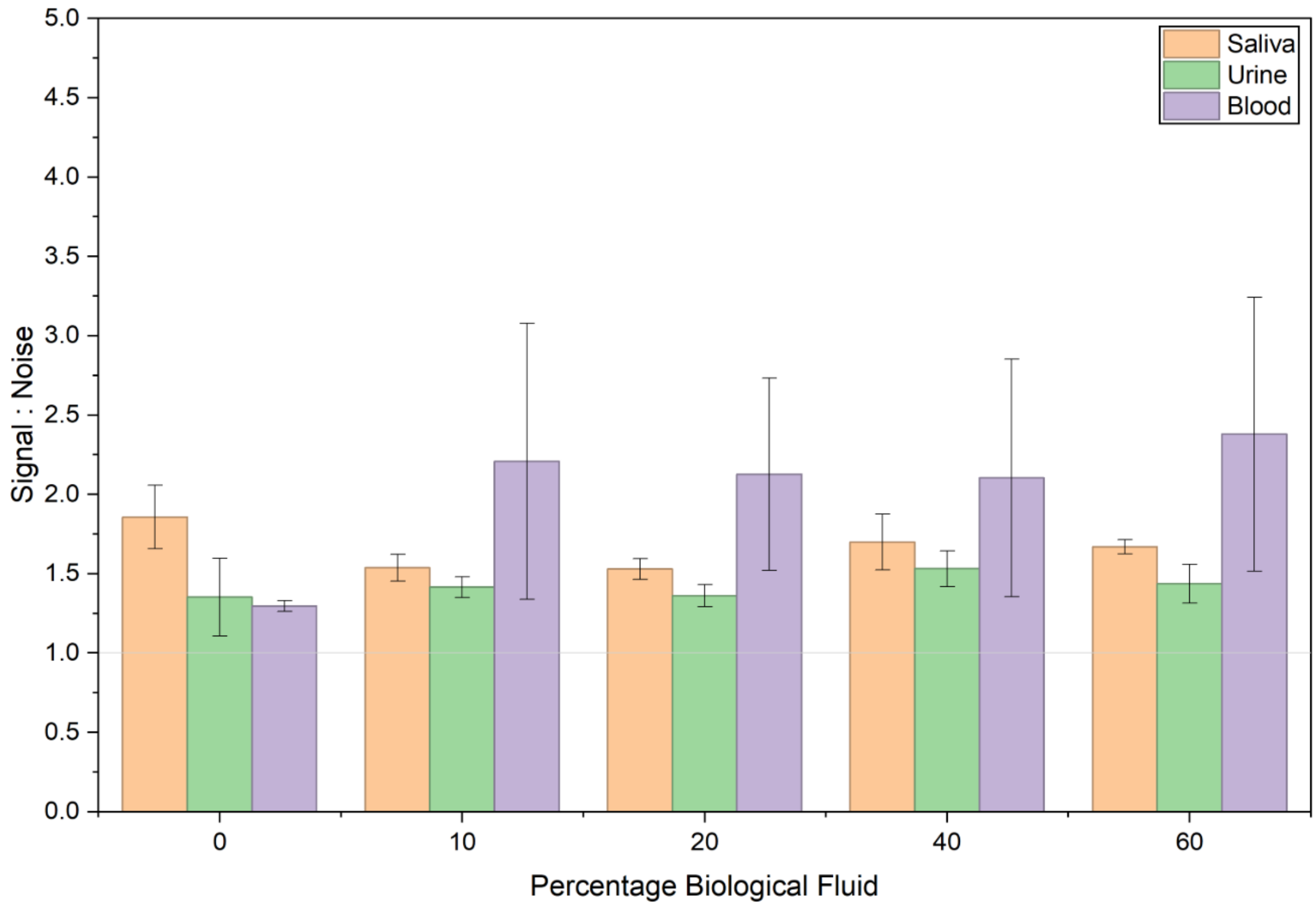

Figure S37. – Signal to noise analysis of Gal-PHEA$_{40}$@AuNP$_{16}$, and SBA concentrations of 0.1 mg/mL in doped buffers (varying concentrations of dopant saliva, urine and blood), with a 0.5 mg/mL test line of Galɑ1-3Galβ1-4GlcNAc-BSA.

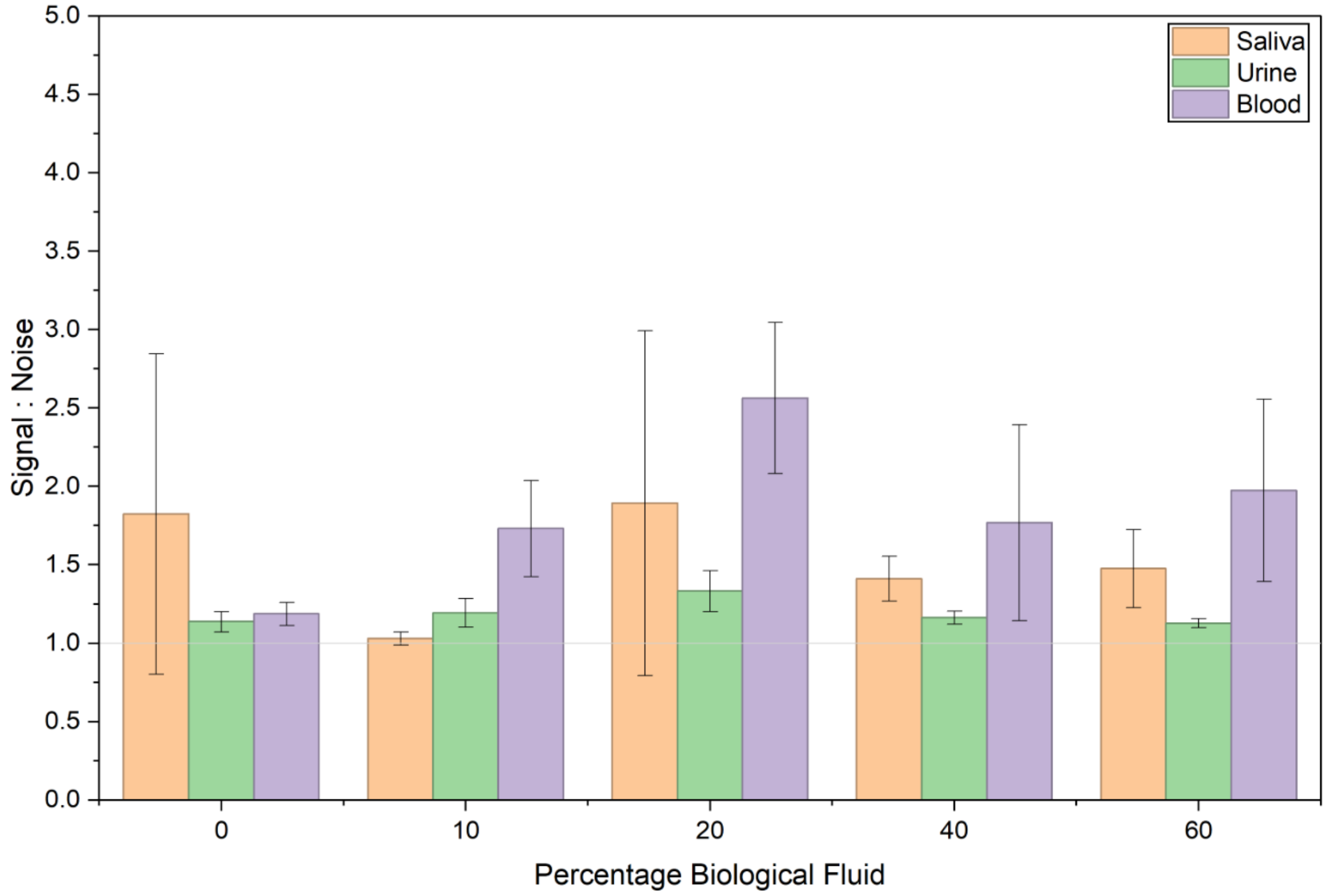

Figure S38. – Signal to noise analysis of Gal-PHEA$_{40}$@AuNP$_{16}$, and BSA concentrations of 0.1 mg/mL in doped buffers (varying concentrations of dopant saliva, urine and blood), with a 0.5 mg/mL test line of Galɑ1-3Galβ1-4GlcNAc-BSA.

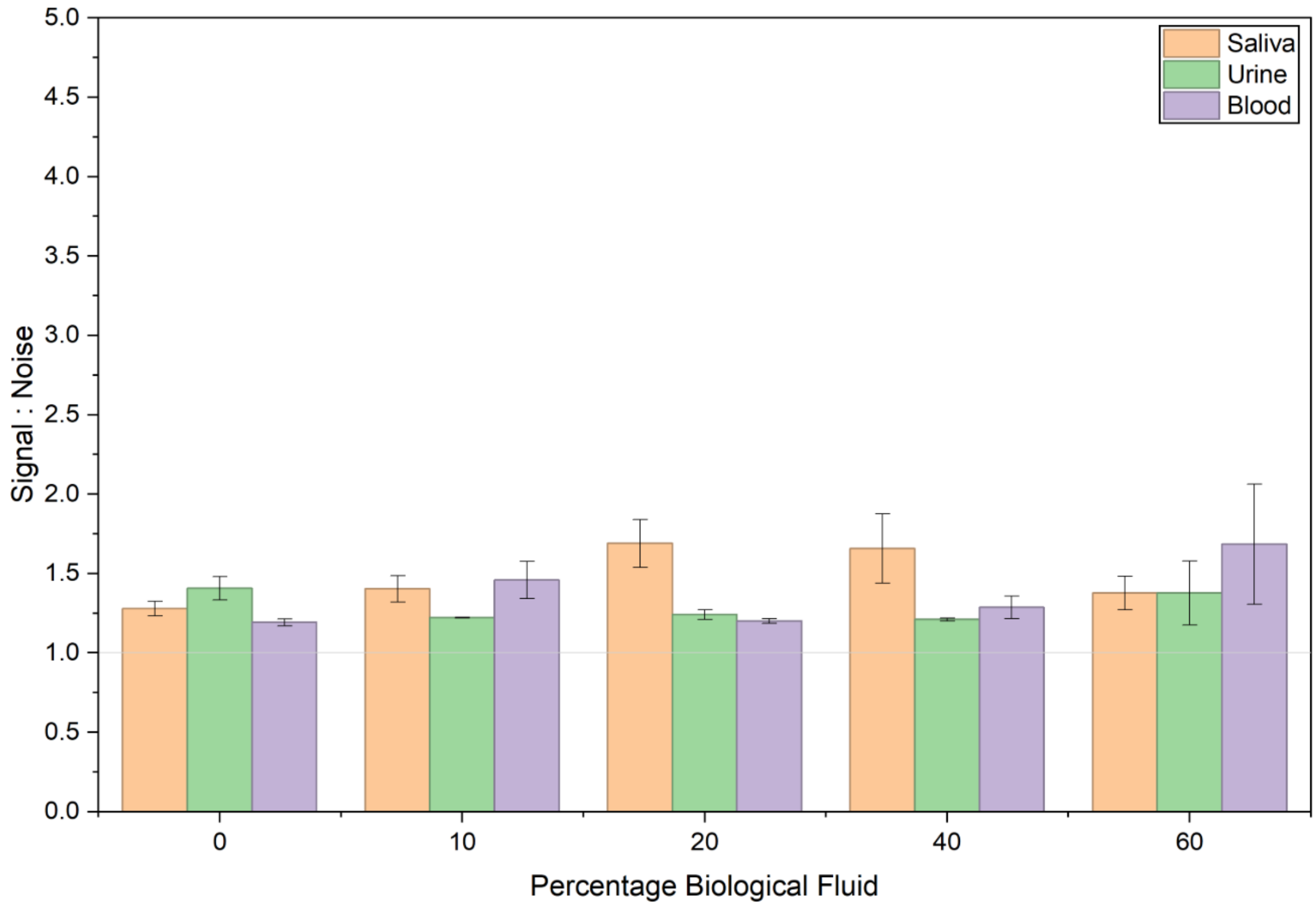

Figure S39. – Signal to noise analysis of Gal-PHEA$_{72}$@AuNP$_{16}$, and SBA concentrations of 0.1 mg/mL in doped buffers (varying concentrations of dopant saliva, urine and blood), with a 0.5 mg/mL test line of Galɑ1-3Galβ1-4GlcNAc-BSA.

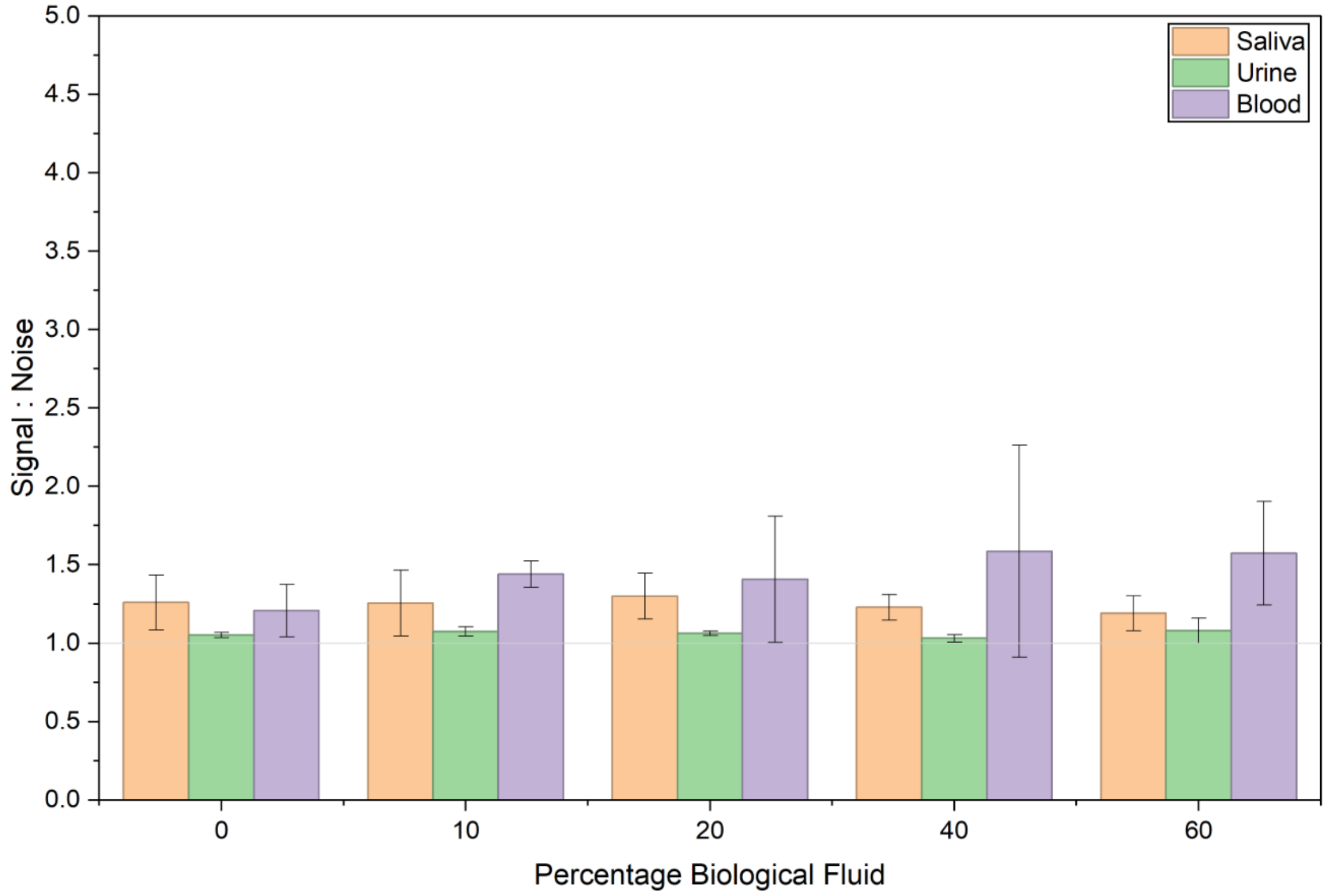

Figure S40. – Signal to noise analysis of Gal-PHEA$_{72}$@AuNP$_{16}$, and BSA concentrations of 0.1 mg/mL in doped buffers (varying concentrations of dopant saliva, urine and blood), with a 0.5 mg/mL test line of Galɑ1-3Galβ1-4GlcNAc-BSA.

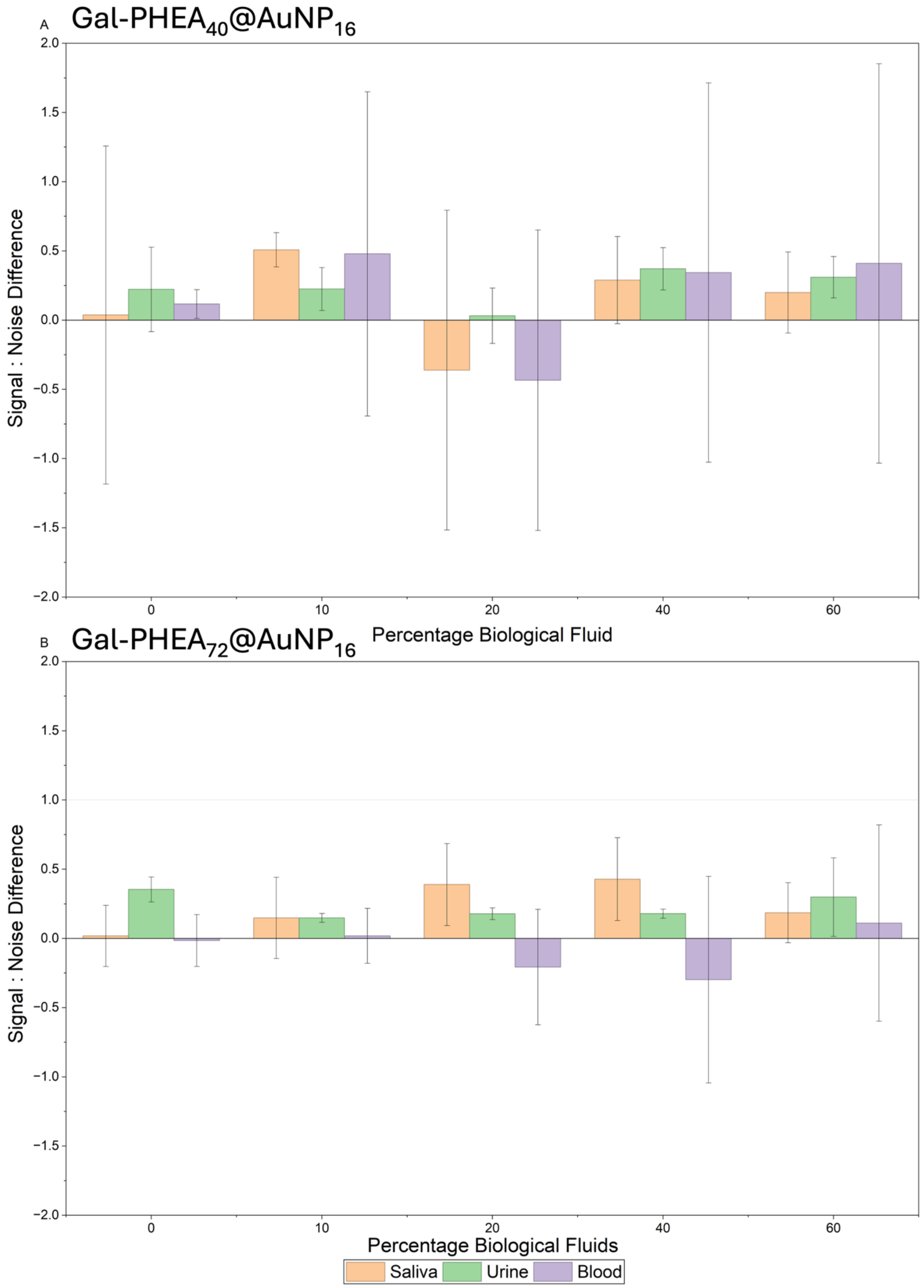
A
Gal-PHEA40@AuNP16
Signal : Noise Difference
2.0
1.5
1.0
0.5
0.0
−0.5
−1.0
−1.5
−2.0
0
10
20
40
60
Percentage Biological Fluid
B
Gal-PHEA72@AuNP16
Signal : Noise Difference
2.0
1.5
1.0
0.5
0.0
−0.5
−1.0
−1.5
−2.0
0
10
20
40
60
Percentage Biological Fluids
Saliva
Urine
Blood

Figure S41. – Signal to noise analysis (SBA SNR – BSA SNR), SBA concentrations of 0.1 mg/mL in doped buffers (varying concentrations of dopant saliva, urine and blood), with a 0.5 mg/mL test line of Galɑ1-3Galβ1-4GlcNAc-BSA. A) Gal-PHEA$_{40}$@AuNP$_{16}$. B) Gal-PHEA$_{72}$@AuNP$_{16}$.

References

1. Baker, A. N.; Richards, S.-J.; Guy, C. S.; Congdon, T. R.; Hasan, M.; Zwetsloot, A. J.; Gallo, A.; Lewandowski, J. R.; Stansfeld, P. J.; Straube, A.; Walker, M.; Chessa, S.; Pergolizzi, G.; Dedola, S.; Field, R. A.; Gibson, M. I. The SARS-COV-2 Spike Protein Binds Sialic Acids and Enables Rapid Detection in a Lateral Flow Point of Care Diagnostic Device. *ACS Central Science* **2020**, *6* (11), 2046–2052.
2. Bastús, N. G.; Comenge, J.; Puntes, V. Kinetically Controlled Seeded Growth Synthesis of Citrate-Stabilized Gold Nanoparticles of up to 200 Nm: Size Focusing versus Ostwald Ripening. *Langmuir* **2011**, *27* (17), 11098–11105.
3. Haiss, W.; Thanh, N. T. K.; Aveyard, J.; Fernig, D. G. Determination of Size and Concentration of Gold Nanoparticles from UV - Vis Spectra. *Analytical Chemistry* **2007**, *79* (11), 4215–4221.
4. Baker, A. N.; Muguruza, A. R.; Richards, S. J.; Georgiou, P. G.; Goetz, S.; Walker, M.; Dedola, S.; Field, R. A.; Gibson, M. I. Lateral Flow Glyco-Assays for the Rapid and Low-Cost Detection of Lectins–Polymeric Linkers and Particle Engineering Are Essential for Selectivity and Performance. *Advanced healthcare materials* **2022**, *11* (4), 2101784.
